# New Acyl Derivatives of 3-Aminofurazanes and Their Antiplasmodial Activities

**DOI:** 10.3390/ph14050412

**Published:** 2021-04-27

**Authors:** Theresa Hermann, Patrick Hochegger, Johanna Dolensky, Werner Seebacher, Robert Saf, Marcel Kaiser, Pascal Mäser, Robert Weis

**Affiliations:** 1 Institute of Pharmaceutical Sciences, Pharmaceutical Chemistry, University of Graz, Schubertstraße 1, A-8010 Graz, Austria; theresa.hermann@uni-graz.at (T.H.); johanna.faist@uni-graz.at (J.D.); we.seebacher@uni-graz.at (W.S.); robert.weis@uni-graz.at (R.W.); 2 Institute for Chemistry and Technology of Materials (ICTM), Graz University of Technology, Stremayrgasse 9, A-8010 Graz, Austria; robert.saf@tugraz.at; 3 Swiss Tropical and Public Health Institute, Socinstraße 57, CH-4002 Basel, Switzerland; marcel.kaiser@swisstph.ch (M.K.); pascal.maeser@swisstph.ch (P.M.)

**Keywords:** antimalarial, furazan derivatives, *Plasmodium falciparum*, PAMPA

## Abstract

An *N*-acylated furazan-3-amine of a Medicines for Malaria Venture (MMV) project has shown activity against different strains of *Plasmodium falciparum*. Seventeen new derivatives were prepared and tested in vitro for their activities against blood stages of two strains of *Plasmodium falciparum*. Several structure–activity relationships were revealed. The activity strongly depended on the nature of the acyl moiety. Only benzamides showed promising activity. The substitution pattern of their phenyl ring affected the activity and the cytotoxicity of compounds. In addition, physicochemical parameters were calculated (log P, log D, ligand efficiency) or determined experimentally (permeability) via a PAMPA. The *N*-(4-(3,4-diethoxyphenyl)-1,2,5-oxadiazol-3-yl)-3-(trifluoromethyl)benzamide possessed good physicochemical properties and showed high antiplasmodial activity against a chloroquine-sensitive strain (IC_50_(NF54) = 0.019 µM) and even higher antiplasmodial activity against a multiresistant strain (IC_50_(K_1_) = 0.007 µM). Compared to the MMV compound, the permeability and the activity against the multiresistant strain were improved.

## 1. Introduction

Malaria is, to this day, one of the most dangerous infectious diseases worldwide. It is caused by protozoa of the genus *Plasmodium*. An estimated number of 229 million cases occurred in 2019, resulting in more than 400,000 deaths. The burden of this infection is mostly carried by children under the age of five located in sub-Saharan Africa. Five *Plasmodium* species are human pathogenic: *P. falciparum*, *P. vivax*, *P. malariae*, *P. ovale* and *P. knowlesi* of which *P. falciparum* is the most predominant cause of death [1,2]. The discovery of chloroquine in the 1960s had an enormous impact on the fight against malaria. Increasing resistance development in *P. falciparum*, however, was reported more than fifty years ago, and the cost of it in human lives was severe [3,4]. The first-line treatment for malaria infections is an artemisinin-based combination therapy (ACT). A rapid-acting artemisinin derivative is combined with an antimalarial drug with a long half-life period. However, artemisinin drug resistances are increasingly emerging in *P. falciparum*, especially within the Southeast Asian region, and are therefore jeopardizing the success of ACTs [5,6]. A new strategy to impede the increasing resistance development temporarily is the application of triple artemisinin-based combination therapy (TACT) [7,8]. Due to the threat of potentially untreatable *P. falciparum* malaria, the development of drugs with new modes of action is of utmost importance [9,10,11].

Phenotypic screening or whole-cell screening represents a significant breakthrough in the discovery of new antimalarial lead structures. The majority of compounds currently in clinical trials were discovered by means of this screening method. The benefit of phenotypic screening is that the activity is not determined at isolated targets, but in the physiological environment. The implementation of this method led to a 100-fold reduction in the cost for screening compounds against *P. falciparum*-infected erythrocytes compared to other methods. Most commonly, the target search starts afterward [12,13].

In 2016, the organization Medicines for Malaria Venture (MMV) published the results of a major phenotypic screening project. The so-called Malaria Box contains 400 substances with activity against *P. falciparum* and serves as a starting point for the development of new lead structures. Alongside activity assays against different strains and life cycle stages of *P. falciparum*, their cytotoxicity against 73 human cell lines was determined as well [14].

**Scheme 1:**
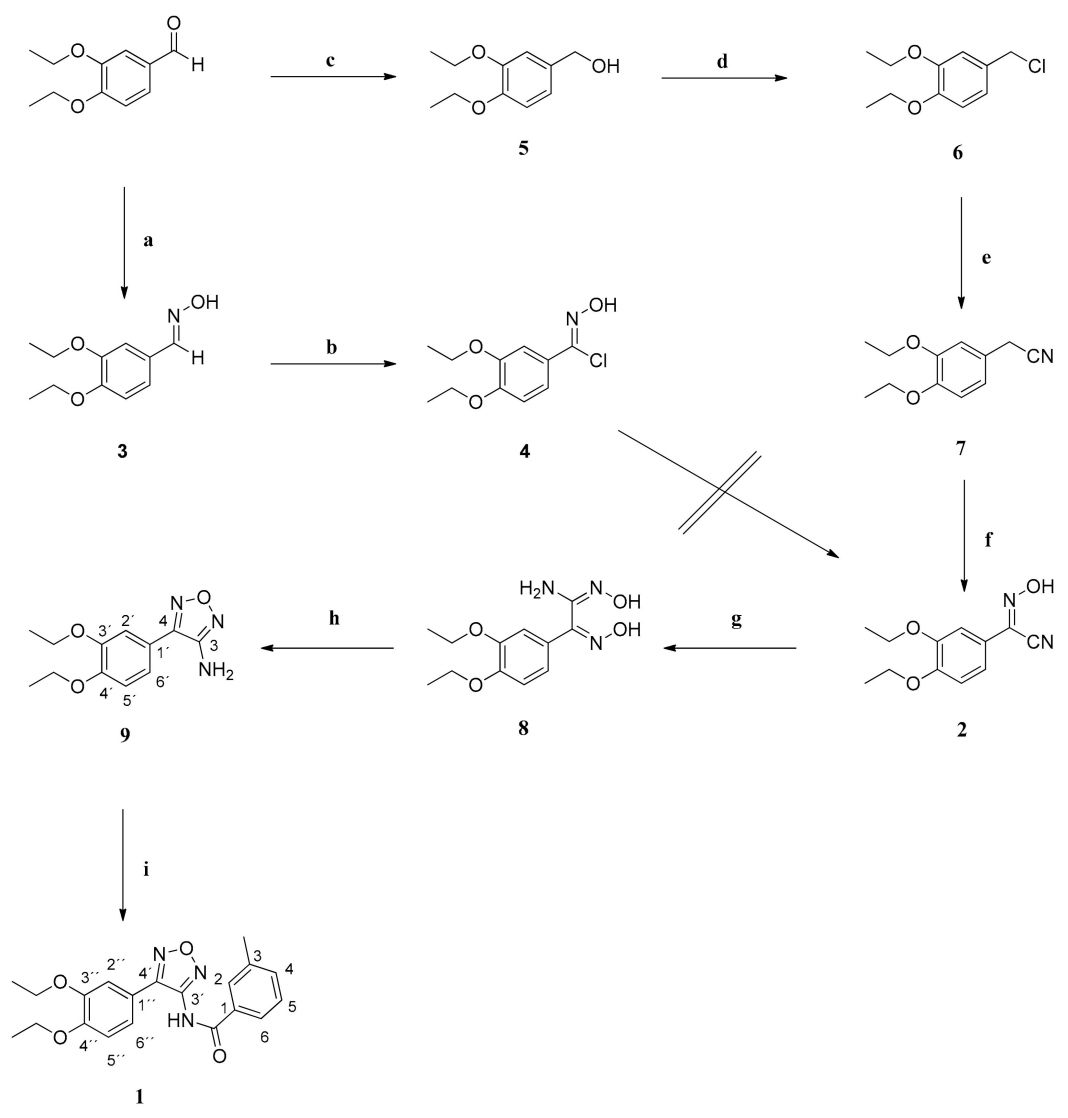
Preparation of compound 1. Reagents and conditions: (**a**) NH_2_OH x HCl, NaHCO_3_, MeOH, H_2_O, 100 °C, 2 h; (**b**) *N*-chlorosuccinimide, DMF, rt, 20 h; (**c**) NaBH_4_, MeOH, rt, 1 h; (**d**) thionyl chloride, CH_2_Cl_2_, rt, 20 h; (**e**) KCN, DMF, rt, 4 h; (**f**) (1) NaOEt, 0 °C, 30 min; (2) 3-methylbutyl nitrite, EtOH, rt, 20 h; (**g**) NH_2_OH x HCl, NaHCO_3_, MeOH, H_2_O, 100 °C, 20 h; (**h**) 2*N* NaOH, 100 °C, 20 h; (**i**) (1) NaH, DMF, 0 °C, 20 min; (2) 3-methylbenzoyl chloride, DMF, 60 °C, 48 h.

The furazan **1** (Scheme 1) from MMV’s Malaria Box project is a promising lead structure for the development of new antimalarials. It shows antiplasmodial activity against the multiresistant strains K_1_ and Dd2 of *Plasmodium falciparum* [14]. Furthermore, a *P. berghei* assay revealed activity against liver schizonts [15,16]. The potential of inhibiting the transmission from humans to mosquitoes by working against early gametocyte stages makes this compound even more promising [17,18,19,20,21].

Within two subsequent studies, the possible targets of furazan **1** could be identified. Firstly, it is likely to inhibit the Na^+^-efflux pump *Pf*ATP4, which is essential in maintaining the parasite’s ion homeostasis. *Pf*ATP4 is localized on the plasma membrane of *P. falciparum* and represents an attractive target for novel antimalarials. However, genetic *Pf*ATP4 mutations led to an increase in resistance development against several preclinical and clinical antimalarials. The furazan **1** also interacts with the enzyme deoxyhypusine hydroxylase (DOHH) which is part of the hypusine biosynthesis [22,23,24,25].

The aim of this study was to synthesize new derivatives of compound **1** in order to increase the antiplasmodial activity and reveal structure–activity relationships (SARs). All newly synthesized compounds were characterized and tested in vitro for their activities against the chloroquine-sensitive strain NF54 and the multiresistant K_1_ strain of *P. falciparum*. The results were compared to those of drugs in use. Furthermore, the passive diffusion of the compounds was determined by a parallel artificial membrane permeability assay (PAMPA) to classify compounds according to their permeability. Especially in the development of new antimalarials, it is important to focus on substances that are orally bioavailable, because of the potential storage problems with other formulations in countries where malaria is prevalent and the simpler use. The PAMPA is an early screening assay to differentiate between compounds that have a good oral absorption potential and those that do not.

## 2. Results and Discussion

### 2.1. Chemistry

The precursor of all derivatives, compound **1**, was synthesized from 3,4-diethoxybenzaldehyde in a multistep procedure via the formation of a cyanide-substituted oxime **2**. Since no synthetic route to generate compound **1** has been published yet, we used a retrosynthetic approach. Two different methods were applied for the synthesis of the oxime **2**. Treatment of 3,4-diethoxybenzaldehyde with hydroxylamine hydrochloride gave the aldoxime **3** in high yields. Conversion of the benzaldoxime to the benzimidoyl chloride **4** succeeded with *N*-chloro succinimide. However, subsequent reaction with potassium cyanide to afford compound **2** failed. Therefore, an alternative method was used, and compound **1** was successfully prepared in a seven-step procedure.

At first, 3,4-diethoxybenzaldehyde was reduced to its corresponding alcohol **5** using sodium borohydride in methanol. The successful reduction was obvious by the disappearance of the signal of the formyl proton in the ^1^H NMR spectrum. A resonance at ca. 4.5 ppm appeared for the new methylene group. Treatment with thionyl chloride gave the benzyl chloride **6**. Due to the replacement of the hydroxy group by a chlorine atom the ^13^C resonance of the methylene group was shifted 18 ppm upfield. It was then converted into the 2-phenylacetonitrile **7** by means of a Kolbe nitrile synthesis [26]. The replacement of the chlorine atom by a cyano group shifted the proton signal of the methylene group 1 ppm upfield. Its α-protons were replaced by a hydroximino group. After deprotonation with sodium ethylate, 3-methylbutyl nitrite was added, yielding the desired oxime **2** [27]. The oxime carbon gave a new signal at ca. 133 ppm in the ^13^C NMR spectrum, whereas the resonance of the methylene group was missing. It was treated with hydroxylamine hydrochloride giving the amide oxime **8**. The conversion of the cyano group to an amidoxime shifted the signal of the concerned carbon atom 35 ppm downfield in the ^13^C NMR spectrum. Refluxing of **8** with 2*N* NaOH led to ring closure, affording the 3-aminofurazan **9**. Due to the formation of the furazan ring system, the signals of both hydroxy groups disappeared in the ^1^H NMR spectrum. The resonance of the amino protons was shifted 0.5 ppm downfield. The desired compound **1** was finally obtained by reaction of **9** with sodium hydride and 3-methylbenzoyl chloride in DMF (Scheme 1) [28]. The successful amide bond formation was detected by a significant change in the NMR spectrum. The signal of the aromatic amino protons was replaced by a broadened signal at high frequencies.

To obtain some insight considering structure–activity relationship, the amino furazan **9** was subsequently coupled with different carboxylic acids. Furthermore, the importance of the *meta*-methyl group was investigated. Compounds **10**–**17** and **26**–**29** were synthesized.

**Scheme 2:**
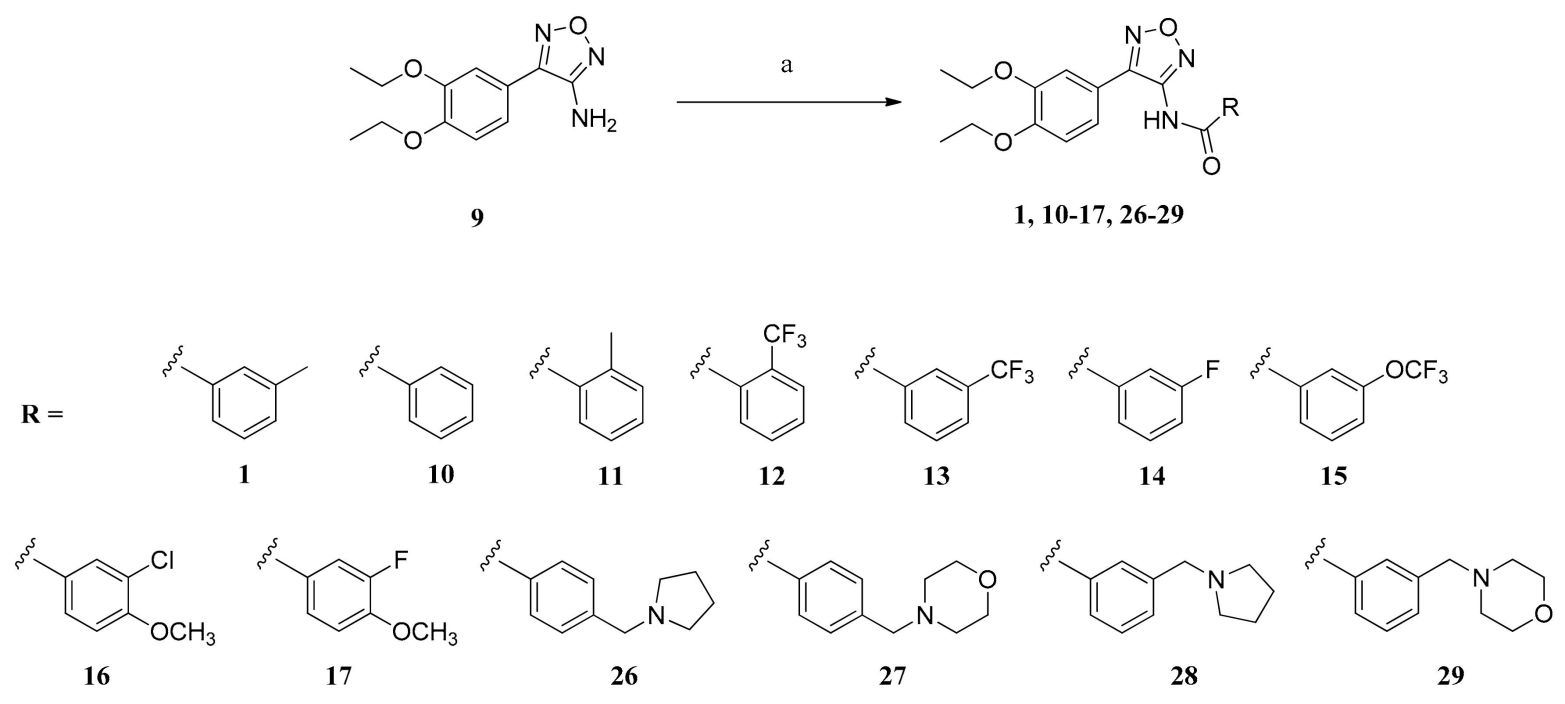
Preparation of compounds **1**, **10**–**17** and **26**–**29**. Reagents and conditions: (**a**) (1) NaH, DMF, 0 °C, 20 min; (2) acyl chloride, DMF, 60 °C, 48 h (compounds **1** and **10**–**14)**; or (1) carboxylic acid, oxalyl dichloride, CH_2_Cl_2_, rt, 20 h; (2) NaH, DMF, 0 °C, 20 min; (3) acid chloride, DMF, 60 °C, 48 h (compounds **16**, **17** and **26**–**29**); or (1) carboxylic acid, *N*-hydroxy succinimide, DCC, THF, rt, 24 h; (2) DMF, rt, 15 min; (3) NaH, DMF, 0 °C, 20 min; (4) acid chloride, DMF, 60 °C, 48 h (compound **15**).

The amides **1**, **10**–**14**, **16**, **17** and **26**–**29** were obtained by coupling the amino furazan **9** with the respective benzoyl chlorides that were commercially available or generated by the reaction of benzoic acids and oxalyl dichloride (Scheme 2) [28,29]. Reaction of the *N*-hydroxy succinimide ester of the 3-(trifluoromethoxy)benzoic acid with **9** yielded **15** [30].

**Scheme 3:**
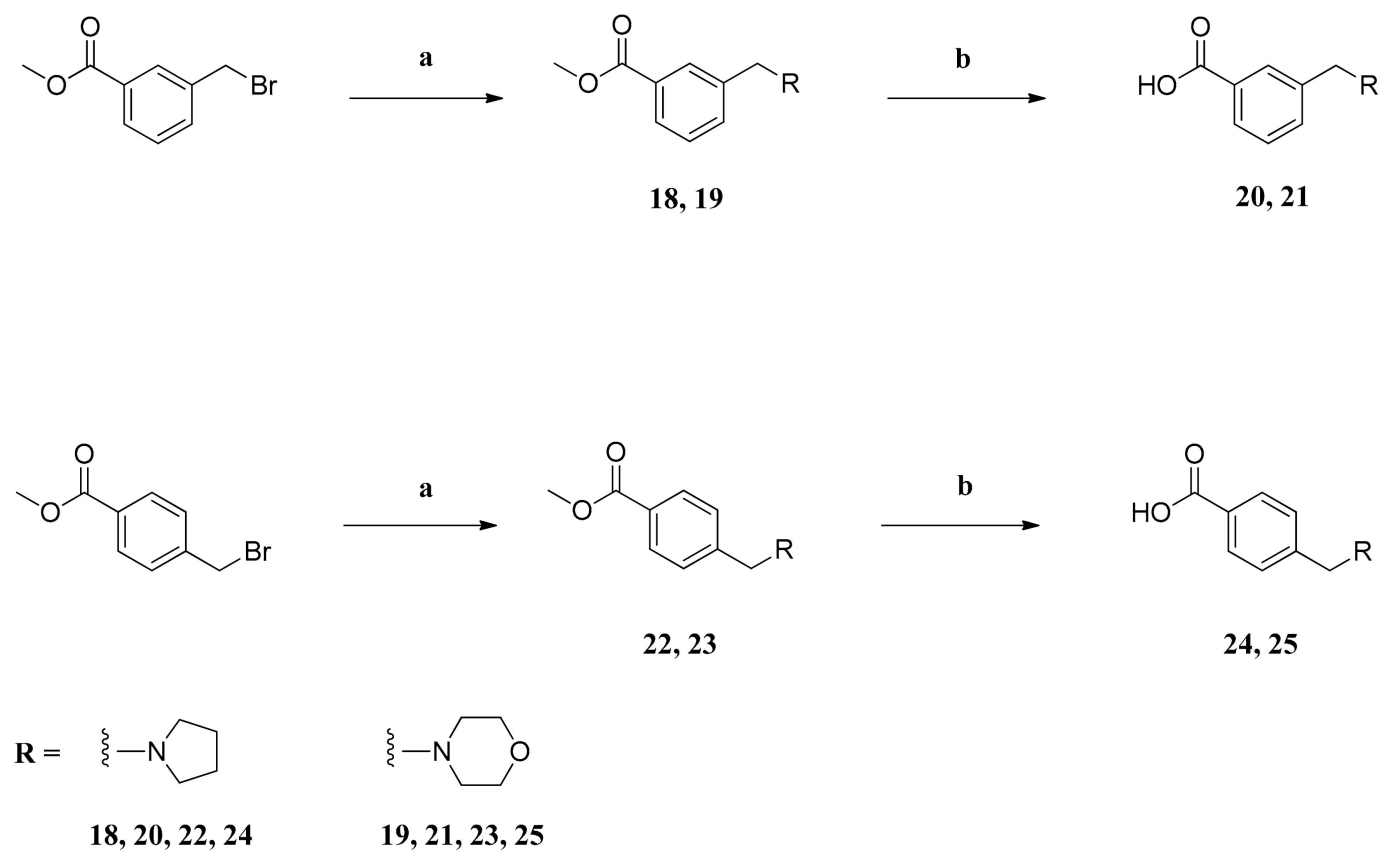
Preparation of compounds **20**, **21**, **24** and **25**. Reagents and conditions: (**a**) amine, NaI, K_2_CO_3_, acetonitrile, 80 °C, 20 h; (**b**) 2*N* NaOH, MeOH, rt, 20 h.

The carboxylic acids used for the synthesis of compounds **26**–**29** had to be synthesized from methyl 3-(bromomethyl)benzoate and methyl 4-(bromomethyl)benzoate (Scheme 3). The benzyl bromides reacted with the respective heterocyclic amines, potassium carbonate and catalytic amounts of sodium iodide in acetonitrile to form the tertiary amines **18**, **19**, **22** and **23**. Treatment of the methyl esters with 2*N* NaOH in methanol gave the desired carboxylic acids **20**, **21**, **24** and **25** [31]. 

Within another series of derivatives, the planar aromatic system was replaced by different aliphatic heterocycles. Furthermore, we also modified the chain length between the amide carbonyl groups and the ω-(dialkylamino) groups. Aside from a methylene linker, the influence of an ethyl linker was investigated.

**Scheme 4:**
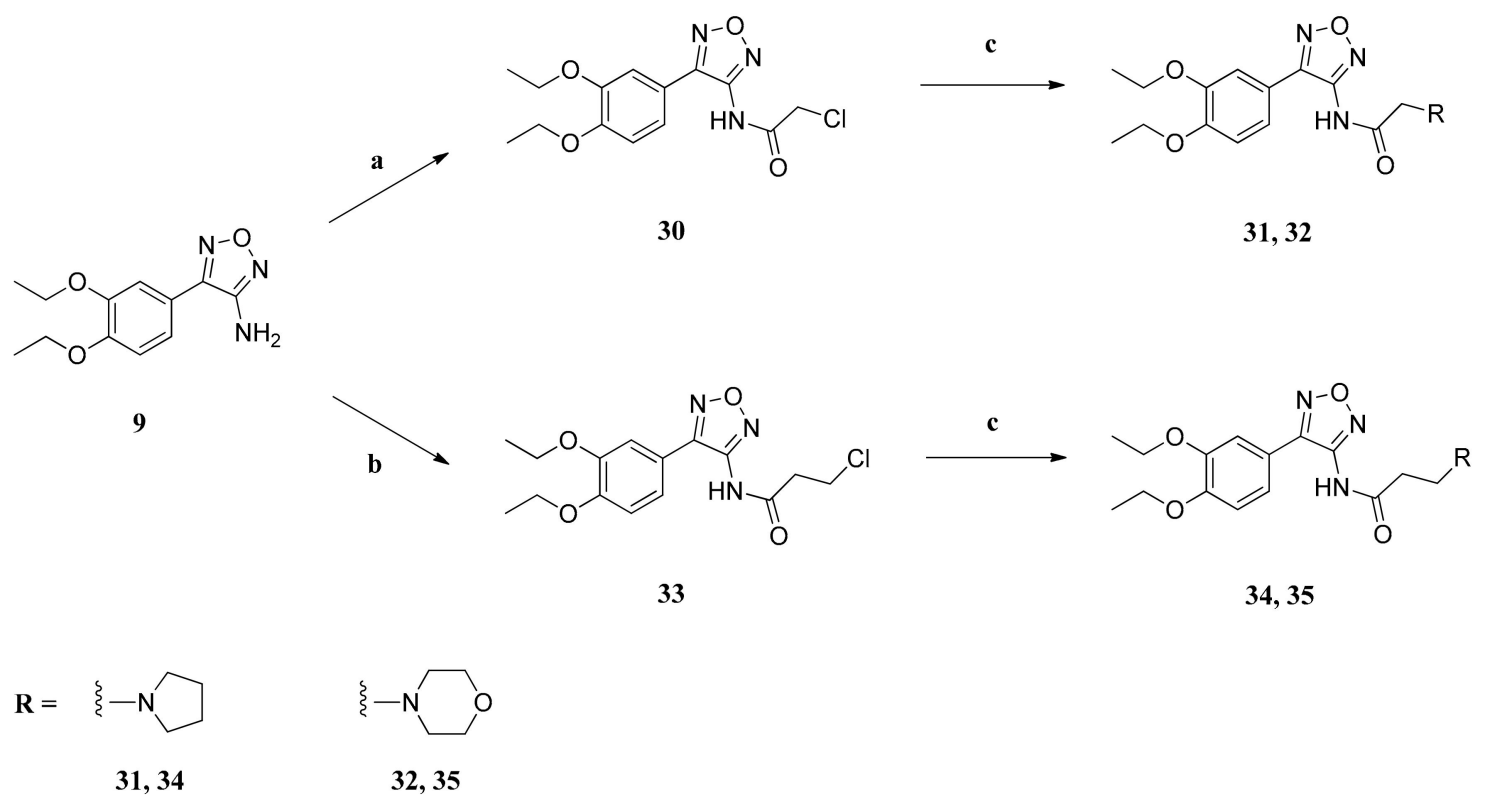
Preparation of compounds **31**, **32**, **34** and **35**. Reagents and conditions: (**a**) chloroacetyl chloride, pyridine, diethyl ether, benzene, 80 °C, 20 h; (**b**) 3-chloropropanoyl chloride, pyridine, diethyl ether, benzene, 80 °C, 20 h; (**c**) amine, NaI, K_2_CO_3_, acetonitrile, 80 °C, 20 h.

To obtain compounds **31**, **32**, **34** and **35**, the amino furazan **9** was treated at first with the corresponding ω-chloroacyl chloride, yielding the ω-chloroalkanamides **30** and **33** [32]. These were treated with the corresponding amines, giving the pyrrolidine derivatives **31** and **34**, as well as the morpholine derivatives **32** and **35** (Scheme 4).

To gain further insight regarding SARs, we also synthesized compound **39**. This compound possesses a 4-phenyl substituent instead of a 4-(3,4-diethoxyphenyl) substituent when compared to compound **1**.

**Scheme 5:**
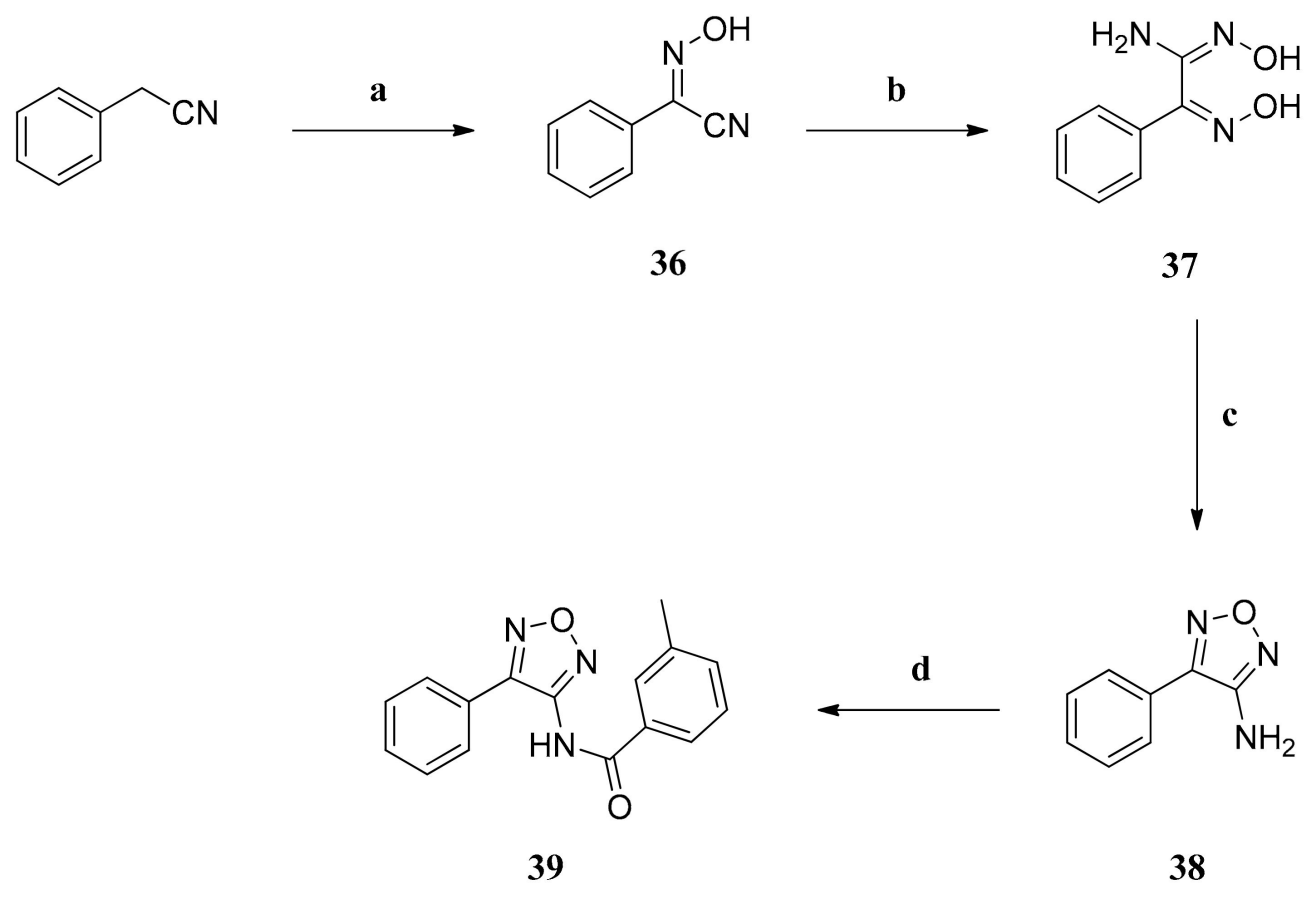
Preparation of compound **39**. Reagents and conditions: (**a**) (1) NaOEt, 0 °C, 30 min; (2) 3-methylbutyl nitrite, EtOH, rt, 20 h; (**b**) NH_2_OH x HCl, NaHCO_3_, MeOH, H_2_O, 100 °C, 20 h; (**c**) 2*N* NaOH, 100 °C, 20 h; (**d**) (1) NaH, DMF, 0 °C, 20 min; (2) 3-methylbenzoyl chloride, DMF, 60 °C, 48 h.

The synthesis was similar to the synthesis of compound **1** (Scheme 1). Starting from benzyl cyanide, the oxime **36** was obtained by treatment of the nitrile with 3-methylbutyl nitrite after deprotonation with sodium ethylate. The cyano group was further converted to the amidoxime **37** and further on to the aminofurazan **38** after a cyclization reaction. The final compound **39** was obtained by an amide reaction of the aminofurazan **38** with 3-methylbenzoyl chloride (Scheme 5).

### 2.2. Antiplasmodial Activity and Cytotoxicity

All newly synthesized compounds were at first tested in vitro for their antiplasmodial activity against the chloroquine-sensitive strain NF54 of *P. falciparum*. The most active compounds were further tested against the K_1_ strain of *P. falciparum*, which is resistant to chloroquine and pyrimethamine. Cytotoxicity was determined using rat skeletal myoblasts (L-6 cells). As standards served chloroquine, artemisinin and podophyllotoxin.

In order to evaluate the influence of a series of inserted acyl moieties, the 4-(3,4-diethoxyphenyl) substituent of the 3-aminofurazan was left unaltered. The substitution pattern of the aromatic system was diversified to evaluate the importance of the *meta*-methyl group of furazan **1**. The methyl group was replaced by several other substituents, including (dialkylamino)methyl substitution in *meta*- and *para*-position. Furthermore, the benzoyl moiety was exchanged with ω-(dialkylamino)acyl residues in order to observe the importance of the aromatic system for activity. The variation of the linker length between the amide and the aliphatic heterocyclic amines might increase the flexibility of the substituents, resulting in a positive effect on cytotoxicity.

Compound **1** served as comparison for all newly synthesized compounds (Table 1). It shows no difference in activity against the chloroquine-sensitive strain NF54 (*Pf*NF54 IC_50_ = 0.011 µM) and the multiresistant K_1_ strain (*Pf*K_1_ IC_50_ = 0.011 µM) of *Plasmodium falciparum*. The low cytotoxicity (L-6 cells IC_50_ = 159.3 µM) combined with the high antiplasmodial activity results in an excellent selectivity index for both strains (SI*_Pf_*_NF54_ = SI*_Pf_*_K1_ = 14,483). Compound **10** is the 3-demethyl analog of **1**. It shows still high antiplasmodial activity against *Pf*NF54 (IC_50_ = 0.076 µM) and *Pf*K_1_ (IC_50_ = 0.091 µM) but a 7–8-fold decrease in activity when compared to **1**. The 2-substituted compounds **11** and **12** (*Pf*NF54 IC_50_ = 0.343–0.831 µM) and the 4-substituted compounds **26** and **27** (*Pf*NF54 IC_50_ = 0.674–4.055 µM) possess only moderate or very weak antiplasmodial activity. With the exception of the only moderately active 3-(morpholin-4-yl)methyl derivative **29** (*Pf*NF54 IC_50_ = 0.712 µM), the series of 3-substituted analogs exhibited good to excellent activity (*Pf*NF54 IC_50_ = 0.014–0.192 µM). Its 3-(pyrrolidin-4-yl)methyl analog **28** showed a 4-fold increase in activity (*Pf*NF54 IC_50_ = 0.192 µM). The fluorinated analogs **13**, **14** and **15** were among the most active of the new compounds (*Pf*NF54 IC_50_ = 0.019–0.098 µM). Compared to its activity against *Pf*NF54 (IC_50_ = 0.049 µM), the 3-fluoro derivative **14** showed the expected decrease in activity against the multiresistant strain *Pf*K_1_ (IC_50_ = 0.108 µM). However, its 3-(trifluoromethyl) analog **13** was even more active against the multiresistant strain *Pf*K_1_ (IC_50_ = 0.007 µM) than against *Pf*NF54 (IC_50_ = 0.019 µM). Against this strain, it even surpassed the activity of compound **1** (*Pf*K_1_ IC_50_ = 0.011 µM) and showed similar activity to artemisinin (*Pf*K_1_ IC_50_ = 0.0064 µM). Likewise, the 3-substituted 4-methoxy compounds **16** and **17** exhibited promising activity against *Pf*NF54 (IC_50_ = 0.014–0.167 µM). Against the sensitive strain, the 3-chloro-4-methoxybenzamide **16** possessed the highest activity (*Pf*NF54 IC_50_ = 0.014 µM), which is similar to **1** (*Pf*NF54 IC_50_ = 0.011 µM). Replacement of the benzamide by a ω-(dialkylamino)acetamide or a ω-(dialkylamino)propanamide moiety led to inactive compounds **31**, **32**, **34** and **35** (*Pf*NF54 IC_50_ = 7.24–56.01 µM). In comparison to **1** (L-6 cells IC_50_ = 159.3 µM), all of the more active compounds showed increased cytotoxicity (L-6 cells IC_50_ = 9.24–111.2 µM). However, the selectivity indexes of the most promising compounds **10**, **13**, **14** and **16** were still very high (S.I. = 380–1463).

The removal of both ethoxy groups of the left-hand side ring, like in compound **39**, resulted in a massive loss in activity against the chloroquine-sensitive strain *Pf*NF54 (IC_50_ = 21.52 µM) and the multiresistant strain *Pf*K_1_ (IC_50_ = 39.92 µM). This affirms the positive impact of the 3,4-diethoxyphenyl substitution pattern.

### 2.3. Physicochemical Properties and Permeability

In addition to antiplasmodial activity tests of compounds **1**, **10**–**17**, **26**–**29**, **31**, **32**, **34**, **35** and **39**, physicochemical parameters like log P and log D_7.4_ were calculated (using the ChemAxon software JChem for Excel). Furthermore, test compounds had to meet conditions of sufficient ligand efficiency (LE) [33] and effective permeability (P_e_). The latter was determined by a PAMPA (Table 2) [34]. The log P values of the compounds range between 1.50 and 5.11. The inactive ω-(dialkylamino)alkanamides **31**, **32**, **34** and **35** have by far the lowest log P values (log P = 1.50–2.36). The most active compounds have good log *p* values (log P = 3.66–4.56). All compounds with considerable antiplasmodial activity have log D_7.4_ values ranging from 3.09 to 5.11. The permeability of compounds was only detectable for selected compounds due to insufficient solubility or excessive mass retention in the PAMPA. All new compounds showed increased permeability (P_e_ = 3.90–10.80 × 10^−6^ cm/s) in comparison to **1** (P_e_ = 2.77 × 10^−6^ cm/s). The most promising compound **13** has only slightly higher permeability than **1** (P_e_ = 4.26 × 10^−6^ cm/s), whereas the inactive ω-aminoacetamides **31** and **32** show by far the best permeabilities (P_e_ = 10.25–10.80 × 10^−6^ cm/s). However, in general, substances with a permeability above 1.5 × 10^−6^ cm/s are considered as having good permeability.

Ligand efficiency has become an important concept in drug development, partly due to the realization that large ligands have a disadvantage in terms of the molecular properties necessary for bioavailability. It is defined as the binding free energy for a ligand divided by its number of heavy atoms (HA). Proposed acceptable values for drug candidates are ~0.3 kcal/mol/HA and higher. Out of all tested compounds, **1** has the highest ligand efficiency (LE = 0.404 kcal/mol/HA). The compounds **10**, **13**, **14** and **16** also exhibit promising ligand efficiencies (LE = 0.353–0.375 kcal/mol/HA).

## 3. Materials and Methods

### 3.1. Instrumentation and Chemicals

Melting points were obtained on an Electrothermal IA 9200 melting point apparatus. IR spectra: Bruker Alpha Platinum ATR FTIR spectrometer (KBr discs); frequencies are reported in cm^−1^. The structures of all newly synthesized compounds were determined by one- and two-dimensional NMR spectroscopy. NMR spectra: Varian UnityInova 400 (298 K) 5 mm tubes, TMS as internal standard. Shifts in ^1^H NMR (400 MHz) and ^13^C NMR (100 MHz) spectra are reported in ppm; ^1^H- and ^13^C-resonances were assigned using ^1^H,^1^H- and ^1^H,^13^C-correlation spectra and are numbered as given in Scheme 1. Signal multiplicities are abbreviated as follows: br, broad; d, doublet; dd, doublet of doublets; ddd, doublet of doublet of doublets; m, multiplet; s, singlet; t, triplet; td, triplet of doublets; q, quartet. HRMS: Micromass Tofspec 3E spectrometer (MALDI) and GCT-Premier, Waters (EI, 70 eV). Materials: column chromatography (CC): silica gel 60 (Merck 70–230 mesh, pore diameter 60 Å), aluminium oxide (pH: 9.5, Fluka), thin-layer chromatography (TLC): TLC plates silica gel 60 F254 (Merck), aluminium oxide 60 F254 (neutral, Merck). Experiments to assess the purity of final compounds were carried out on an Agilent 1110 HPLC device (Agilent Technologies, Palo Alto, CA, USA) equipped with an auto sampler and a VWL detector. Ultraviolet detection was performed at 214 nm. The measurements were carried out under isocratic conditions at ambient temperature with a flowrate of 2 mL/min and an injection volume of 20 µL. Data were collected with Chemstation Rev. B. 0903 (Agilent Technologies, Waldbronn, Germany) software. A LiChrospher 100 RP-18e, 125 mm × 4 mm, 3 µm from Merck KGaA (Darmstadt, Germany) served as stationary phase. Mobile phase was prepared by mixing a 20 mM ammonium phosphate buffer adjusted to pH 2.4 and acetonitrile (2:1). Samples were prepared by dissolving 1 mg each in 1 mL methanol. Unless otherwise noted, all compounds were found to be >95% pure by this method. PAMPA: 96-well precoated Corning Gentest PAMPA plate (Corning, Glendale, AZ, USA), 96-well UV-Star Microplates (Greiner Bio-One, Kremsmünster, Austria), SpectraMax M3 UV plate reader (Molecular Devices, San Jose, CA, USA). ^1^H-NMR and ^13^C-NMR spectra of new compounds are available in Supplementary Materials Section (Figures S1–S23).

### 3.2. Syntheses

*(3,4-Diethoxyphenyl)methanol* (**5**): NaBH_4_ (0.57 g (15.00 mmol)) was added in portions to an ice-cooled solution of 3,4-diethoxybenzaldehyde (2.91 g (15.00 mmol)) in dry methanol (16 mL). After that, the ice bath was removed and the reaction mixture was stirred at 25 °C for 1 h. Then, the solvent was evaporated in vacuo and the residue was mixed with water and extracted with CH_2_Cl_2_. The combined organic phases were washed with water, dried over anhydrous sodium sulfate and filtered. The solvent was evaporated in vacuo giving compound **5** as colorless oil (2.80 g (95%)), which was used without further purification.

NMR data were in accordance with literature data [35].

*4-(Chloromethyl)-1,2-diethoxybenzene* (**6**): Thionyl chloride (4.14 g (34.80 mmol)) was added dropwise via a dropping funnel to an ice-cooled solution of benzyl alcohol **5** (2.36 g (12.00 mmol)) in dry CH_2_Cl_2_ (50 mL). The ice bath was removed and the reaction mixture was stirred at 25 °C for 20 h. Then, the reaction was quenched with 2*N* NaOH at 0 °C and the mixture was basified to a pH of 10–11. The aqueous and organic phases were separated and the aqueous phase was extracted with CH_2_Cl_2_. The combined organic phases were dried over anhydrous sodium sulfate and filtered and the solvent was removed in vacuo, giving compound **6** as brown oil (2.47 g (96%)), which was used without further purification. IR = 2977, 1604, 1510, 1476, 1428, 1392, 1256, 1232, 1135, 1038, 985, 805; ^1^H NMR (CDCl_3_, 400 MHz) *δ* = 1.42–1.47 (m, 6H, 2 CH_3_), 4.06–4.13 (m, 4H, 2 CH_2_), 4.55 (s, 2H,CH_2_Cl), 6.82 (d, *J* = 8.1 Hz, 1H, 5-H), 6.90 (dd, *J* = 8.1, 2.0 Hz, 1H, 6-H), 6.91 (d, *J* = 2.0 Hz, 1H, 2-H); ^13^C NMR (CDCl_3_, 100 MHz) *δ* = 14.76 (2 CH_3_), 46.71 (CH_2_Cl), 64.56 (2 CH_2_), 113.08 (C-5), 113.86 (C-2), 121.21 (C-6), 129.96 (C-1), 148.81 (C-3), 148.93 (C-4); HRMS (EI+) calcd for C_11_H_15_ClO_2_: 214.0761; found: 214.0755.

*2-(3,4-Diethoxyphenyl)acetonitrile* (**7**): Benzyl chloride **6** (2.15 g (10.00 mmol)) was dissolved in dry DMF (20 mL). KCN (1.30 g (20.00 mmol)) was added and the suspension was stirred at 100 °C for 4 h. After that, the solvent was evaporated in vacuo and the residue was mixed with water and extracted with ethyl acetate. The organic phases were combined and washed with water and brine, dried over anhydrous sodium sulfate and filtered. The solvent was evaporated in vacuo, giving compound **7** as brown oil (1.95 g (95%)), which was used without further purification.

NMR data were in accordance with literature data [36].

*(3,4-Diethoxyphenyl)(hydroxyimino)acetonitrile* (**2**): Sodium (0.37 g (12.00 mmol)) was dissolved in dry ethanol (15 mL) and then cooled to 0 °C with an ice bath. A solution of compound **7** (1.64 g (8.00 mmol)) in dry ethanol (10 mL) was added dropwise. Finally, isopentyl nitrite (1.41 g (12.00 mmol)) was added dropwise with a syringe through a septum. The ice bath was removed and the reaction mixture stirred at 25 °C for 20 h. The solution was diluted with ethyl acetate (80 mL) and washed with 2*N* HCl, 8% aq NaHCO_3_ and brine. The organic phase was dried over anhydrous sodium sulfate and filtered and the solvent was removed in vacuo, giving compound **2** as white amorphous solid (1.86 g (99%)), which was used without further purification. IR = 3364, 1601, 1512, 1438, 1337, 1273, 1210, 1146, 1076, 1006, 845, 806; ^1^H NMR (CDCl_3_, 400 MHz) *δ* = 1.42 (t, *J* = 6.9 Hz, 3H, CH_3_), 1.43 (t, *J* = 6.9 Hz, 3H, CH_3_), 4.08 (q, *J* = 6.9 Hz, 2H, OCH_2_), 4.10 (q, *J* = 6.9 Hz, 2H, OCH_2_), 7.00 (d, *J* = 8.5 Hz, 1H, 5-H), 7.26 (dd, *J* = 8.5, 1.9 Hz, 1H, 6-H), 7.33 (d, *J* = 1.9 Hz, 1H, 2-H); ^13^C NMR (CDCl_3_, 100 MHz) *δ* = 15.16 (CH_3_), 15.19 (CH_3_), 65.75 (OCH_2_), 65.89 (OCH_2_), 110.68 (C-2), 111.04 (CN), 114.03 (C-5), 121.22 (C-6), 124.25 (C-1), 132.87 (C=NOH), 150.49 (C-3), 152.73 (C-4); HRMS (EI+) calcd for C_12_H_14_N_2_O_3_: 234.1004; found: 234.0996.

*2-(3,4-Diethoxyphenyl)-N’-hydroxy-2-(hydroxyimino)ethanimidamide* (**8**): Oxime **2** (1.41 g (6.00 mmol)) was dissolved in methanol (18 mL) and a solution of NH_2_OH × HCl (0.50 g (7.20 mmol)) and NaHCO_3_ (0.61 g (7.20)) in water (9 mL) was added. The mixture was refluxed for 20 h and then the solvent was evaporated in vacuo. The residue was mixed with water (20 mL) and extracted with ethyl acetate. The organic phases were combined dried over anhydrous sodium sulfate and filtered. The solvent was evaporated in vacuo, giving the raw amidoxime which was recrystallized in CH_2_Cl_2_ affording compound **8** as beige crystals (0.55 g (34%)). m.p. 148 °C. IR = 3370, 2981, 1661, 1598, 1516, 1444, 1393, 1270, 1201, 1144, 1040, 978, 810; ^1^H NMR (DMSO-d_6_, 400 MHz) *δ* = 1.33 (t, *J* = 7.0 Hz, 6H, 2 CH_3_), 3.97–4.07 (m, 4H, 2 OCH_2_), 5.67 (s, 2H, NH_2_), 6.95 (d, *J* = 8.4 Hz, 1H, 5-H), 7.06 (dd, *J* = 8.4, 1.9 Hz, 1H, 6-H), 7.22 (d, *J* = 1.9 Hz, 1H, 2-H), 9.39 (s, 1H, NOH), 11.34 (s, 1H, NOH); ^13^C NMR (DMSO-d_6_, 100 MHz) *δ* = 14.66 (CH_3_), 14.72 (CH_3_), 63.75 (OCH_2_), 63.81 (OCH_2_), 110.59 (C-2), 112.59 (C-5), 120.24 (C-6), 127.24 (C-1), 146.64 (C(=NOH)NH_2_), 147.66 (C-3), 149.20 (C-4), 149.22 (C=NOH); HRMS (EI+) calcd for C_12_H_17_N_3_O_4_: 267.1219; found: 267.1230.

*4-(3,4-Diethoxyphenyl)-1,2,5-oxadiazol-3-amine* (**9**): Amidoxime **8** (0.80 g (3.00 mmol)) was dissolved in 2*N* NaOH (30 mL) and refluxed for 20 h. The formed precipitate was filtered, washed with water and dried. The beige precipitate was pure product **9** (0.67 g (83%)) and was used without further purification. m.p. 155 °C. IR = 3374, 3322, 3244, 2984, 1593, 1536, 1501, 1396, 1327, 1305, 1253, 1215, 1141, 1111, 1041, 941, 862, 848, 815; ^1^H NMR (DMSO-d_6_, 400 MHz) *δ* = 1.35 (t, J = 6.9 Hz, 6H, 2 CH_3_), 4.10 (q, *J* = 6.9 Hz, 4H, 2 OCH_2_), 6.12 (s, 2H, NH_2_), 7.09 (d, *J* = 8.4 Hz, 1H, 5’-H), 7.25 (d, *J* = 1.8 Hz, 1H, 2’-H), 7.29 (dd, *J* = 8.4, 1.8 Hz, 1H, 6’-H); ^13^C NMR (DMSO-d_6_, 100 MHz) *δ* = 14.62 (CH_3_), 14.66 (CH_3_), 63.85 (2 OCH_2_), 112.20 (C-2’), 113.25 (C-5’), 117.65 (C-1’), 120.60 (C-6’), 146.69 (C-4), 148.35 (C-3’), 149.87 (C-4’), 155.21 (C-3); HRMS (EI+) calcd for C_12_H_15_N_3_O_3_: 249.1113; found: 249.1099.

The general procedure for the synthesis of tertiary amines (**18**, **19**, **22** and **23**) is as follows: The respective heterocyclic amine (8.00 mmol), K_2_CO_3_ (8.00 mmol) and a catalytical amount of NaI (3.20 mmol) were added to a solution of the corresponding benzyl bromide (4.00 mmol) in dry acetonitrile (40 mL). The mixture was refluxed for 20 h. The suspension was cooled and filtered. The filtrate was evaporated in vacuo to dryness. The residue was mixed with water and the aqueous phase was extracted with CH_2_Cl_2_. The combined organic phases were washed with 8% aq NaHCO_3_ and brine. The organic phase was dried over anhydrous sodium sulfate and filtered and the solvent was removed in vacuo, giving pure amines **18**, **19**, **22** and **23**, which were used without further purification.

*Methyl 3-((pyrrolidin-1-yl)methyl)benzoate* (**18**): The reaction of methyl 3-(bromomethyl)benzoate (0.92 g (4.00 mmol)), pyrrolidine (0.57 g (8.00 mmol)), K_2_CO_3_ (1.11 g (8.00 mmol)) and NaI (0.48 g (3.20 mmol)) in dry acetonitrile (40 mL) gave compound **18** as pale brown oil (0.87 g (99%)).

NMR data were in accordance with literature data [37].

*Methyl 3-((morpholin-4-yl)methyl)benzoate* (**19**): The reaction of methyl 3-(bromomethyl)benzoate (0.92 g (4.00 mmol)), morpholine (0.70 g (8.00 mmol)), K_2_CO_3_ (1.11 g (8.00 mmol)) and NaI (0.48 g (3.20 mmol)) in dry acetonitrile (40 mL) gave compound **19** as yellow oil (0.93 g (99%)).

NMR data were in accordance with literature data [38].

*Methyl 4-((pyrrolidin-1-yl)methyl)benzoate* (**22**): The reaction of methyl 4-(bromomethyl)benzoate (0.92 g (4.00 mmol)), pyrrolidine (0.57 g (8.00 mmol)), K_2_CO_3_ (1.11 g (8.00 mmol)) and NaI (0.48 g (3.20 mmol)) in dry acetonitrile (40 mL) gave compound **22** as pale brown oil (0.82 g (94%)).

NMR data were in accordance with literature data [39].

*Methyl 4-((morpholin-4-yl)methyl)benzoate* (**23**): The reaction of methyl 4-(bromomethyl)benzoate (0.92 g (4.00 mmol)), morpholine (0.70 g (8.00 mmol)), K_2_CO_3_ (1.11 g (8.00 mmol)) and NaI (0.48 g (3.20 mmol)) in dry acetonitrile (40 mL) gave compound **23** as yellow oil (0.90 g (96%)).

NMR data were in accordance with literature data [40].

*2-(Hydroxyimino)-2-phenylacetonitrile* (**36**): Sodium (0.37 g (12.00 mmol)) was dissolved in dry ethanol (15 mL) and then cooled to 0 °C with an ice bath. A solution of benzyl cyanide (0.94 g (8.00 mmol)) in dry ethanol (10 mL) was added dropwise. Finally, isopentyl nitrite (1.41 g (12.00 mmol)) was added dropwise with a syringe through a septum. The ice bath was removed and the reaction mixture stirred at 25 °C for 20 h. The solution was diluted with ethyl acetate (80 mL) and washed with 2*N* HCl, 8% aq NaHCO_3_ and brine. The organic phase was dried over anhydrous sodium sulfate and filtered and the solvent was removed in vacuo, giving compound **36** as yellow amorphous solid (1.15 g (98%)), which was used without further purification.

NMR data were in accordance with literature data [41].

*N’-Hydroxy-2-(hydroxyimino)-2-phenylethanimidamide* (**37**): Oxime **36** (0.88 g (6.00 mmol)) was dissolved in methanol (18 mL) and a solution of NH_2_OH × HCl (0.50 g (7.20 mmol)) and NaHCO_3_ (0.61 g (7.20)) in water (9 mL) was added. The mixture was refluxed for 20 h and then the solvent was evaporated in vacuo. The residue was mixed with water (20 mL) and extracted with ethyl acetate. The organic phases were combined, dried over anhydrous sodium sulfate and filtered. The solvent was evaporated in vacuo, giving the raw amidoxime which was recrystallized in CH_2_Cl_2_ affording compound **37** as white crystals (0.25 g (23%)).

NMR data were in accordance with literature data [42].

*4-Phenyl-1,2,5-oxadiazol-3-amine* (**38**): Amidoxime **37** (0.54 g (3.00 mmol)) was dissolved in 2*N* NaOH (30 mL) and refluxed for 20 h. The formed precipitate was filtered, washed with water and dried. The white precipitate was pure product **38** (0.17 g (36%)) and was used without further purification.

NMR data were in accordance with literature data [43].

The general procedure for the synthesis of carboxylic acids (**20**, **21**, **24** and **25**) is as follows: To a solution of the respective methyl ester (3.00 mmol) in methanol (10 mL), 2*N* NaOH (9.0 mL) was added. The mixture was stirred at 25 °C for 20 h and then the reaction mixture was acidified with conc HCl to a pH of 6. The solvent was evaporated in vacuo and the residue was mixed with CH_2_Cl_2_ (20 mL) and sonicated for 5 min. The suspension was filtered and the filtrate was evaporated in vacuo to dryness, giving pure carboxylic acids **20, 21, 24** and **25**, which were used without further purification.

*3-((Pyrrolidin-1-yl)methyl)benzoic acid* (**20**): The reaction of methyl ester **18** (0.66 g (3.00 mmol)) and 2*N* NaOH (9 mL) in methanol (10 mL) gave compound **20** as white foam (0.17 g (27%)). IR = 2927, 2597, 1613, 1567, 1455, 1381, 1263; ^1^H NMR (CDCl_3_, 400 MHz) *δ* = 2.12 (br, s, 4H, (CH_2_)_2_), 3.26 (br, 4H, N(CH_2_)_2_), 4.20 (br, s, 2H, ArCH_2_), 7.40 (t, *J* = 7.7 Hz, 1H, 5-H), 7.55 (br, 1H, 4-H), 8.07 (d, *J* = 7.7 Hz, 1H, 6-H), 8.50 (br, s, 1H, 2-H); ^13^C NMR (CDCl_3_, 100 MHz) *δ* = 23.19 ((CH_2_)_2_), 52.71 (N(CH_2_)_2_), 58.49 (ArCH_2_), 128.33 (C-5), 130.39 (C-6), 130.75 (C-1), 132.47 (C-2), 132.55 (C-4), 136.70 (C-3), 170.88 (COOH); HRMS (EI+) calcd for C_12_H_15_NO_2_: 205.1103; found: 205.1088.

*3-((Morpholin-4-yl)methyl)benzoic acid* (**21**): The reaction of methyl ester **19** (0.71 g (3.00 mmol)) and 2*N* NaOH (9 mL) in methanol (10 mL) gave compound **21** as white amorphous solid (0.64 g (97%)).

NMR data were in accordance with literature data [44].

*4-((Pyrrolidin-1-yl)methyl)benzoic acid* (**24**): The reaction of methyl ester **22** (0.66 g (3.00 mmol)) and 2*N* NaOH (9 mL) in methanol (10 mL) gave compound **24** as white foam (0.55 g (90%)).

NMR data were in accordance with literature data [39].

*4-((Morpholin-4-yl)methyl)benzoic acid* (**25**): The reaction of methyl ester **23** (0.71 g (3.00 mmol)) and 2*N* NaOH (9 mL) in methanol (10 mL) gave compound **25** as yellow oil (0.62 g (94%)).

NMR data were in accordance with literature data [40].

The general procedure for the synthesis of carboxamides (**1**, **10**–**17**, **26**–**29**) is detailed in the following subsections.

#### 3.2.1. Method A

An ice-cooled suspension of NaH (60% dispersion in mineral oil; 2.00 mmol) in dry DMF (14 mL) was mixed with aminofurazan **9** (1.00 mmol) and stirred for 20 min. Then, a solution of acid chloride (1.30 mmol) in dry DMF (2 mL) was added dropwise and the reaction mixture was stirred at 60 °C for 20 h. Afterward, the mixture was quenched with water at 0 °C and the aqueous phase was extracted with CH_2_Cl_2_. The organic layer was washed with 8% aq NaHCO_3_ and brine, dried over anhydrous sodium sulfate and filtered, and the solvent was evaporated in vacuo, yielding the raw carboxamides **1** and **10**–**14**, which were further purified by crystallization.

#### 3.2.2. Method B

To an ice-cooled solution of carboxylic acid (1.50 mmol) in dry CH_2_Cl_2_ (14 mL), oxalyl dichloride 2 M in CH_2_Cl_2_ (1.90 mmol) was added dropwise under stirring. After 1 h, the ice bath was removed and the reaction batch was stirred 20 h at 25 °C in an atmosphere of Ar. Subsequently, the solvent was evaporated in vacuo and the crude acyl chloride was dissolved in dry DMF (9 mL). An ice-cooled suspension of NaH (60% dispersion in mineral oil; 2.00 mmol) in dry DMF (14 mL) was mixed with aminofurazan **9** (1.00 mmol) and stirred for 20 min. Then, the solution of acyl chloride in dry DMF was added dropwise and the reaction mixture was stirred at 60 °C for 48 h. Afterward, the mixture was quenched with water at 0 °C and the aqueous phase was extracted with CH_2_Cl_2_. The organic layer was washed with 8% aq NaHCO_3_ and brine, dried over anhydrous sodium sulfate and filtered, and the solvent was evaporated in vacuo, yielding the raw carboxamides **16**, **17** and **26**–**29**, which were further purified by column chromatography or crystallization.

#### 3.2.3. Method C

A solution of benzoic acid (1.50 mmol), *N*-hydroxysuccinimide (1.58 mmol) and *N*,*N*′-dicyclohexylcarbodiimide (1.50 mmol) was dissolved in dry THF (10 mL) and stirred at 25 °C for 20 h. The formed precipitate was filtered and the filtrate was evaporated in vacuo to dryness to obtain the crude NHS ester which was used without further purification. An ice-cooled suspension of NaH (60% dispersion in mineral oil; 2.00 mmol) in dry DMF (14 mL) was mixed with aminofurazan **9** (1.00 mmol) and stirred for 20 min. Then, the solution of NHS ester in dry DMF (4 mL) was added dropwise and the reaction mixture was stirred at 60 °C for 20 h. Afterward, the mixture was quenched with water at 0 °C and the aqueous phase was extracted with CH_2_Cl_2_. The organic layer was washed with 8% aq NaHCO_3_ and brine, dried over anhydrous sodium sulfate and filtered, and the solvent was evaporated in vacuo, yielding the raw carboxamide **15**, which was further purified by crystallization.

*N-(4-(3,4-Diethoxyphenyl)-1,2,5-oxadiazol-3-yl)-3-methylbenzamide* (**1**): Method A: The reaction of compound **9** (249 mg (1.00 mmol)), 3-methylbenzoyl chloride (201 mg (1.30 mmol)) and NaH (60% dispersion in mineral oil) (80 mg (2.00 mmol)) in dry DMF (16 mL) gave the raw carboxamide which was purified by recrystallization in CH_2_Cl_2_ to yield compound **1** as white crystals (63 mg (17%)). m.p. 162 °C. IR = 3198, 2981, 1665, 1591, 1531, 1500, 1394, 1377, 1325, 1277, 1260, 1218, 1142, 1042, 940, 862, 810; ^1^H NMR (DMSO-d_6_, 400 MHz) *δ* = 1.20 (t, *J* = 6.9 Hz, 3H, CH_3_), 1.32 (t, *J* = 6.9 Hz, 3H, CH_3_), 2.40 (s, 3H, ArCH_3_), 3.90 (q, *J* = 6.9 Hz, 2H, OCH_2_), 4.05 (q, *J* = 6.9 Hz, 2H, OCH_2_), 7.07 (d, *J* = 8.4 Hz, 1H, 5”-H), 7.32-7.37 (m, 2H, 2”-H, 6”-H), 7.45 (t, *J* = 7.6, 1H, 5-H), 7.49 (d, *J* = 7.6 Hz, 1H, 4-H), 7.79 (br, d, *J* = 7.4 Hz, 1H, 6-H), 7.82 (br, s, 1H, 2-H), 11.17 (br, 1H, NH); ^13^C NMR (DMSO-d_6_, 100 MHz) *δ* = 14.47 (CH_3_), 14.56 (CH_3_), 20.86 (ArCH_3_), 63.79 (2 OCH_2_), 111.68 (C-2”), 113.21 (C-5”), 116.92 (C-1”), 120.44 (C-6”), 125.17 (C-6), 128.52 (C-2), 128.61 (C-5), 132.22 (C-1), 133.41 (C-4), 138.13 (C-3), 148.10 (C-3”), 150.06 (C-3’), 150.33 (C-4”), 151.23 (C-4’), 166.78 (C=O); HRMS (EI+) calcd for C_20_H_21_N_3_O_4_: 367.1532; found: 367.1526.

*N-(4-(3,4-Diethoxyphenyl)-1,2,5-oxadiazol-3-yl)benzamide* (**10**): Method A: The reaction of compound **9** (249 mg (1.00 mmol)), benzoyl chloride (183 mg (1.30 mmol)) and NaH (60% dispersion in mineral oil) (80 mg (2.00 mmol)) in dry DMF (16 mL) gave the raw carboxamide which was purified by recrystallization in CH_2_Cl_2_ to yield compound **10** as white crystals (81 mg (23%)). m.p. 199 °C. IR = 3212, 2980, 1660, 1591, 1530, 1501, 1468, 1395, 1378, 1324, 1275, 1258, 1216, 1141, 1040, 916, 852, 811; ^1^H NMR (DMSO-d_6_, 400 MHz) *δ* = 1.19 (t, *J* = 6.9 Hz, 3H, CH_3_), 1.31 (t, *J* = 6.9 Hz, 3H, CH_3_), 3.88 (q, *J* = 7.0 Hz, 2H, OCH_2_), 4.05 (q, *J* = 6.9 Hz, 2H, OCH_2_), 7.07 (d, *J* = 8.4 Hz, 1H, 5”-H), 7.31 (d, *J* = 1.7 Hz, 1H, 2”-H), 7.33 (dd, *J* = 8.4, 1.7 Hz, 1H, 6”-H), 7.58 (t, *J* = 7.4 Hz, 2H, 3-H, 5-H), 7.68 (t, *J* = 7.4 Hz, 1H, 4-H), 8.00 (d, *J* = 7.2 Hz, 2H, 2-H, 6-H), 11.24 (br s, 1H, NH); ^13^C NMR (DMSO-d_6_, 100 MHz) *δ* = 14.45 (CH_3_), 14.57 (CH_3_), 63.78 (2 OCH_2_), 111.65 (C-2”), 113.19 (C-5”), 116.87 (C-1”), 120.43 (C-6”), 128.05 (C-2, C-6), 128.74 (C-3, C-5), 132.10 (C-1), 132.91 (C-4), 148.11 (C-3”), 149.95 (C-3’), 150.36 (C-4”), 151.24 (C-4’), 166.66 (C=O); HRMS (EI+) calcd for C_19_H_19_N_3_O_4_: 353.1375; found: 353.1373.

*N-(4-(3,4-Diethoxyphenyl)-1,2,5-oxadiazol-3-yl)-2-methylbenzamide* (**11**): Method A: The reaction of compound **9** (249 mg (1.00 mmol)), 2-methylbenzoyl chloride (201 mg (1.30 mmol)) and NaH (60% dispersion in mineral oil) (80 mg (2.00 mmol)) in dry DMF (16 mL) gave the raw carboxamide which was purified by recrystallization in CH_2_Cl_2_ to yield compound **11** as white crystals (103 mg (28%)). m.p. 170 °C. IR = 3214, 2983, 1663, 1591, 1503, 1395, 1377, 1324, 1259, 1216, 1142, 1042, 918, 854, 803, 741, 687, 657, 618; ^1^H NMR (DMSO-d_6_, 400 MHz) *δ* = 1.28 (t, *J* = 7.0 Hz, 3H, CH_3_), 1.34 (t, *J* = 7.0 Hz, 3H, CH_3_), 2.34 (s, 3H, ArCH_3_), 3.99 (q, *J* = 7.0 Hz, 2H, OCH_2_), 4.08 (q, *J* = 7.0 Hz, 2H, OCH_2_), 7.11 (d, *J* = 8.4 Hz, 1H, 5”-H), 7.33–7.37 (m, 4H, 2”-H, 3-H, 5-H, 6”-H), 7.45 (t, *J* = 7.3 Hz, 1H, 4-H), 7.65 (d, *J* = 7.3 Hz, 1H, 6H), 11.12 (br, 1H, NH); ^13^C NMR (DMSO-d_6_, 100 MHz) *δ* = 14.61 (2 CH_3_), 19.56 (ArCH_3_), 63.84 (OCH_2_), 63.97 (OCH_2_), 112.06 (C-2”), 113.13 (C-5”), 116.96 (C-1”), 120.67 (C-6”), 125.79 (C-5), 127.89 (C-6), 130.92 (C-4), 131.10 (C-3), 133.97 (C-1), 136.63 (C-2), 148.21 (C-3”), 149.83 (C-3´), 150.34 (C-4”), 151.22 (C-4´), 168.70 (C=O); HRMS (EI+) calcd for C_19_H_21_N_3_O_4_: 367.1532; found: 367.1526.

*N-(4-(3,4-Diethoxyphenyl)-1,2,5-oxadiazol-3-yl)-2-(trifluoromethyl)benzamide* (**12**): Method A: The reaction of compound **9** (249 mg (1.00 mmol)), 2-(trifluoromethyl)benzoyl chloride (271 mg (1.30 mmol)) and NaH (60% dispersion in mineral oil) (80 mg (2.00 mmol)) in dry DMF (16 mL) gave the raw carboxamide which was purified by recrystallization in methanol to yield compound **12** as white crystals (38 mg (9%)). m.p. 171 °C. IR = 3209, 2983, 1676, 1604, 1531, 1494, 1397, 1377, 1316, 1277, 1217, 1177, 1129, 1043, 921, 854, 811, 772, 651, 617; ^1^H NMR (DMSO-d_6_, 400 MHz) *δ* = 1.32 (t, *J* = 7.0 Hz, 3H, CH_3_), 1.34 (t, *J* = 7.0 Hz, 3H, CH_3_), 4.05 (q, *J* = 7.0 Hz, 2H, OCH_2_), 4.09 (q, *J* = 7.0 Hz, 2H, OCH_2_), 7.13 (d, *J* = 8.2 Hz, 1H, 5”-H), 7.34 (d, *J* = 1.8 Hz, 1H, 2”-H), 7.36 (dd, *J* = 8.2, 1.8 Hz, 1H, 6”-H), 7.76-7.84 (m, 3H, 4-H, 5-H, 6-H), 7.89 (d, *J* = 8.2 Hz, 1H, 3-H), 11.46 (br, 1H, NH); ^13^C NMR (DMSO-d_6_, 100 MHz) *δ* = 14.61 (CH_3_), 14.64 (CH_3_), 63.84 (OCH_2_), 63.96 (OCH_2_), 112.37 (C-2”), 113.09 (C-5”), 116.52 (C-1”), 120.94 (C-6”), 123.52 (q, *J* = 274 Hz, CF_3_), 126.25 (q, *J* = 31.7 Hz, C-2), 126.71 (q, *J* = 4.9 Hz, C-3), 128.69 (C-6), 131.05 (C-4), 132.69 (C-5), 134.03 (m, C-1), 148.23 (C-3”), 149.00 (C-3’), 150.41 (C-4”), 150.72 (C-4’), 166.47 (C=O); HRMS (EI+) calcd for C_20_H_18_F_3_N_3_O_4_: 421.1249; found: 421.1248.

*N-(4-(3,4-Diethoxyphenyl)-1,2,5-oxadiazol-3-yl)-3-(trifluoromethyl)benzamide* (**13**): Method A: The reaction of compound **9** (249 mg (1.00 mmol)), 3-(trifluoromethyl)benzoyl chloride (271 mg (1.30 mmol)) and NaH (60% dispersion in mineral oil) (80 mg (2.00 mmol)) in dry DMF (16 mL) gave the raw carboxamide which was purified by recrystallization in CH_2_Cl_2_ to yield compound **13** as white crystals (131 mg (31%)). m.p. 153 °C. IR = 3203, 1675, 1498, 1378, 1336, 1260, 1217, 1172, 1121, 1042, 815; ^1^H NMR (DMSO-d_6_, 400 MHz) *δ* = 1.19 (t, *J* = 7.0 Hz, 3H, CH_3_), 1.32 (t, *J* = 7.0 Hz, 3H, CH_3_), 3.90 (q, *J* = 7.0 Hz, 2H, OCH_2_), 4.06 (q, *J* = 7.0 Hz, 2H, OCH_2_), 7.08 (d, *J* =8.3 Hz, 1H, 5”-H), 7.33 (d, *J* = 1.9 Hz, 1H, 2”-H), 7.34 (dd, *J* = 8.3, 1.9 Hz, 1H, 6”-H), 7.84 (t, *J* = 7.7 Hz, 1H, 5-H), 8.06 (d, *J* = 7.7 Hz, 1H, 4-H), 8.29 (d, *J* = 7.7 Hz, 1H, 6-H), 8.36 (s, 1H, 2-H), 11.53 (br, 1H, NH); ^13^C NMR (DMSO-d_6_, 100 MHz) *δ* = 14.40 (CH_3_), 14.56 (CH_3_), 63.77 (OCH_2_), 63.81 (OCH_2_), 111.72 (C-2”), 113.24 (C-5”), 116.83 (C-1”), 120.59 (C-6”), 123.82 (q, *J* = 272 Hz, CF_3_), 124.66 (q, *J* = 3.9 Hz, C-2), 129.33 (q, *J* = 4.4 Hz, C-4), 129.44 (q, *J* = 32.2 Hz, C-3), 130.18 (C-5), 132.27 (C-6), 133.21 (C-1), 148.11 (C-3”), 149.80 (C-3’), 150.36 (C-4”), 151.08 (C-4’), 165.25 (C=O); HRMS (EI+) calcd for C_20_H_18_F_3_N_3_O_4_: 421.1249; found: 421.1242.

*N-(4-(3,4-Diethoxyphenyl)-1,2,5-oxadiazol-3-yl)-3-fluorobenzamide* (**14**): Method A: The reaction of compound **9** (249 mg (1.00 mmol)), 3-fluorobenzoyl chloride (206 mg (1.30 mmol)) and NaH (60% dispersion in mineral oil) (80 mg (2.00 mmol)) in dry DMF (16 mL) gave the raw carboxamide which was purified by recrystallization in CH_2_Cl_2_ to yield compound **14** as beige crystals (186 mg (50%)). m.p. 250 °C. IR = 3216, 2983, 1663, 1591, 1505, 1364, 1275, 1215, 1142, 1039, 880, 857, 809; ^1^H NMR (DMSO-d_6_, 400 MHz) *δ* = 1.27 (t, *J* = 7.0 Hz, 3H, CH_3_), 1.33 (t, *J* = 7.0 Hz, 3H, CH_3_), 3.99 (q, *J* = 7.0 Hz, 2H, OCH_2_), 4.07 (q, *J* = 7.0 Hz, 2H, OCH_2_), 7.06 (d, *J* =8.4 Hz, 1H, 5”-H), 7.32 (td, *J* = 8.4, 2.1 Hz, 1H, 4-H), 7.48 (ddd, *J* = 8.4, 7.6, 5.7 Hz, 1H, 5-H), 7.63 (dd, *J* = 8.3, 1.4 Hz, 1H, 6”-H), 7.81-7.87 (m, 2H, 2-H, 2”-H), 7.93 (d, *J* = 7.6 Hz, 1H, 6-H); ^13^C NMR (DMSO-d_6_, 100 MHz) *δ* = 14.60 (CH_3_), 14.66 (CH_3_), 63.76 (2 OCH_2_), 112.34 (C-2”), 113.08 (C-5”), 114.69 (d, *J* = 22.1 Hz, C-2), 117.25 (d, *J* = 21.2 Hz, C-4), 119.08 (C-1”), 120.60 (C-6”), 124.25 (d, *J* = 2.7 Hz, C-6), 129.87 (d, *J* = 7.9 Hz, C-5), 140.35 (C-1), 147.84 (C-3”), 149.66 (C-4”), 150.19 (C-4’), 155.35 (C-3’), 161.94 (d, *J* = 242 Hz, C-3), 166.84 (C=O); HRMS (EI+) calcd for C_19_H_18_FN_3_O_4_: 371.1261; found: 371.1276.

*3-Chloro-N-(4-(3,4-diethoxyphenyl)-1,2,5-oxadiazol-3-yl)-4-methoxybenzamide* (**16**): Method B: The reaction of 3-chloro-4-methoxybenzoic acid (280 mg (1.50 mmol)) and oxalyl dichloride 2 M in CH_2_Cl_2_ (0.95 mL (1.90 mmol)) in dry CH_2_Cl_2_ (14 mL) gave the raw acid chloride which was suspended in dry DMF (9 mL) and added to a suspension of compound **9** (249 mg (1.00 mmol)) and NaH (60% dispersion in mineral oil) (80 mg (2.00 mmol)) in dry DMF (14 mL), giving the raw carboxamide which was purified by recrystallization in toluene to yield compound **16** as white crystals (255 mg (61%)). m.p. 260 °C (decomp.). IR = 2978, 1663, 1600, 1501, 1470, 1395, 1360, 1271, 1215, 1142, 1057, 1040, 937, 855, 811; ^1^H NMR (DMSO-d_6_, 400 MHz) *δ* = 1.30 (t, *J* = 7.0 Hz, 3H, CH_3_), 1.34 (t, *J* = 7.0 Hz, 3H, CH_3_), 3.91 (s, 3H, OCH_3_), 4.02 (q, *J* = 7.0 Hz, 2H, OCH_2_), 4.07 (q, *J* = 7.0 Hz, 2H, OCH_2_), 7.06 (d, *J* = 8.4 Hz, 1H, 5”-H), 7.18 (d, *J* = 8.7 Hz, 1H, 5-H), 7.65 (dd, *J* = 8.4, 1.6 Hz, 1H, 6”-H), 7.92 (d, *J* = 1.6 Hz, 1H, 2”-H), 8.05 (dd, *J* = 8.7, 1.9 Hz, 1H, 6-H), 8.17 (d, *J* = 1.6 Hz, 1H, 2-H), 11.27 (br, 1H, NH); ^13^C NMR (DMSO-d_6_, 100 MHz) *δ* = 14.69 (2 CH_3_), 56.27 (OCH_3_), 63.72 (2 OCH_2_), 111.80 (C-5), 112.33 (C-2”), 112.99 (C-5”), 119.43 (C-1”), 120.18 (C-3), 120.55 (C-6”), 128.55 (C-6), 129.79 (C-2), 131.72 (C-1), 147.76 (C-3”), 149.48 (C-4”), 150.09 (C-4’), 153.07 (C-3’), 156.03 (C-4), 166.89 (C=O); HRMS (EI+) calcd for C_20_H_20_ClN_3_O_5_: 417.1092; found: 417.1081.

*N-(4-(3,4-Diethoxyphenyl)-1,2,5-oxadiazol-3-yl)-3-fluoro-4-methoxybenzamide* (**17**): Method B: The reaction of 3-fluoro-4-methoxybenzoic acid (255 mg (1.50 mmol)) and oxalyl dichloride 2 M in CH_2_Cl_2_ (0.95 mL (1.90 mmol)) in dry CH_2_Cl_2_ (14 mL) gave the raw acid chloride which was suspended in dry DMF (9 mL) and added to a suspension of compound **9** (249 mg (1.00 mmol)) and NaH (60% dispersion in mineral oil) (80 mg (2.00 mmol)) in dry DMF (14 mL), giving the raw carboxamide which was purified by recrystallization in toluene to yield compound **17** as beige crystals (124 mg (31%)). m.p. 232 °C (decomp.). IR = 3199, 2982, 1663, 1615, 1500, 1469, 1396, 1377, 1327, 1286, 1217, 1140, 1040, 853, 802; ^1^H NMR (DMSO-d_6_, 400 MHz) *δ* = 1.25 (t, *J* = 6.9 Hz, 3H, CH_3_), 1.33 (t, *J* = 6.9 Hz, 3H, CH_3_), 3.92 (s, 3H, OCH_3_), 3.96 (q, *J* = 6.9 Hz, 2H, OCH_2_), 4.07 (q, *J* = 6.9 Hz, 2H, OCH_2_), 7.06 (d, *J* = 8.6 Hz, 1H, 5”-H), 7.27 (t, *J* = 8.7 Hz, 1H, 5-H), 7.51 (dd, *J* = 8.4, 2.0 Hz, 1H, 6”-H), 7.62 (d, *J* = 2.0 Hz, 1H, 2”-H), 7.89 (d, *J* = 8.3 Hz, 1H, 6-H), 7.90 (s, 1H, 2-H), 11.29 (br, 1H, NH); ^13^C NMR (DMSO-d_6_, 100 MHz) *δ* = 14.55 (CH_3_), 14.63 (CH_3_), 56.23 (d, *J* = 2.3 Hz, OCH_3_), 63.74 (2 OCH_2_), 112.02 (C-2”), 113.06 (C-5, C-5”), 115.54 (d, *J* = 19.0 Hz, C-2), 118.23 (C-1”), 120.50 (C-6”), 125.30 (d, *J* = 3.1 Hz, C-6), 128.22 (C-1), 147.90 (C-3”), 149.86 (d, *J* = 23.6 Hz, C-4), 149.89 (C-4”), 150.61 (C-4’), 150.76 (d, *J* = 244 Hz, C-3), 153.19 (C-3’), 166.11 (C=O); HRMS (EI+) calcd for C_20_H_20_FN_3_O_5_: 401.1387; found: 401.1380.

*N-(4-(3,4-Diethoxyphenyl)-1,2,5-oxadiazol-3-yl)-4-((pyrrolidin-1-yl)methyl)benzamide* (**26**): Method B: The reaction of carboxylic acid **20** (308 mg (1.50 mmol)) and oxalyl dichloride 2 M in CH_2_Cl_2_ (0.95 mL (1.90 mmol)) in dry CH_2_Cl_2_ (14 mL) gave the raw acid chloride which was suspended in dry DMF (9 mL) and added to a suspension of compound **9** (249 mg (1.00 mmol)) and NaH (60% dispersion in mineral oil) (80 mg (2.00 mmol)) in dry DMF (14 mL), giving the raw carboxamide which was purified by recrystallization in toluene to yield compound **26** as light yellow crystals (140 mg (32%)). m.p. 251 °C (decomp.). IR = 2973, 2788, 1584, 1471, 1365, 1271, 1246, 1201, 1141, 1091, 1038, 997, 937, 880, 854, 809; ^1^H NMR (DMSO-d_6_, 400 MHz) *δ* = 1.32 (t, *J* = 7.0 Hz, 3H, CH_3_), 1.35 (t, *J* = 7.0 Hz, 3H, CH_3_), 1.69 (br, s, 4H, (CH_2_)_2_), 2.43 (br, s, 4H, N(CH_2_)_2_), 3.59 (s, 2H, ArCH_2_), 4.05 (q, *J* = 7.0 Hz, 2H, OCH_2_), 4.08 (q, *J* = 7.0 Hz, 2H, OCH_2_), 7.07 (d, *J* = 8.5 Hz, 1H, 5”-H), 7.27 (d, *J* = 7.8 Hz, 2H, 3-H, 5-H), 7.92 (dd, *J* = 7.8, 1.9 Hz, 1H, 6”-H), 8.11 (d, *J* = 7.8 Hz, 2H, 2-H, 6-H), 8.24 (d, *J* = 1.9 Hz, 1H, 2”-H); ^13^C NMR (DMSO-d_6_, 100 MHz) *δ* = 14.74 (CH_3_), 14.79 (CH_3_), 23.14 ((CH_2_)_2_), 53.54 (N(CH_2_)_2_), 59.54 (ArCH_2_), 63.68 (2 OCH_2_), 112.67 (C-2”), 112.86 (C-5”), 120.64 (C-1”), 120.72 (C-6”), 127.37 (C-3, C-5), 128.24 (C-2, C-6), 139.78 (C-1), 140.34 (C-4), 147.60 (C-3”), 149.12 (C-4”), 149.56 (C-4’), 158.93 (C-3’), 169.56 (C=O); HRMS (EI+) calcd for C_24_H_28_N_4_O_4_: 436.2111; found: 436.2102.

*N-(4-(3,4-Diethoxyphenyl)-1,2,5-oxadiazol-3-yl)-4-((morpholin-4-yl)methyl)benzamide* (**27**): Method B: The reaction of carboxylic acid **25** (332 mg (1.50 mmol)) and oxalyl dichloride 2 M in CH_2_Cl_2_ (0.95 mL (1.90 mmol)) in dry CH_2_Cl_2_ (14 mL) gave the raw acyl chloride which was suspended in dry DMF (9 mL) and added to a suspension of compound **9** (249 mg (1.00 mmol)) and NaH (60% dispersion in mineral oil) (80 mg (2.00 mmol)) in dry DMF (14 mL), giving the raw carboxamide which was purified by recrystallization in toluene to yield compound **27** as pale yellow crystals (258 mg (57%)). m.p. 269 °C (decomp.). IR = 2977, 1585, 1499, 1472, 1360, 1271, 1202, 1141, 1109, 1035, 867; ^1^H NMR (DMSO-d_6_, 400 MHz) *δ* = 1.32 (t, *J* = 7.0 Hz, 3H, CH_3_), 1.35 (t, *J* = 7.0 Hz, 3H, CH_3_), 2.36 (br, s, 4H, N(CH_2_)_2_), 3.49 (s, 2H, ArCH_2_), 3.58 (t, *J* = 4.5 Hz, 4H, O(CH_2_)_2_), 4.05 (q, *J* = 7.0 Hz, 2H, OCH_2_), 4.08 (q, *J* = 7.0 Hz, 2H, OCH_2_), 7.07 (d, *J* = 8.6 Hz, 1H, 5”-H), 7.28 (d, *J* = 8.2 Hz, 2H, 3-H, 5-H), 7.92 (dd, *J* = 8.6, 1.6 Hz, 1H, 6”-H), 8.12 (d, *J* = 7.8 Hz, 2H, 2-H, 6-H), 8.23 (d, *J* = 1.6 Hz, 1H, 2”-H); ^13^C NMR (DMSO-d_6_, 100 MHz) *δ* = 14.75 (CH_3_), 14.79 (CH_3_), 53.22 (N(CH_2_)_2_), 62.36 (ArCH_2_), 63.69 (2 OCH_2_), 66.24 (O(CH_2_)_2_), 112.66 (C-2”), 112.86 (C-5”), 120.64 (C-1”), 120.73 (C-6”), 127.86 (C-3, C-5), 128.28 (C-2, C-6), 138.60 (C-1), 140.08 (C-4), 147.61 (C-3”), 149.12 (C-4”), 149.56 (C-4’), 158.97 (C-3’), 169.47 (C=O); HRMS (EI+) calcd for C_24_H_28_N_4_O_5_: 452.2060; found: 452.2043.

*N-(4-(3,4-Diethoxyphenyl)-1,2,5-oxadiazol-3-yl)-3-((pyrrolidin-1-yl)methyl)benzamide* (**28**): Method B: The reaction of carboxylic acid **20** (308 mg (1.50 mmol)) and oxalyl dichloride 2 M in CH_2_Cl_2_ (0.95 mL (1.90 mmol)) in dry CH_2_Cl_2_ (14 mL) gave the raw acid chloride which was suspended in dry DMF (9 mL) and added to a suspension of compound **9** (249 mg (1.00 mmol)) and NaH (60% dispersion in mineral oil) (80 mg (2.00 mmol)) in dry DMF (14 mL), giving the raw carboxamide which was purified by column chromatography (silica gel, CH_2_Cl_2_/toluene/*Me*OH, 20:1:1) to yield compound **28** as yellow oil (262 mg (60%)). IR = 3212, 2974, 2799, 1661, 1591, 1500, 1473, 1395, 1377, 1259, 1217, 1140, 1041, 849, 800; ^1^H NMR (CDCl_3_, 400 MHz) *δ* = 1.40 (t, *J* = 7.0 Hz, 3H, CH_3_), 1.46 (t, *J* = 7.0 Hz, 3H, CH_3_), 1.78–1.81 (m, 4H, (CH_2_)_2_), 2.52–2.56 (m, 4H, N(CH_2_)_2_), 3.69 (s, 2H, ArCH_2_), 4.04 (q, *J* = 7.0 Hz, 2H, OCH_2_), 4.11 (q, *J* = 7.0 Hz, 2H, OCH_2_), 6.91 (d, *J* = 8.2 Hz, 1H, 5”-H), 7.23 (dd, *J* = 8.2, 1.9 Hz, 1H, 6”-H), 7.25 (d, *J* = 1.9 Hz, 1H, 2”-H), 7.42 (t, *J* = 7.7 Hz, 1H, 5-H), 7.57 (d, *J* = 7.6 Hz, 1H, 4-H), 7.75 (d, *J* = 7.8 Hz, 1H, 6-H), 7.87 (s, 1H, 2-H); ^13^C NMR (CDCl_3_, 100 MHz) *δ* = 14.64 (CH_3_), 14.65 (CH_3_), 23.42 ((CH_2_)_2_), 54.20 (N(CH_2_)_2_), 60.09 (ArCH_2_), 64.44 (OCH_2_), 64.58 (OCH_2_), 112.36 (C-2”), 112.94 (C-5”), 117.28 (C-1”), 120.33 (C-6”), 126.40 (C-6), 127.82 (C-2), 128.92 (C-5), 132.39 (C-1), 133.45 (C-4), 140.34 (C-3), 148.88 (C-3’), 149.08 (C-3”), 149.83 (C-4’), 150.82 (C-4”), 165.69 (C=O); HRMS (EI+) calcd for C_24_H_28_N_4_O_4_: 436.2111; found: 436.2086.

*N-(4-(3,4-Diethoxyphenyl)-1,2,5-oxadiazol-3-yl)-3-((morpholin-4-yl)methyl)benzamide* (**29**): Method B: The reaction of carboxylic acid **21** (332 mg (1.50 mmol)) and oxalyl dichloride 2 M in CH_2_Cl_2_ (0.95 mL (1.90 mmol)) in dry CH_2_Cl_2_ (14 mL) gave the raw acid chloride which was suspended in dry DMF (9 mL) and added to a suspension of compound **9** (249 mg (1.00 mmol)) and NaH (60% dispersion in mineral oil) (80 mg (2.00 mmol)) in dry DMF (14 mL), giving the raw carboxamide which was purified by recrystallization in toluene to yield compound **29** as white crystals (113 mg (25%)). m.p. 199 °C. IR = 2978, 1605, 1502, 1468, 1361, 1271, 1209, 1141, 1114, 1036, 854; ^1^H NMR (CD_3_OD, 400 MHz) *δ* = 1.28 (t, *J* = 7.0 Hz, 3H, CH_3_), 1.40 (t, *J* = 7.0 Hz, 3H, CH_3_), 2.48 (br, s, 4H, N(CH_2_)_2_), 3.58 (s, 2H, ArCH_2_), 3.70 (t, *J* = 4.5 Hz, 4H, O(CH_2_)_2_), 3.94 (q, *J* = 7.0 Hz, 2H, OCH_2_), 4.09 (q, *J* = 7.0 Hz, 2H, OCH_2_), 6.98 (d, *J* = 8.3 Hz, 1H, 5”-H), 7.36 (t, *J* = 7.7 Hz, 1H, 5-H), 7.43 (d, *J* = 7.7 Hz, 1H, 4-H), 7.64 (dd, *J* = 8.3, 1.6 Hz, 1H, 6”-H), 7.72 (d, *J* = 1.6 Hz, 1H, 2”-H), 8.00 (d, *J* = 7.7 Hz, 1H, 6-H), 8.03 (s, 1H, 2-H); ^13^C NMR (CD_3_OD, 100 MHz) *δ* = 15.23 (2 CH_3_), 54.77 (N(CH_2_)_2_), 64.49 (ArCH_2_), 65.72 (OCH_2_), 65.76 (OCH_2_), 67.92 (O(CH_2_)_2_), 114.03 (C-2”), 114.47 (C-5”), 121.81 (C-1”), 122.20 (C-6”), 128.81 (C-6), 128.95 (C-5), 131.01 (C-2), 132.31 (C-4), 137.99 (C-3), 140.85 (C-1), 149.92 (C-3”), 151.48 (C-4”), 151.79 (C-4’), 160.84 (C-3’), 173.71 (C=O); HRMS (EI+) calcd for C_24_H_28_N_4_O_5_: 452.2060; found: 452.2042.

*N-(4-(3,4-Diethoxyphenyl)-1,2,5-oxadiazol-3-yl)-3-(trifluoromethoxy)benzamide* (**15**): Method C: The reaction of 3-(trifluoromethoxy)benzoic acid (309 mg (1.50 mmol)), *N*-hydroxysuccinimide (182 mg (1.58 mmol)) and *N*,*N*′-dicyclohexylcarbodiimide (310 mg (1.50 mmol)) in dry THF (10 mL) gave the raw NHS ester. It was suspended in dry DMF (4 mL) and added to a suspension of compound **9** (249 mg (1.00 mmol)) and NaH (60% dispersion in mineral oil) (80 mg (2.00 mmol)) in dry DMF (14 mL), giving the raw carboxamide which was purified by recrystallization in CH_2_Cl_2_ to yield compound **15** as white crystals (136 mg (31%)). m.p. 145–147 °C. IR = 3209, 1675, 1590, 1501, 1378, 1260, 1216, 1170, 1042, 809, 633; ^1^H NMR (DMSO-d_6_, 400 MHz) *δ* = 1.19 (t, *J* = 7.0 Hz, 3H, CH_3_), 1.32 (t, *J* = 7.0 Hz, 3H, CH_3_), 3.90 (q, *J* = 7.0 Hz, 2H, OCH_2_), 4.05 (q, *J* = 7.0 Hz, 2H, OCH_2_), 7.06 (d, *J* = 8.3 Hz, 1H, 5”-H), 7.39–7.44 (m, 2H, 2”-H, 6”-H), 7.63 (d, *J* = 8.3 Hz, 1H, 4-H), 7.68 (t, *J* = 7.8 Hz, 1H, 5-H), 7.96 (s, 1H, 2-H), 8.06 (d, *J* = 7.5 Hz, 1H, 6-H), 11.32 (br, 1H, NH); ^13^C NMR (DMSO-d_6_, 100 MHz) *δ* = 14.45, 14.62 (2 CH_3_), 63.72, 63.78 (2 OCH_2_), 111.79 (C-2”), 113.13 (C-5”), 117.50 (C-1”), 120.08 (q, *J* = 257 Hz, CF_3_), 120.50 (C-2), 120.56 (C-6”), 124.68 (C-4), 127.27 (C-6), 130.75 (C-5), 136.13 (C-1), 148.01 (C-3”), 148.34 (C-3’), 150.14 (C-4”), 150.84 (C-4’), 151.53 (C-3), 165.46 (C=O); HRMS (EI+) calcd for C_20_H_18_F_3_N_3_O_5_: 437.1198; found: 437.1171.

#### 3.2.4. General Procedure for the Synthesis of Compounds **31**, **32**, **34** and **35**

Amino furazan **9** (1.00 mmol) was dissolved in a mixture of dry diethyl ether (4 mL) and dry benzene (16 mL). Dry pyridine (1.13 mmol) was added and then a solution of chloroacyl chloride (1.10 mmol) in dry diethyl ether (4 mL) was added dropwise. The mixture was refluxed for 20 h and then the solvent was evaporated in vacuo. The residue was mixed with water (35 mL) and the aqueous phase was extracted with CH_2_Cl_2_. The combined organic layers were dried over anhydrous sodium sulfate and filtered, and the solvent was evaporated in vacuo, yielding the raw carboxamide which was used without further purification. It was dissolved in dry acetonitrile (20 mL), and the respective amine (2.00 mmol), K_2_CO_3_ (2.00 mmol) and a catalytical amount of NaI (0.65 mmol) were added. The mixture was stirred at 70 °C for 20 h and then cooled to ambient temperature. The suspension was filtered and the filtrate was evaporated in vacuo to dryness. The residue was mixed with water (20 mL) and the aqueous phase was extracted with CH_2_Cl_2_. The organic layer was washed with 8% aqueous NaHCO_3_ and brine, dried over anhydrous sodium sulfate and filtered, and the solvent was evaporated in vacuo, yielding the carboxamide, which was purified by column chromatography.

*N-(4-(3,4-Diethoxyphenyl)-1,2,5-oxadiazol-3-yl)-2-(pyrrolidin-1-yl)acetamide* (**31**): The reaction of amino furazan **9** (249 mg (1.00 mmol)), chloroacetyl chloride (124 mg (1.10 mmol)) and pyridine (89 g (1.13 mmol)) in a mixture of dry diethyl ether (8 mL) and dry benzene (16 mL) gave the raw carboxamide. This was mixed with pyrrolidine (142 mg (2.00 mmol)), K_2_CO_3_ (276 mg (2.00 mmol)) and NaI (97 mg (0.65 mmol)) in dry acetonitrile (20 mL), giving the tertiary amine which was purified by column chromatography (silica gel, CH_2_Cl_2_/*Me*OH, 100:1) to yield compound **31** as pale yellow amorphous solid (87 mg (24%)). IR = 2983, 1726, 1593, 1545, 1519, 1461, 1256, 1216, 1149, 1042, 844, 811; ^1^H NMR (CDCl_3_, 400 MHz) *δ* = 1.48 (t, *J* = 7.0 Hz, 3H, CH_3_), 1.50 (t, *J* = 7.0 Hz, 3H, CH_3_), 1.79–1.85 (m, 4H, (CH_2_)_2_), 2.68–2.73 (m, 4H, N(CH_2_)_2_), 3.38 (s, 2H, CH_2_CO), 4.13 (q, *J* = 7.0 Hz, 2H, OCH_2_), 4.17 (q, *J* = 7.0 Hz, 2H, OCH_2_), 6.97 (d, *J* = 8.4 Hz, 1H, 5”-H), 7.12 (dd, *J* = 8.4, 2.1 Hz, 1H, 6”-H), 7.22 (d, *J* = 2.1 Hz, 1H, 2”-H), 9.73 (br, 1H, NH); ^13^C NMR (CDCl_3_, 100 MHz) *δ* = 14.63 (CH_3_), 14.70 (CH_3_), 24.04 ((CH_2_)_2_), 54.62 (N(CH_2_)_2_), 58.76 (CH_2_), 64.63, 64.88 (2 OCH_2_), 113.07 (C-5”), 113.13 (C-2”), 117.07 (C-1”), 120.06 (C-6”), 147.54 (C-3’), 148.36 (C-4’), 149.53 (C-3”), 151.07 (C-4”), 168.87 (C=O); HRMS (EI+) calcd for C_18_H_24_N_4_O_4_: 360.1797; found: 360.1782.

*N-(4-(3,4-Diethoxyphenyl)-1,2,5-oxadiazol-3-yl)-2-(morpholin-4-yl)acetamide* (**32**): The reaction of amino furazan **9** (249 mg (1.00 mmol)), chloroacetyl chloride (124 mg (1.10 mmol)) and pyridine (89 g (1.13 mmol)) in a mixture of dry diethyl ether (8 mL) and dry benzene (16 mL) gave the raw carboxamide. It was mixed with morpholine (174 mg (2.00 mmol)), K_2_CO_3_ (276 mg (2.00 mmol)) and NaI (97 mg (0.65 mmol)) in dry acetonitrile (20 mL), giving the tertiary amine which was purified by column chromatography (silica gel, CH_2_Cl_2_/*Me*OH, 40:1) to yield compound **32** as white amorphous solid (233 mg (62%)). IR = 3326, 2982, 1731, 1595, 1542, 1520, 1462, 1328, 1254, 1216, 1146, 1112, 1041, 1009, 867, 809; ^1^H NMR (CDCl_3_, 400 MHz) *δ* = 1.46–1.53 (m, 6H, 2 CH_3_), 2.64 (t, *J* = 4.6 Hz, 4H, N(CH_2_)_2_), 3.24 (s, 2H, 2-H), 3.68 (br, t, *J* = 4.6 Hz, 4H, O(CH_2_)_2_), 4.12–4.20 (m, 4H, 2 OCH_2_), 7.00 (d, *J* = 8.4 Hz, 1H, 5”-H), 7.11 (dd, *J* = 8.4, 2.0 Hz, 1H, 6”-H), 7.23 (d, *J* = 2.0 Hz, 1H, 2”-H), 9.75 (br, s, 1H, NH); ^13^C NMR (CDCl_3_, 100 MHz) *δ* = 14.62 (CH_3_), 14.71 (CH_3_), 53.79 (N(CH_2_)_2_), 61.76 (C-2), 64.69, 65.01 (2 OCH_2_), 66.85 (O(CH_2_)_2_), 113.05 (C-5”), 113.57 (C-2”), 116.84 (C-1”), 120.00 (C-6”), 147.42 (C-3’), 148.30 (C-4’), 149.65 (C-3”), 151.29 (C-4”), 167.80 (C=O); HRMS (EI+) calcd for C_18_H_24_N_4_O_5_: 376.1747; found: 376.1743.

*N-(4-(3,4-Diethoxyphenyl)-1,2,5-oxadiazol-3-yl)-3-(pyrrolidin-1-yl)propanamide* (**34**): The reaction of amino furazan **9** (249 mg (1.00 mmol)), 3-chloropropanoyl chloride (140 mg (1.10 mmol)) and pyridine (89 g (1.13 mmol)) in a mixture of dry diethyl ether (8 mL) and dry benzene (16 mL) gave the raw carboxamide. It was mixed with pyrrolidine (142 mg (2.00 mmol)), K_2_CO_3_ (276 mg (2.00 mmol)) and NaI (97 mg (0.65 mmol)) in dry acetonitrile (20 mL), giving the tertiary amine which was purified by column chromatography (silica gel, CH_2_Cl_2_/*Me*OH, 15:1) to yield compound **34** as yellow amorphous solid (300 mg (80%)). IR = 2977, 1719, 1551, 1476, 1434, 1396, 1323, 1274, 1250, 1217, 1149, 1043, 916, 845; ^1^H NMR (CDCl_3_, 400 MHz) *δ* = 1.46–1.51 (m, 6H, 2 CH_3_), 1.53–1.57 (m, 4H, (CH_2_)_2_), 2.51–2.56 (m, 4H, N(CH_2_)_2_), 2.63 (t, *J* = 5.9 Hz, 2H, 2-H), 2.82 (t, *J* = 5.9 Hz, 2H, 3-H), 4.10–4.18 (m, 4H, 2 OCH_2_), 6.96 (d, *J* = 8.3 Hz, 1H, 5”-H), 7.11 (dd, *J* = 8.3, 1.8 Hz, 1H, 6”-H), 7.19 (d, *J* = 1.8 Hz, 1H, 2”-H), 12.02 (br, 1H, NH); ^13^C NMR (CDCl_3_, 100 MHz) *δ* = 14.68 (CH_3_), 14.72 (CH_3_), 23.35 ((CH_2_)_2_), 34.06 (C-2), 51.07 (C-3), 53.08 (N(CH_2_)_2_), 64.89, 64.65 (2 OCH_2_), 112.95 (C-5”), 113.50 (C-2”), 117.36 (C-1”), 120.46 (C-6”), 148.42 (C-3’), 148.71 (C-4’), 149.27 (C-3”), 150.88 (C-4”), 170.64 (C=O); HRMS (EI+) calcd for C_19_H_26_N_4_O_4_: 374.1954; found: 374.1973.

*N-(4-(3,4-Diethoxyphenyl)-1,2,5-oxadiazol-3-yl)-3-(morpholin-4-yl)propanamide* (**35**): The reaction of amino furazan **9** (249 mg (1.00 mmol)), 3-chloropropanoyl chloride (140 mg (1.10 mmol)) and pyridine (89 g (1.13 mmol)) in a mixture of dry diethyl ether (8 mL) and dry benzene (16 mL) gave the raw carboxamide. It was mixed with morpholine (174 mg (2.00 mmol)), K_2_CO_3_ (276 mg (2.00 mmol)) and NaI (97 mg (0.65 mmol)) in dry acetonitrile (20 mL), giving the tertiary amine which was purified by column chromatography (silica gel, CH_2_Cl_2_/*Me*OH, 40:1) to yield compound **35** as white amorphous solid (223 mg (57%)). IR = 3205, 2978, 1677, 1537, 1472, 1375, 1274, 1215, 1142, 1119, 1042, 870, 814; ^1^H NMR (CDCl_3_, 400 MHz) *δ* = 1.48 (q, *J* = 6.9 Hz, 6H, 2 CH_3_), 2.49 (br, t, *J* = 4.6 Hz, 4H, N(CH_2_)_2_), 2.64 (t, *J* = 5.7 Hz, 2H, 2-H), 2.72 (t, *J* = 5.7 Hz, 2H, 3-H), 3.36–3.41 (m, 4H, O(CH_2_)_2_), 4.10–4.17 (m, 4H, 2 OCH_2_), 6.96 (d, *J* = 8.2 Hz, 1H, 5”-H), 7.15 (dd, *J* = 8.2, 2.1 Hz, 1H, 6”-H), 7.19 (d, *J* = 2.1 Hz, 1H, 2”-H), 11.28 (br, 1H, NH); ^13^C NMR (CDCl_3_, 100 MHz) *δ* = 14.62 (CH_3_), 14.70 (CH_3_), 31.56 (C-2), 52.70 (N(CH_2_)_2_), 53.56 (C-3), 64.63 (OCH_2_), 64.98 (OCH_2_), 66.17 (O(CH_2_)_2_), 113.09 (C-5”), 113.56 (C-2”), 117.09 (C-1”), 120.83 (C-6”), 148.22 (C-3’), 148.95 (C-4’), 149.28 (C-3”), 151.14 (C-4”), 170.19 (C=O); HRMS (EI+) calcd for C_19_H_26_N_4_O_5_: 390.1903; found: 390.1892.

*3-Methyl-N-(4-phenyl-1,2,5-oxadiazol-3-yl)benzamide* (**39**): Method A: The reaction of compound **38** (161 mg (1.00 mmol)), 3-methylbenzoyl chloride (201 mg (1.30 mmol)) and NaH (60% dispersion in mineral oil) (80 mg (2.00 mmol)) in dry DMF (16 mL) gave the raw carboxamide which was purified by column chromatography (aluminium oxide basic, cyclohexane/ethyl acetate, 3:1) to yield compound **39** as white amorphous solid (75 mg (27%)). IR = 3288, 1663, 1588, 1566, 1521, 1481, 1383, 1284, 885, 807; ^1^H NMR (CDCl_3_, 400 MHz) *δ* = 2.41 (s, 3H, CH_3_), 7.38 (t, J = 7.5 Hz, 1H, 5-H), 7.42 (d, J = 7.5 Hz, 1H, 4-H), 7.45–7.53 (m, 3H, 3”-H, 4”-H, 5”-H), 7.63 (d, J = 7.5 Hz, 1H, 6-H), 7.69 (s, 1H, 2-H), 7.70 (d, J = 7.5 Hz, 2H, 2”-H, 6”-H), 8.29 (s, 1H, NH); ^13^C NMR (CDCl_3_, 100 MHz) *δ* = 21.32 (CH_3_), 124.45 (C-6), 125.29 (C-1”), 127.57 (C-2”, C-6”), 128.39 (C-2), 128.88 (C-5), 129.32 (C-3”, C-5”), 130.81 (C-4”), 131.99 (C-1), 133.95 (C-4), 139.13 (C-3), 148.73 (C-3’), 149.94 (C-4’), 165.38 (CO); HRMS (EI+) calcd for C_16_H_13_N_3_O_2_: 279.1008; found: 279.0997.

### 3.3. Biological Tests

#### 3.3.1. In Vitro Microplate Assay against *P. falciparum*

In vitro activity against erythrocytic stages of *P. falciparum* was determined using a ^3^H-hypoxanthine incorporation assay [45,46], using the drug-sensitive NF54 strain [47] or the chloroquine- and pyrimethamine-resistant K_1_ strain [48]. Chloroquine (Sigma C6628) was used as standard. Compounds were dissolved in DMSO at 10 mg/mL and added to parasite cultures incubated in RPMI 1640 medium without hypoxanthine, supplemented with HEPES (5.94 g/L), NaHCO_3_ (2.1 g/L), neomycin (100 U/mL), Albumax (5 g/L) and washed human red cells A^+^ at 2.5% hematocrit (0.3% parasitemia). Serial drug dilutions of 11 3-fold dilution steps covering a range from 100 to 0.002 μg/mL were prepared. The 96-well plates were incubated in a humidified atmosphere at 37 °C; 4% CO_2_, 3% O_2_, 93% N_2_. After 48 h, 0.05 mL of ^3^H-hypoxanthine (=0.5 μCi) was added to each well of the plate. The plates were incubated for a further 24 h under the same conditions. The plates were then harvested with a Betaplate cell harvester (Wallac, Zurich, Switzerland). The red blood cells were transferred onto a glass fiber filter and washed with distilled water. The dried filters were inserted into a plastic foil with 10 mL of scintillation fluid and counted in a Betaplate liquid scintillation counter (Wallac, Zurich, Switzerland). IC_50_ values were calculated from sigmoidal inhibition curves by linear regression [49] using Microsoft Excel. Artemisinin and chloroquine were used as control. Dose-response curves of measured compounds and activities with standard deviation values are available in Supplementary Materials Section (Figures S24–S41 and Table S1).

#### 3.3.2. In Vitro Cytotoxicity with L-6 Cells

Assays were performed in 96-well microtiter plates, each well containing 0.1 mL of RPMI 1640 medium supplemented with 1% L-glutamine (200 mM) and 10% fetal bovine serum and 4000 L-6 cells (a primary cell line derived from rat skeletal myoblasts) [50,51]. Serial drug dilutions of 11 3-fold dilution steps covering a range from 100 to 0.002 μg/mL were prepared. After 70 h of incubation, the plates were inspected under an inverted microscope to assure the growth of the controls and sterile conditions. Then, 0.01 mL of Alamar Blue was then added to each well and the plates incubated for another 2 h. Then, the plates were read with a Spectramax Gemini XS microplate fluorometer (Molecular Devices Cooperation, Sunnyvale, CA, USA) using an excitation wavelength of 536 nm and an emission wavelength of 588 nm. The IC_50_ values were calculated by linear regression [44] from the sigmoidal dose inhibition curves using SoftmaxPro software (Molecular Devices Cooperation, Sunnyvale, CA, USA). Podophyllotoxin (Sigma P4405) was used as control.

#### 3.3.3. Parallel Artificial Membrane Permeability Assay (PAMPA)

The PAMPA was performed using a 96-well precoated Corning Gentest PAMPA plate at a pH of 7.4. The PAMPA plate system consists of a donor plate and an acceptor plate. When both plates are coupled, each well is divided into two chambers that are separated by a lipid–oil–lipid trilayer constructed in a porous filter. Stock solutions (10 mM) of each compound were prepared in DMSO and then further diluted to a concentration of 200 µM in phosphate-buffered saline (PBS) at pH 7.4. The compound dissolved in PBS was then added to the donor plate and pure PBS was added to the acceptor plate. Four replicates of each compound and negative control (PBS) were transferred into different wells of the donor plate. Both plates were coupled and left at room temperature for 5 h. Then, the plates were separated, and the solutions of each donor well and acceptor well were transferred to 96-well UV-Star Microplates (Greiner Bio-One). The UV absorbance of compounds in donor wells and acceptor wells were analyzed by a SpectraMax M3 UV plate reader (Molecular Devices). The concentrations were received from a calibration curve for each substance. The plates were analyzed at a wavelength where the R^2^ value of the calibration curve was higher than 0.99 [34]. The effective permeability (P_e_) was calculated as shown in the following Equations (1)–(3):

(1) $$P_{e}\left(nm/s\right)=10^{7}*\frac{-\ln\left[1-\frac{c_{A}\left(t\right)}{c_{equ}}\right]}{S*\left(\frac{1}{V_{D}}+\frac{1}{V_{A}}\right)*\;t}$$

where:

*P_e_*—effective permeability;

*S*—filter area (0.3 cm^2^);

*V_D_*—donor well volume;

*V_A_*—acceptor well volume;

*t*—incubation time (14,400 s);

*c_A_*(*t*)—concentration of compound in acceptor well at time *t*;

*c_equ_*—equilibrium concentration.

(2) $$c_{equ}=\frac{\left[c_{D}\left(t\right)\;*V_{D}+c_{A}\left(t\right)\;*V_{A}\right]}{\left(V_{D}+V_{A}\right)}$$

where:

*c_D_*(*t*) —concentration of compound in donor well at time *t*.

Recovery of compounds from donor and acceptor wells (mass retention) was calculated as shown in the equation below. Data were only accepted when recovery exceeded 70%.

(3) $$R=1-\frac{\left[c_{D}\left(t\right)\;*V_{D}+c_{A}\left(t\right)\;*V_{A}\right]}{\left(c_{0}*V_{D}\right)}$$

where:

*R*—mass retention (%);

*c_D_*(*t*)—concentration of compound in donor well at time *t*;

*c_A_*(*t*)—concentration of compound in acceptor well at time *t*;

*c*_0_—initial concentration of compound in donor well;

*V_D_*—donor well volume;

*V_A_*—acceptor well volume.

#### 3.3.4. Ligand Efficiency (LE)

Ligand efficiency was calculated as shown in the following Equation (4) [33]:

(4) $$\mathrm{LE}=\frac{1.37}{\mathrm{HA}}*{\mathrm{pIC}}_{50}$$

where:

LE—ligand efficiency;

HA—number of heavy atoms;

pIC_50_—negative logarithm of IC_50_.

## 4. Conclusions

**Scheme 6:**
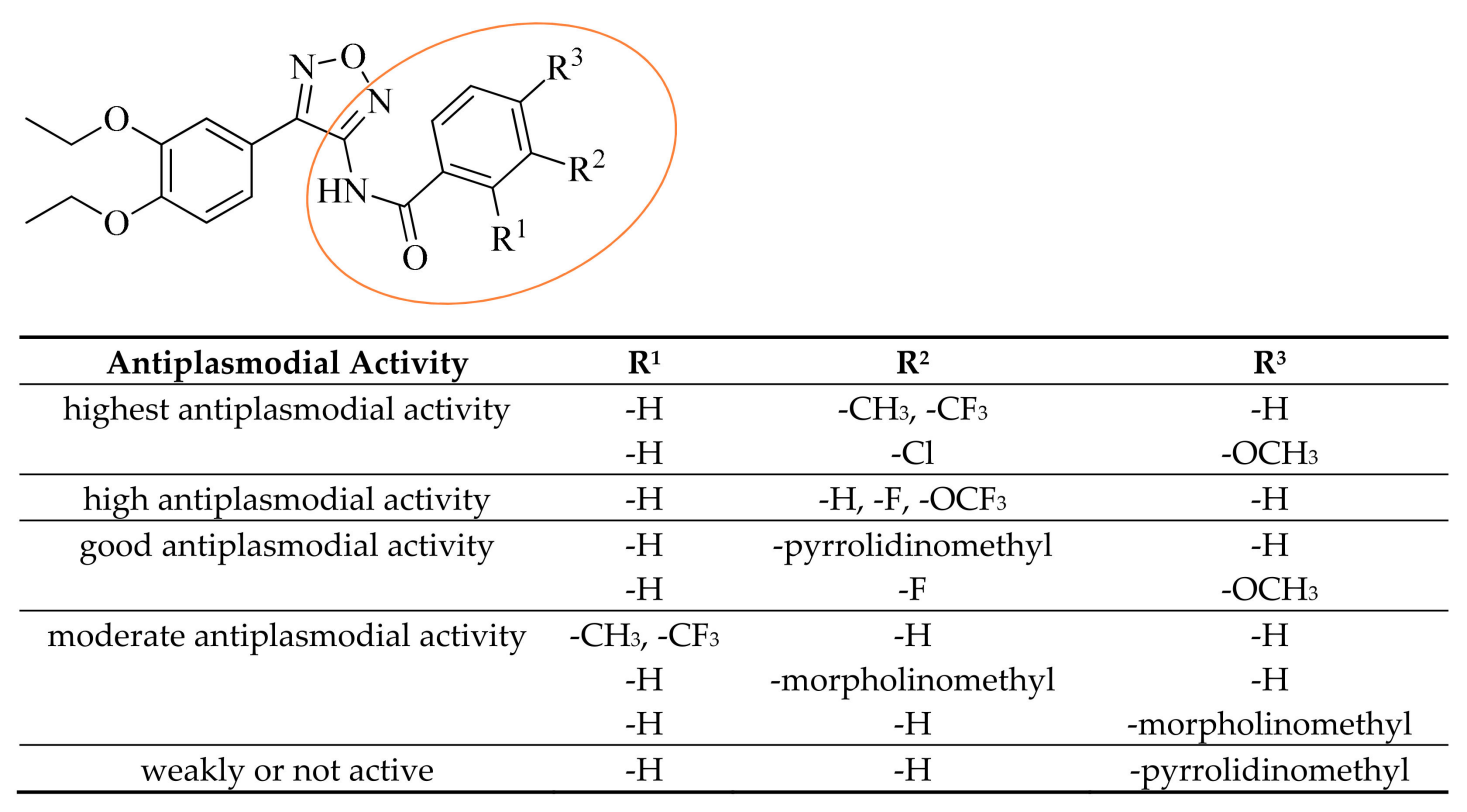
Summary of SARs of benzoyl derivatives.

This paper deals with the synthesis and antiplasmodial activities of a series of new 4-substituted *N*-acyl derivatives of 1,2,5-oxadiazol-3-amines. The substitution of the phenyl ring in ring position 4 of the furazan ring has a remarkable impact on the antiplasmodial activity. A compound with an unsubstituted ring was practically ineffective against both strains of *P. falciparum*, whereas its 3,4-diethoxy analog was active in low nanomolar concentration. Moreover, the activity strongly depended on the nature of the acyl moiety. Benzoyl derivatives were much more active than their alkanoyl analogs. Substitution of the phenyl ring strongly influenced the activity depending on the substituent and its ring position (Scheme 6). The most promising derivative **13** with a 3-(trifluoromethyl) group was highly active against the chloroquine-sensitive strain NF54 but even more active against the multiresistant K_1_ strain of *P. falciparum*. Like artemisinin, it possessed activity against this strain in low nanomolar concentration. Compared to the parent compound, it showed improved permeability. Further investigations should reveal if the (3,4-dimethoxyphenyl) substitution is already the optimum in ring position 4 of the furazan ring.

## Figures and Tables

**Table 1:** Activities of compounds **1**, **10**–**17**, **26**–**29**, **31**, **32**, **34**, **35** and **39** against *P. falciparum* NF54, *P. falciparum* K_1_ and L-6 cells, expressed as IC_50_ (µM) ^a^.

Compound	*P.f.* NF54 ^b^ IC_50_ (µM)	*P.f.* K_1_^c^ IC_50_ (µM)	S.I.= IC_50_ (Cyt.)/IC_50_ (*P.f.* NF54)	S.I. = IC_50_ (Cyt.)/IC_50_ (*P.f.* K_1_)	Cytotoxicity L-6 Cells IC_50_ (µM)
**1**	0.011	0.011	14483	14483	159.3
**10**	0.076	0.091	1463	1222	111.2
**11**	0.343		43.49		14.92
**12**	0.831		17.13		14.24
**13**	0.019	0.007	524.6	1424	9.97
**14**	0.049	0.108	838.6	380.5	41.09
**15**	0.098		142.8		13.99
**16**	0.014		1431		20.03
**17**	0.167		209.8		35.03
**26**	4.055	1.87	25.07	54.25	101.7
**27**	0.674	0.586	172.7	199.7	116.4
**28**	0.192		402.2		77.23
**29**	0.712		146.0		104.0
**31**	7.24	5.02	33.46	48.25	242.3
**32**	24.25	19.53	10.95	13.61	265.7
**34**	56.01	2.41	2.50	58.09	139.8
**35**	30.15	18.99	4.88	7.76	147.3
**39**	21.52	39.92	3.07	1.65	65.95
**ART**		0.0064		70390	450.5
**CQ**		0.15		1570	188.5
**POD**					0.012

ART = artemisinin; CQ = chloroquine; POD = podophyllotoxin. ^a^ Values represent the average of four determinations (two determinations of two independent experiments); ^b^ sensitive to chloroquine; ^c^ resistant to chloroquine and pyrimethamine.

**Table 2:** Key physicochemical parameters and PAMPA values of compounds **1**, **10**–**17**, **26**–**29**, **31**, **32**, **34**, **35** and **39.**

Compound	log P ^a^	log D_7.4_^a^	LE (kcal/mol/HA)	Pe ^b^ (10^−6^ cm/s)
**1**	4.19	4.19	0.404	2.77
**10**	3.68	3.68	0.375	n.d.
**11**	4.19	4.19	0.328	n.d.
**12**	4.56	4.56	0.278	n.d.
**13**	4.56	4.56	0.353	4.26
**14**	3.82	3.82	0.371	n.d.
**15**	5.11	5.11	0.310	n.d.
**16**	4.13	4.13	0.371	n.d.
**17**	3.66	3.66	0.320	3.90
**26**	3.90	2.47	0.231	8.13
**27**	3.40	3.34	0.256	n.d.
**28**	4.03	3.09	0.288	7.26
**29**	3.40	3.39	0.255	5.65
**31**	2.12	1.83	0.271	10.80
**32**	1.50	1.38	0.234	10.25
**34**	2.36	0.57	0.216	6.93
**35**	1.74	1.53	0.221	6.08
**39**	3.79	3.79	0.287	8.47

^a^ Log P and log D were calculated using the ChemAxon software JChem for Excel 14.9.1500.912 (2014); ^b^ determined by PAMPA; n.d., could not be determined.

## Data Availability

The data presented in this study are available in this article.

## Supplementary Materials

The following materials are available online at https://www.mdpi.com/article/10.3390/ph14050412/s1. Figures S1–S23. 1H- and 13C-NMR spectra for compounds **6**, **2**, **8**, **9**, **20**, **1**, **10**–**17**, **26**–**29**, **31**, **32**, **34**, **35** and **39**. Figures S24–S41. Dose-response curves of compounds **1**, **10**–**17**, **26**–**29**, **31**, **32**, **34**, **35** and **39** against *P.f.* NF54, *P.f.* K_1_ and L-6 cells. Table S1**.** Activities with standard deviation values of compounds **1**, **10**–**17, 26**–**29**, **31**, **32**, **34**, **35** and **39** against *P. falciparum* NF54, *P. falciparum* K_1_ and L-6 cells, expressed as IC_50_ (µM).
