# Supplementary material for: New Acyl Derivatives of 3-Aminofurazanes and Their Antiplasmodial Activities
Source: Pharmaceuticals (Basel). 2021 Apr 27;14(5):412. doi: 10.3390/ph14050412 (PMC8145535; doi:10.3390/ph14050412)
Supplement: Supplementary file 1 [file pharmaceuticals-14-00412-s001.zip › pharmaceuticals-1182101-supplementary.pdf]

Supplementary Information

New acyl derivatives of 3-aminofurazanes and their antiplasmodial activities

Theresa Hermann [1], Patrick Hochegger [1,*], Johanna Dolensky [1], Werner Seebacher [1], Robert Saf [2], Marcel Kaiser [3], Pascal Mäser [3] and Robert Weis [1]

[1] Institute of Pharmaceutical Sciences, Pharmaceutical Chemistry, University of Graz, Schubertstraße 1, A-8010 Graz, Austria; theresa.hermann@uni-graz.at; patrick.hochegger@uni-graz.at; we.seebacher@uni-graz.at; robert.weis@uni-graz.at
[2] Institute for Chemistry and Technology of Materials (ICTM), Graz University of Technology, Stremayrgasse 9, A-8010 Graz, Austria; robert.saf@tugraz.at
[3] Swiss Tropical and Public Health Institute, Socinstraße 57, CH-4002 Basel, Switzerland; marcel.kaiser@swisstph.ch; pascal.maeser@swisstph.ch
* Correspondence: patrick.hochegger@uni-graz.at; Tel.: +43-316-380-5379; fax: +43-316-380-9846

NMR spectra data of compounds 6, 2, 8, 9, 20, 1, 10-17, 26-29, 31, 32, 34, 35 and 39

Figure S1. 1H NMR at 400 MHz and 13C NMR at 100 MHz spectra for compound **6**

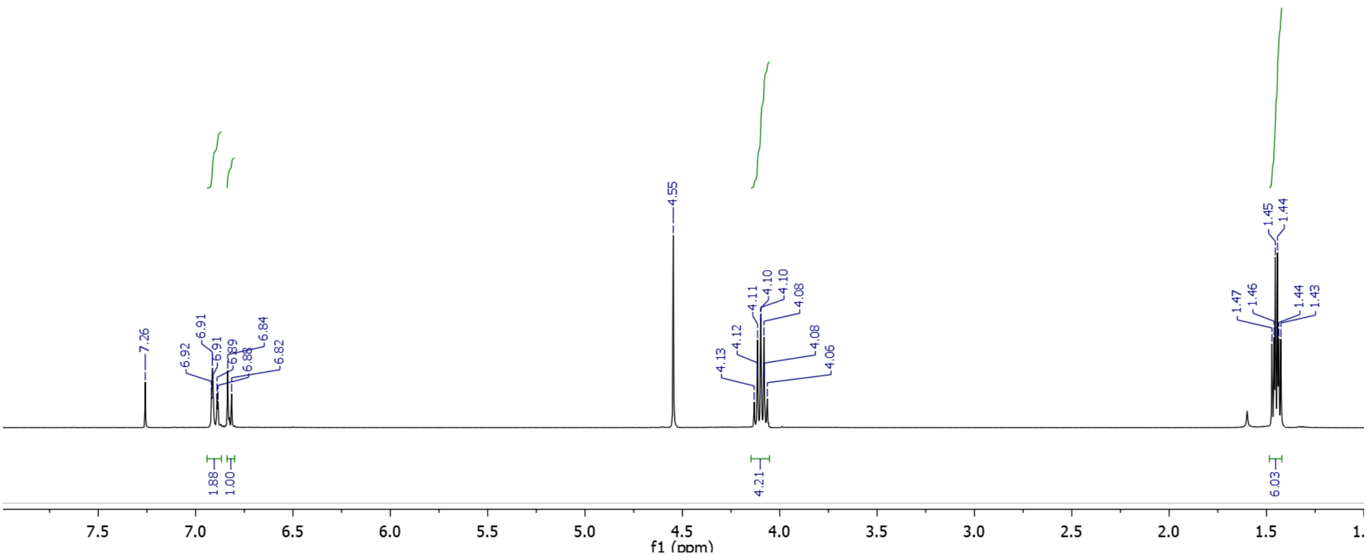

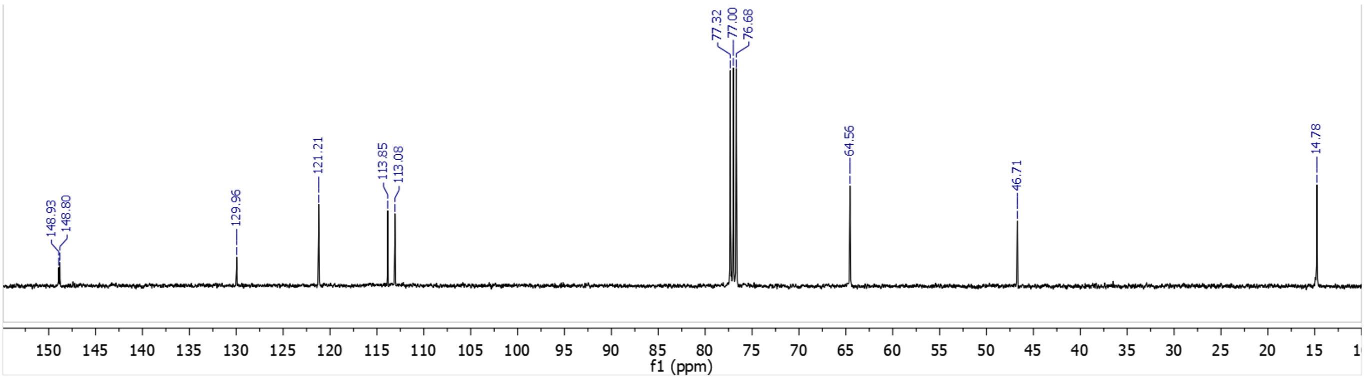

Figure S2. 1H NMR at 400 MHz and 13C NMR at 100 MHz spectra for compound **2**

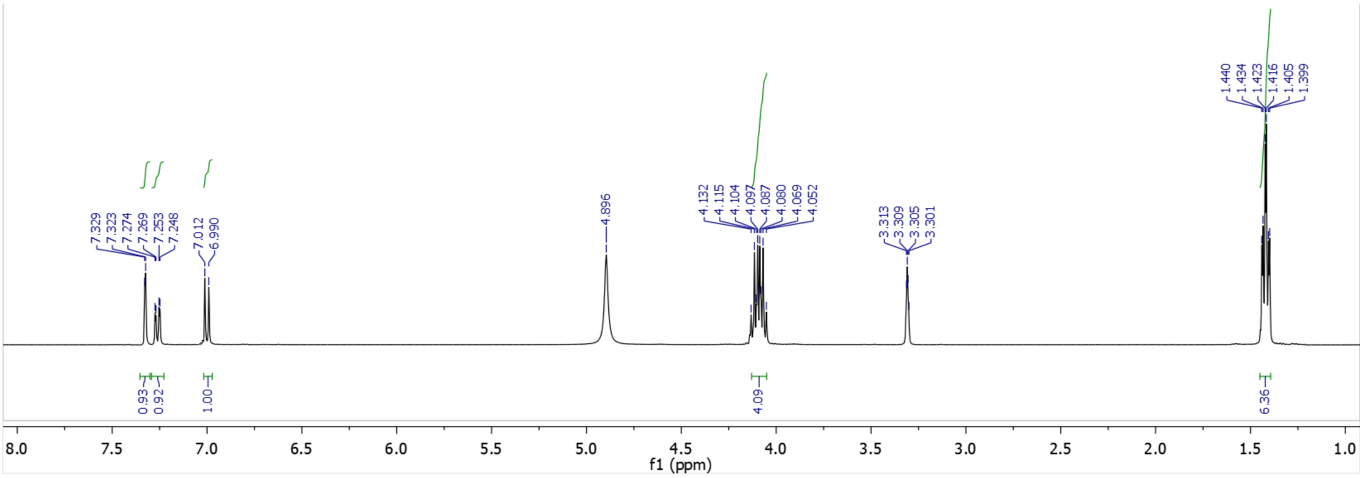

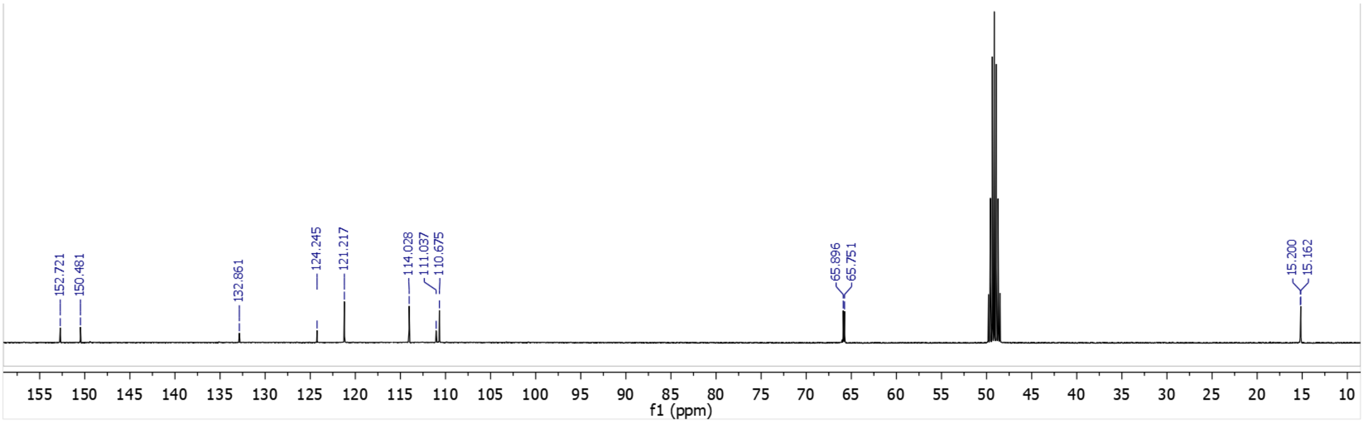

Figure S3. 1H NMR at 400 MHz and 13C NMR at 100 MHz spectra for compound **8**

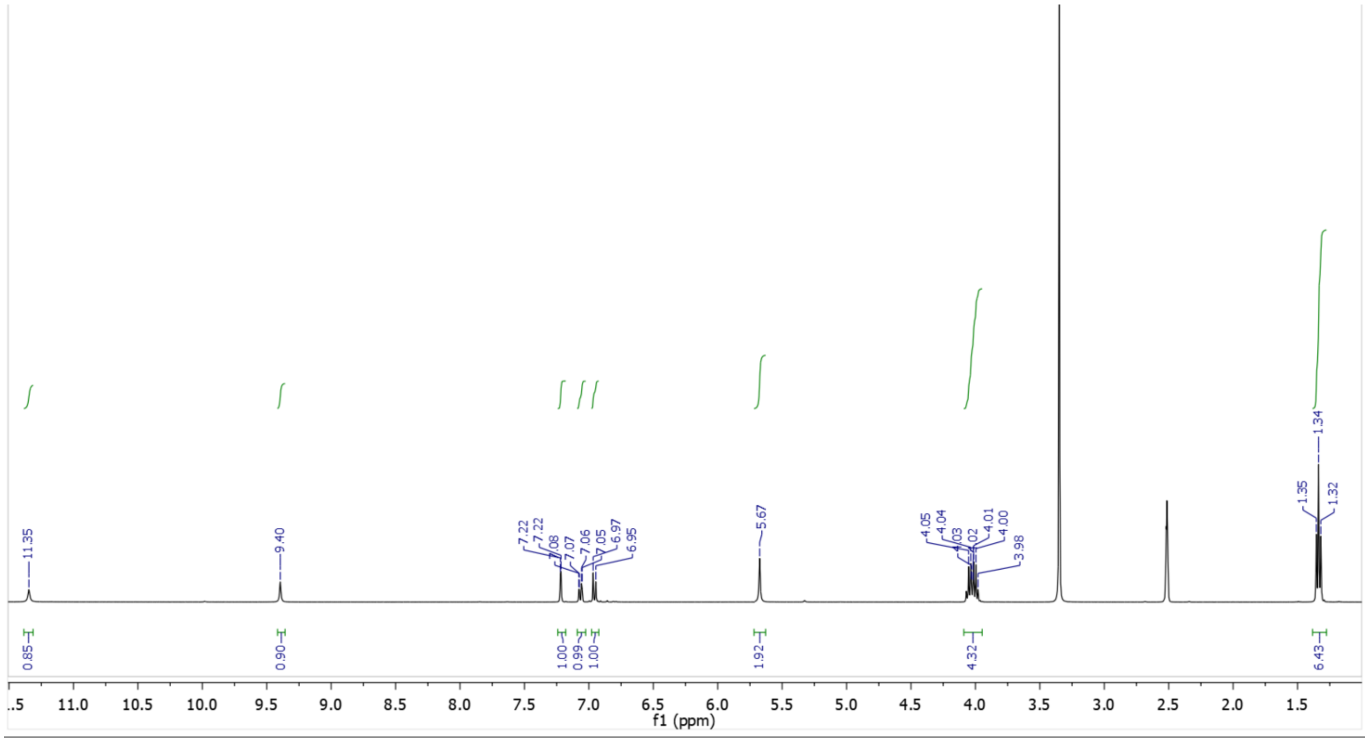

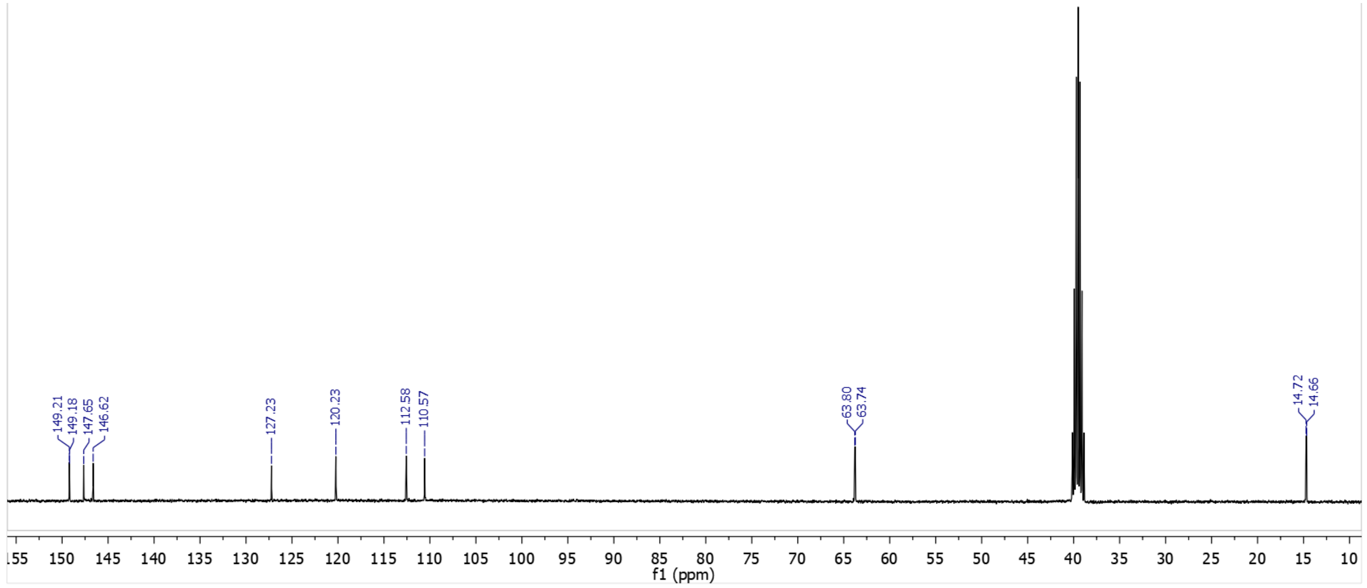

Figure S4. 1H NMR at 400 MHz and 13C NMR at 100 MHz spectra for compound **9**

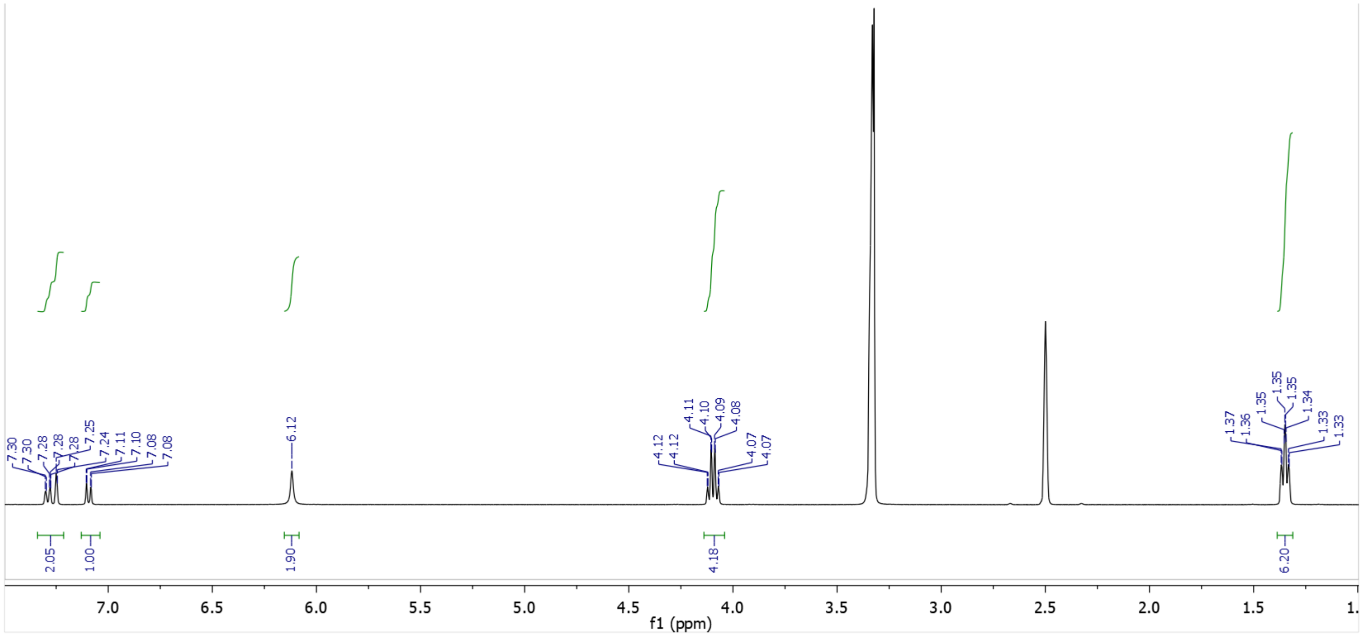

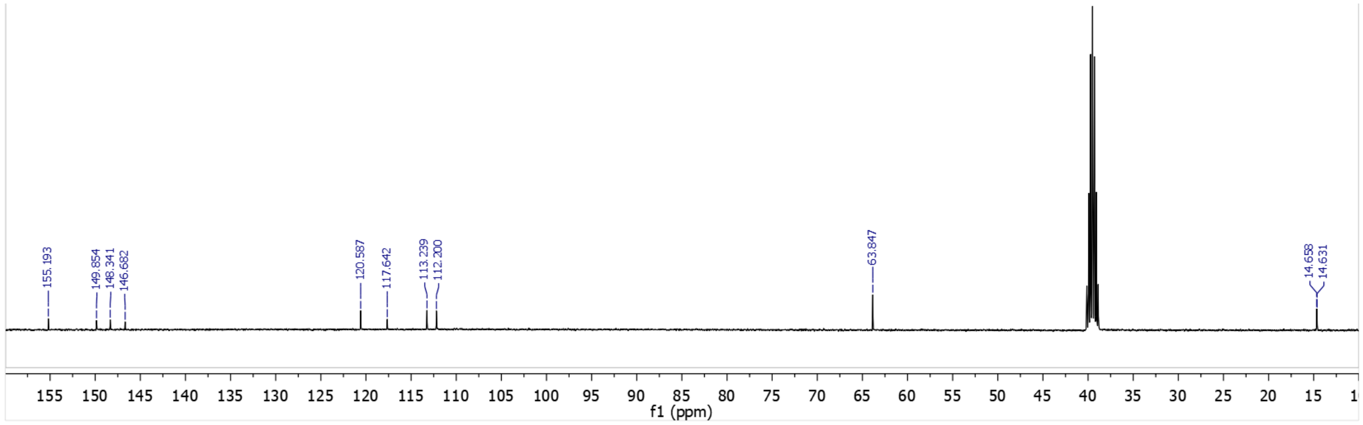

Figure S5. 1H NMR at 400 MHz and 13C NMR at 100 MHz spectra for compound **20**

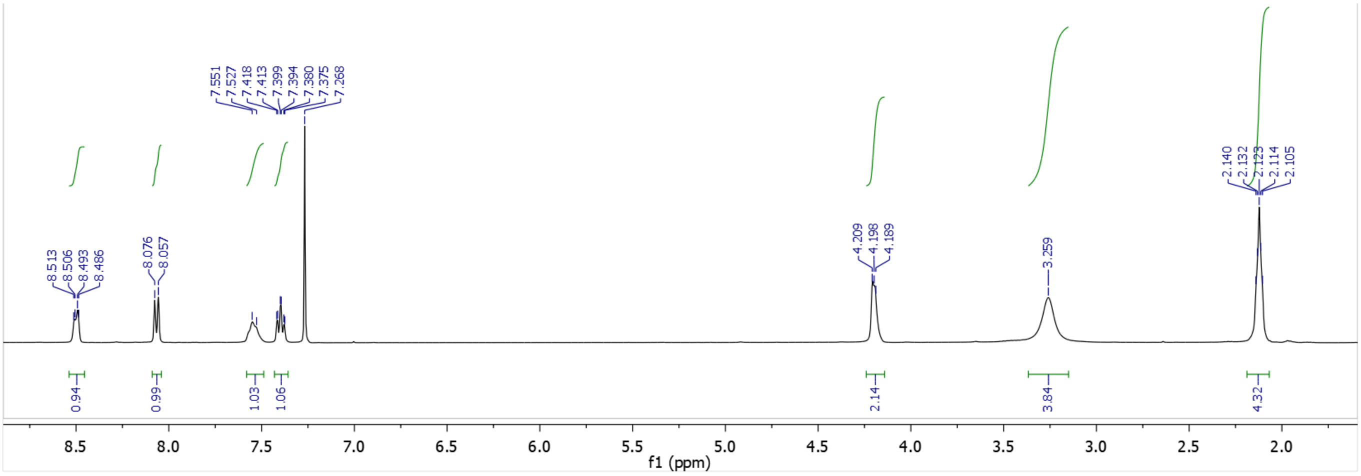

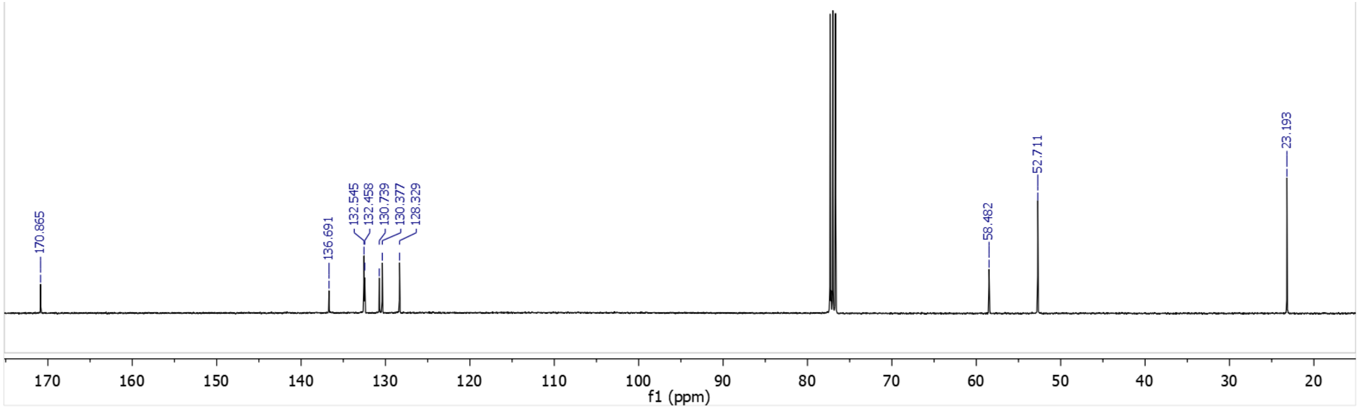

Figure S6. 1H NMR at 400 MHz and 13C NMR at 100 MHz spectra for compound **1**

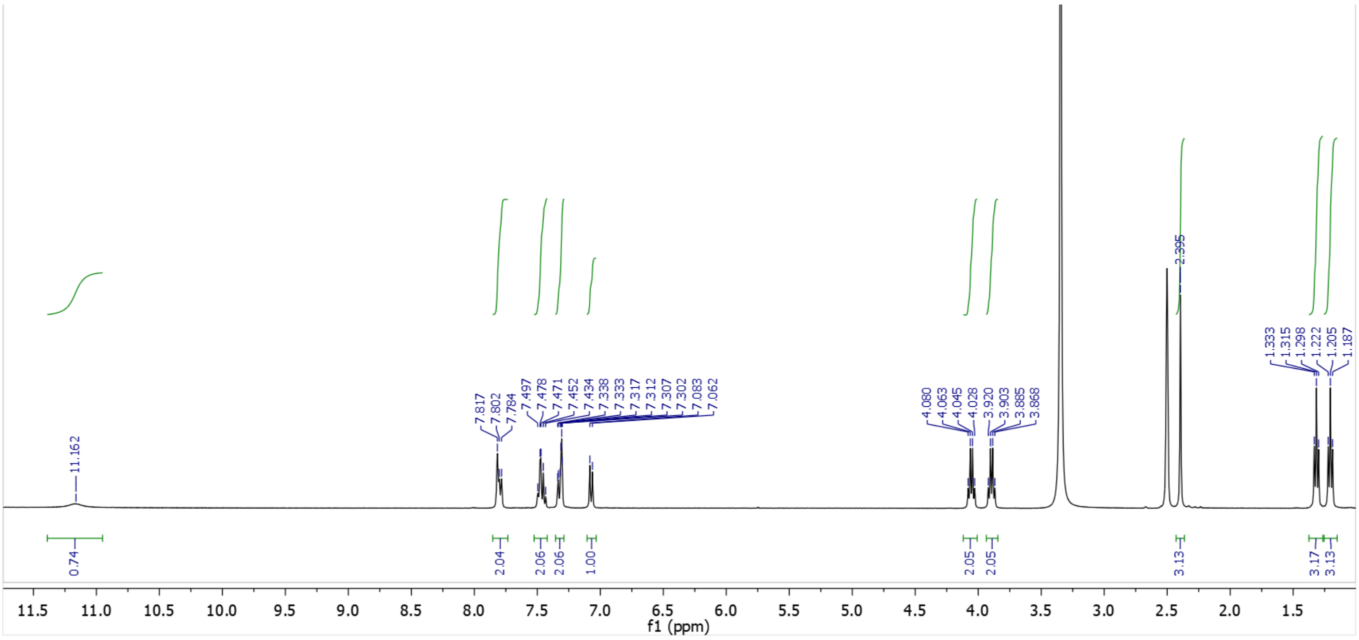

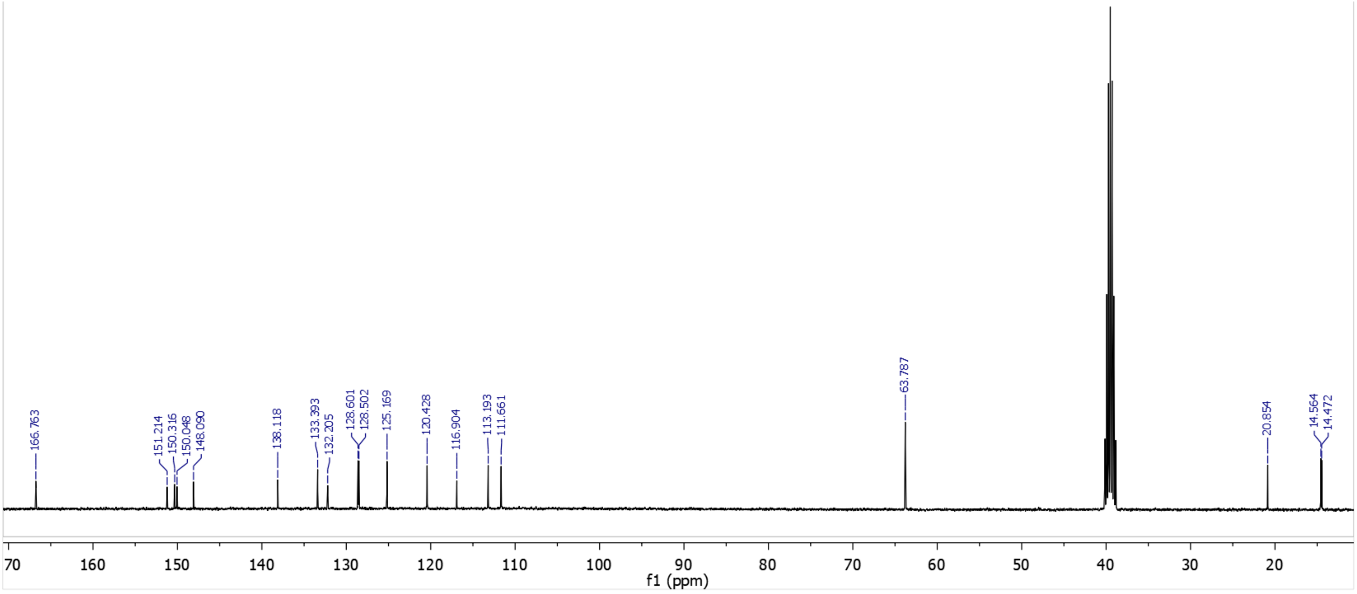

Figure S7. 1H NMR at 400 MHz and 13C NMR at 100 MHz spectra for compound **10**

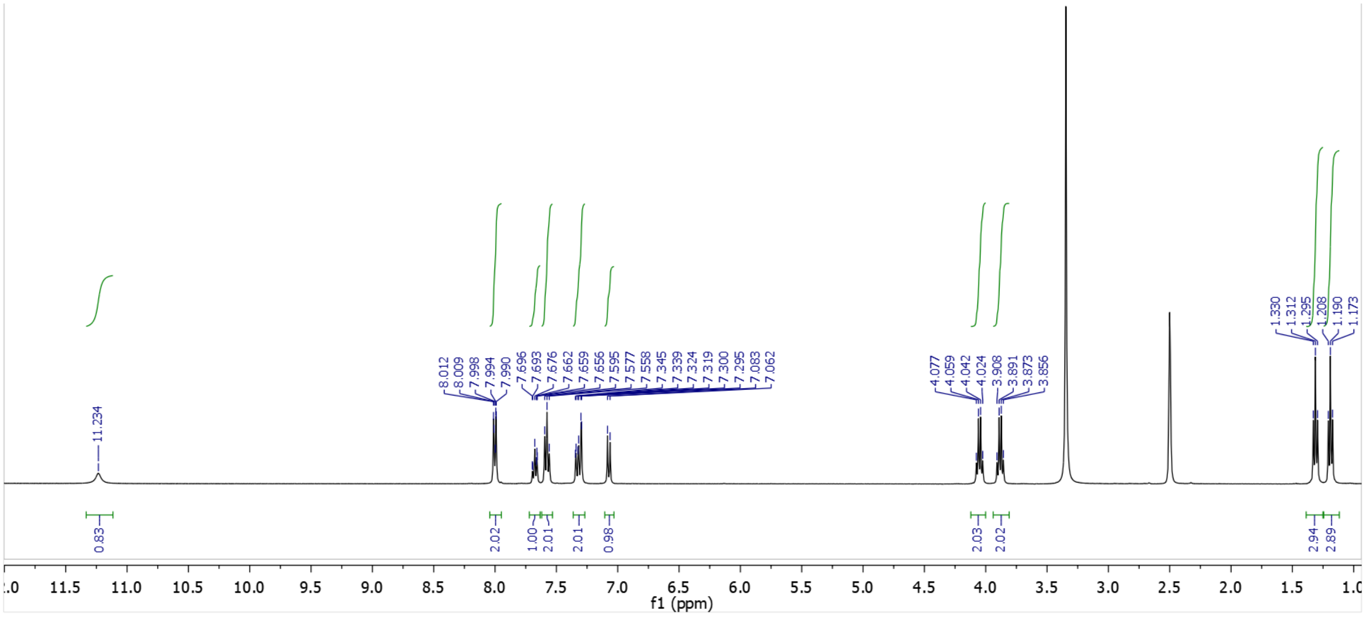

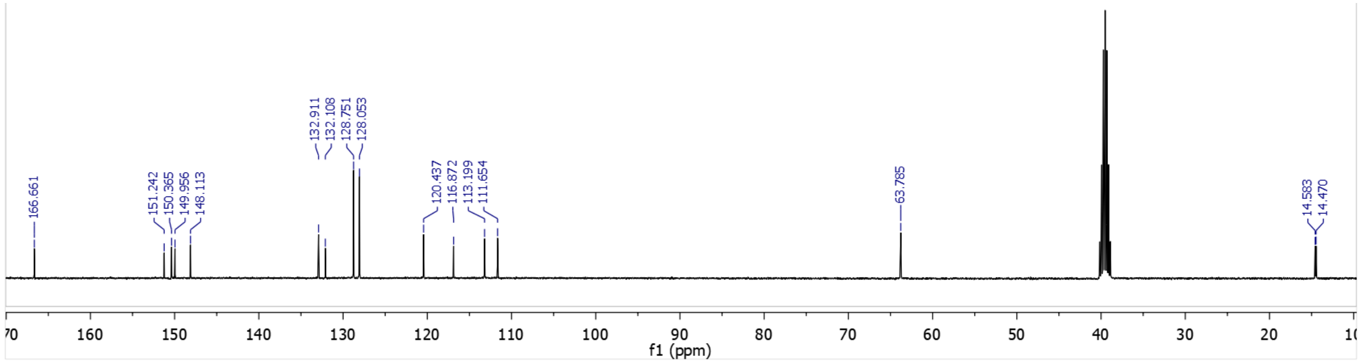

Figure S8. 1H NMR at 400 MHz and 13C NMR at 100 MHz spectra for compound **11**

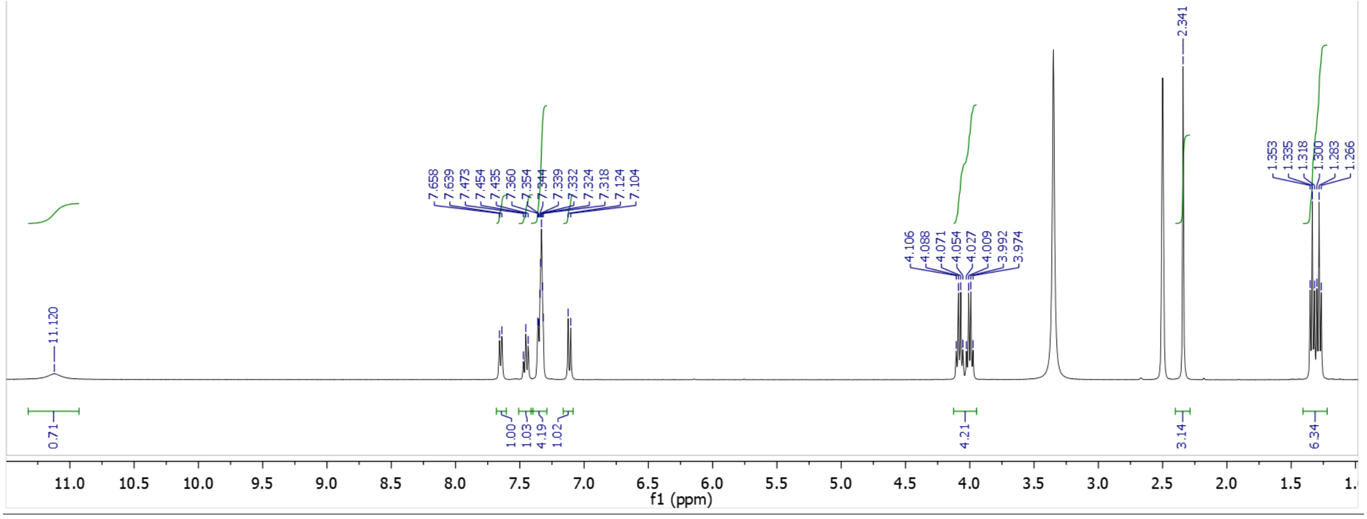

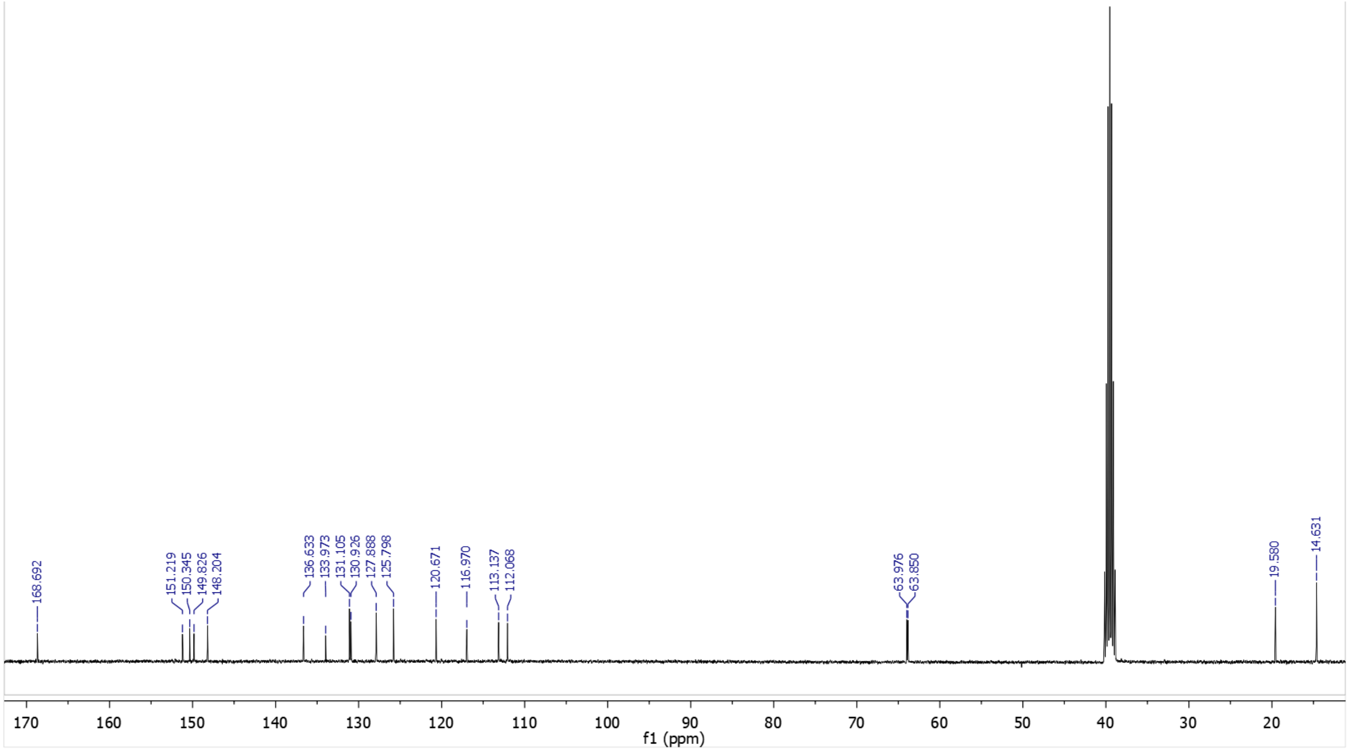

Figure S9. 1H NMR at 400 MHz and 13C NMR at 100 MHz spectra for compound **12**

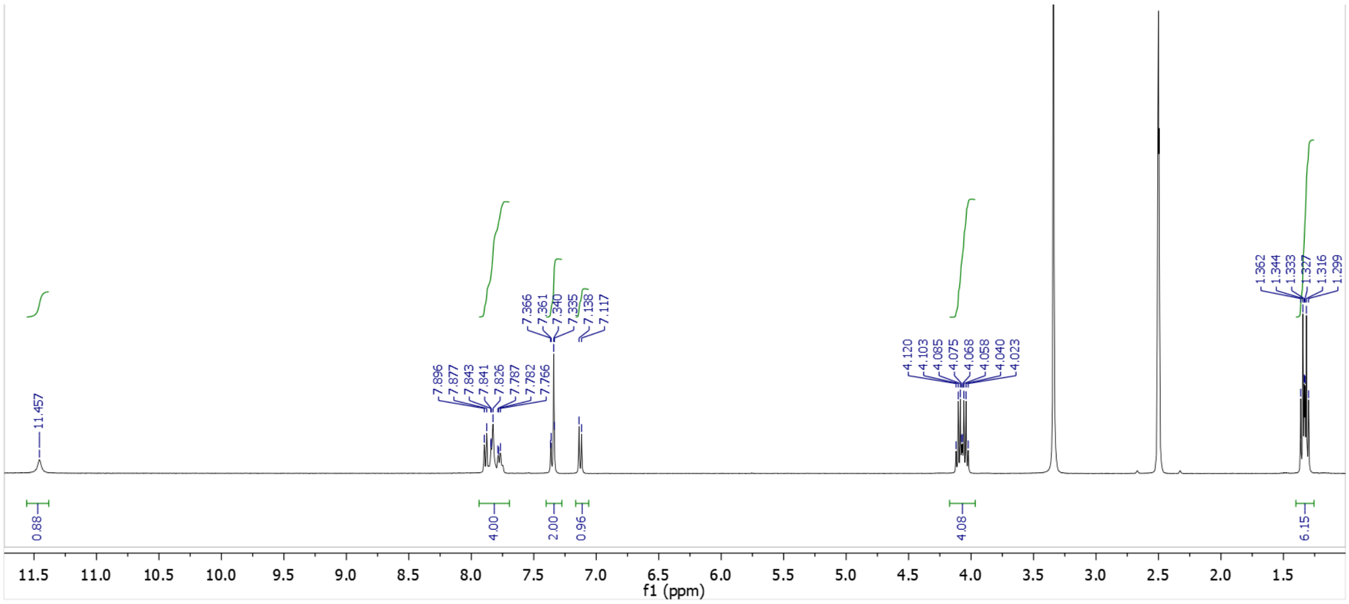

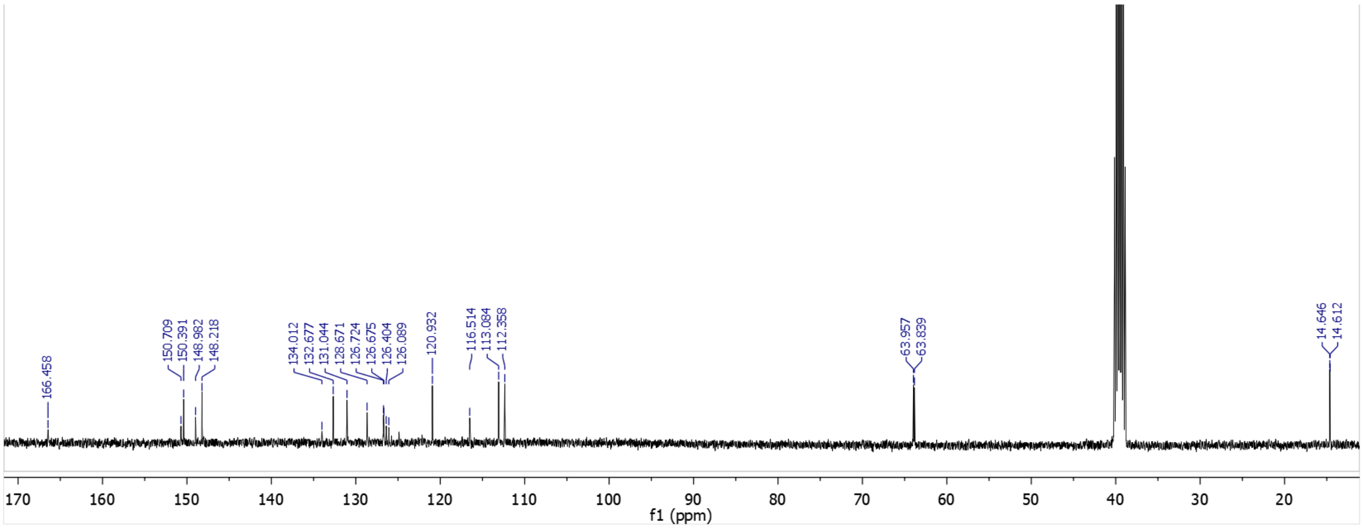

Figure S10. 1H NMR at 400 MHz and 13C NMR at 100 MHz spectra for compound **13**

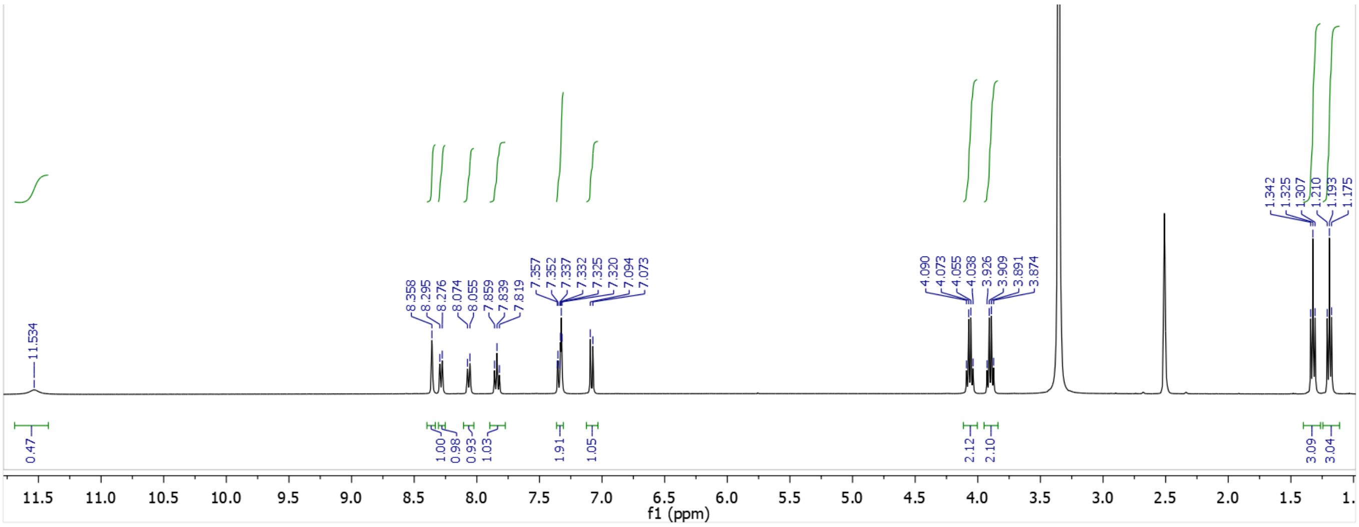

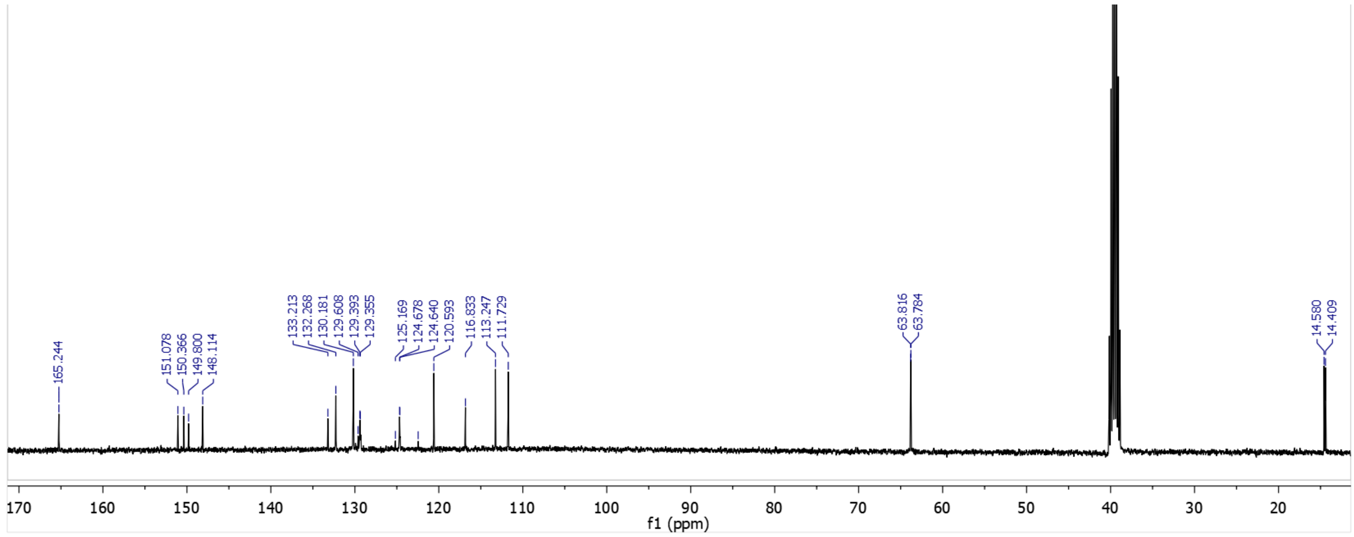

Figure S11. 1H NMR at 400 MHz and 13C NMR at 100 MHz spectra for compound **14**

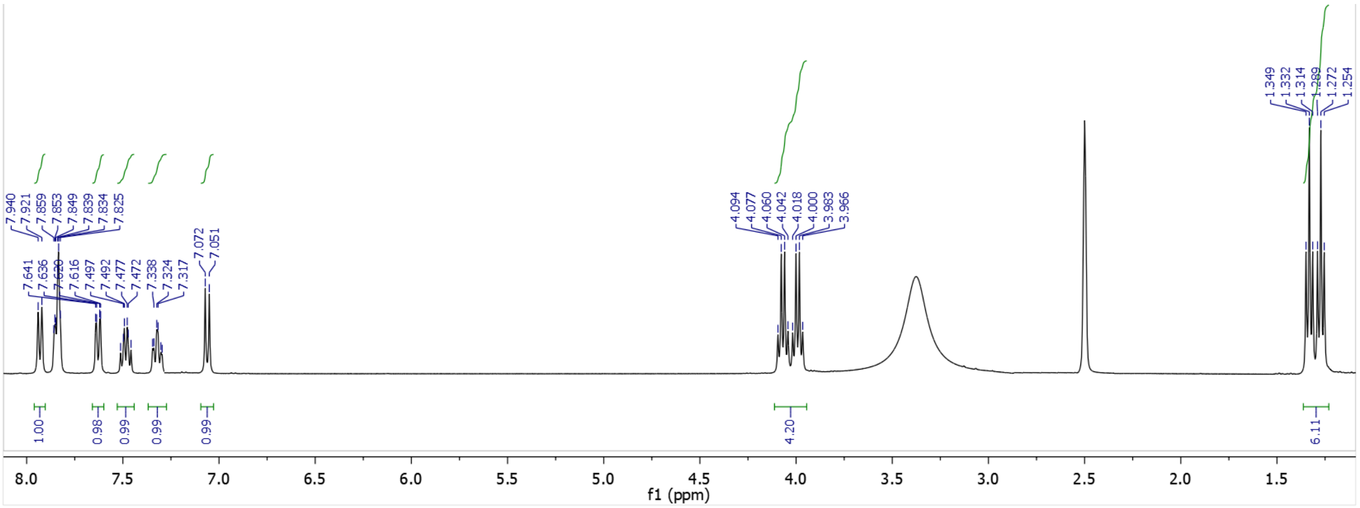

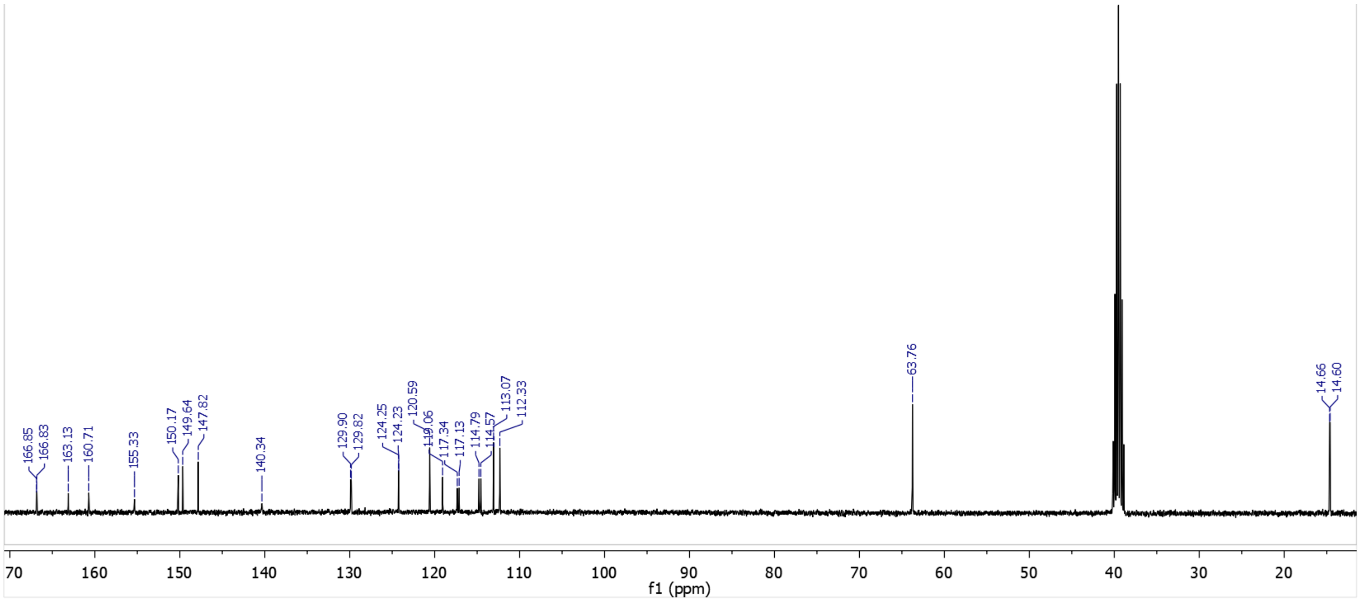

Figure S12. 1H NMR at 400 MHz and 13C NMR at 100 MHz spectra for compound **15**

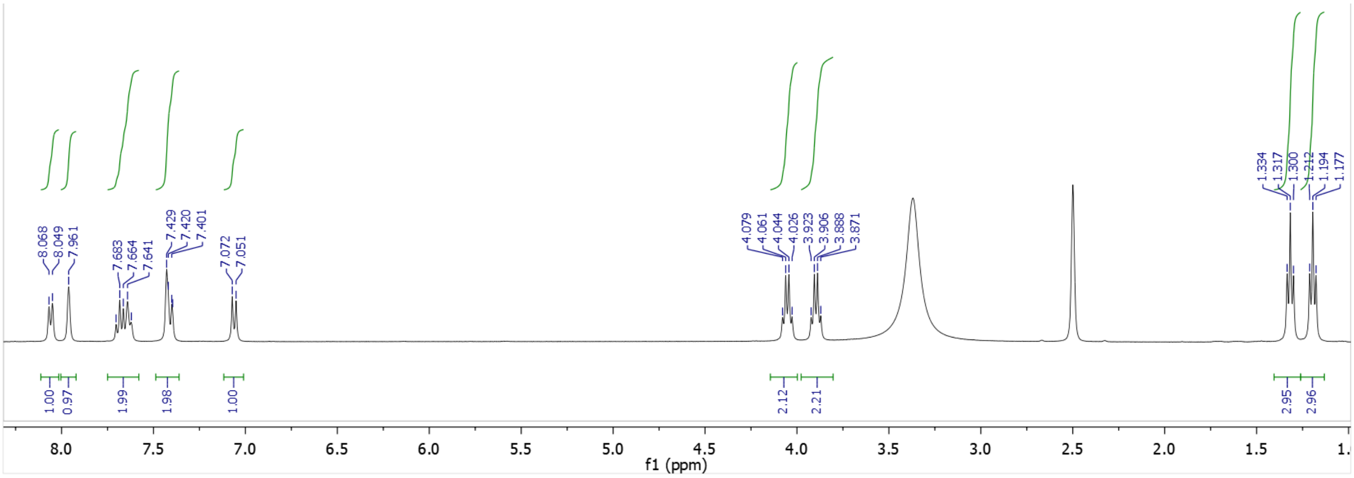

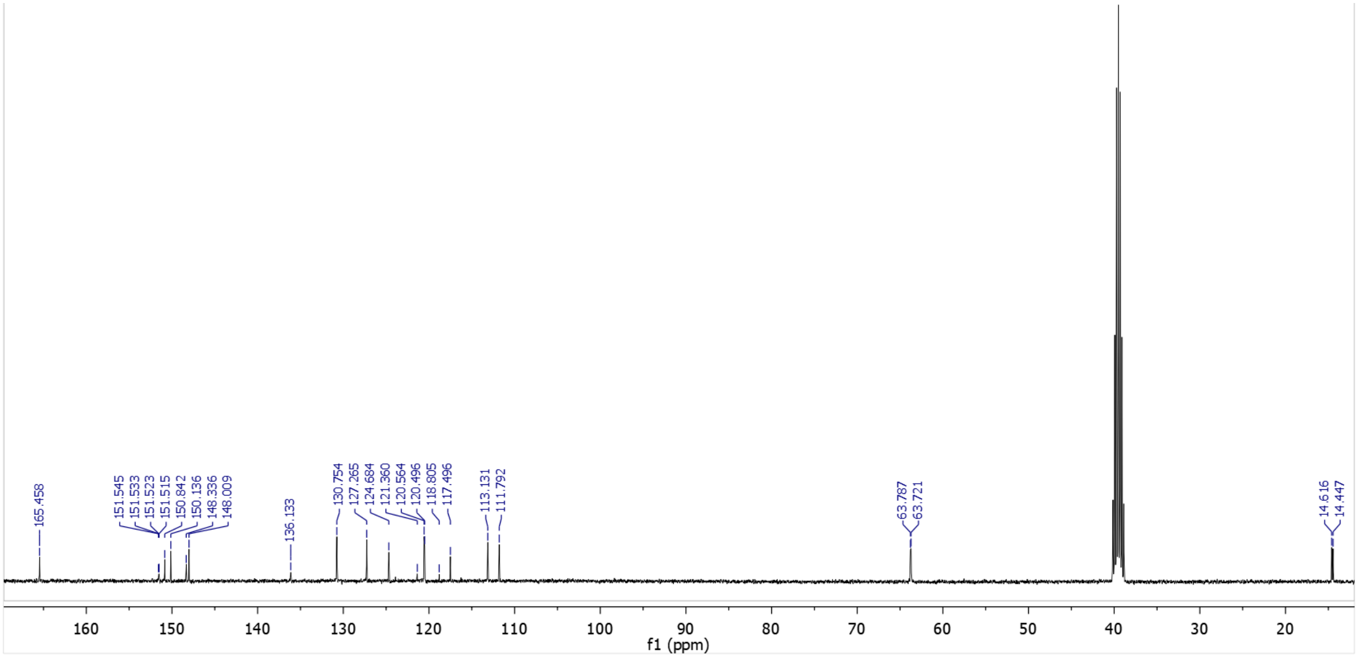

Figure S13. 1H NMR at 400 MHz and 13C NMR at 100 MHz spectra for compound 1**6**

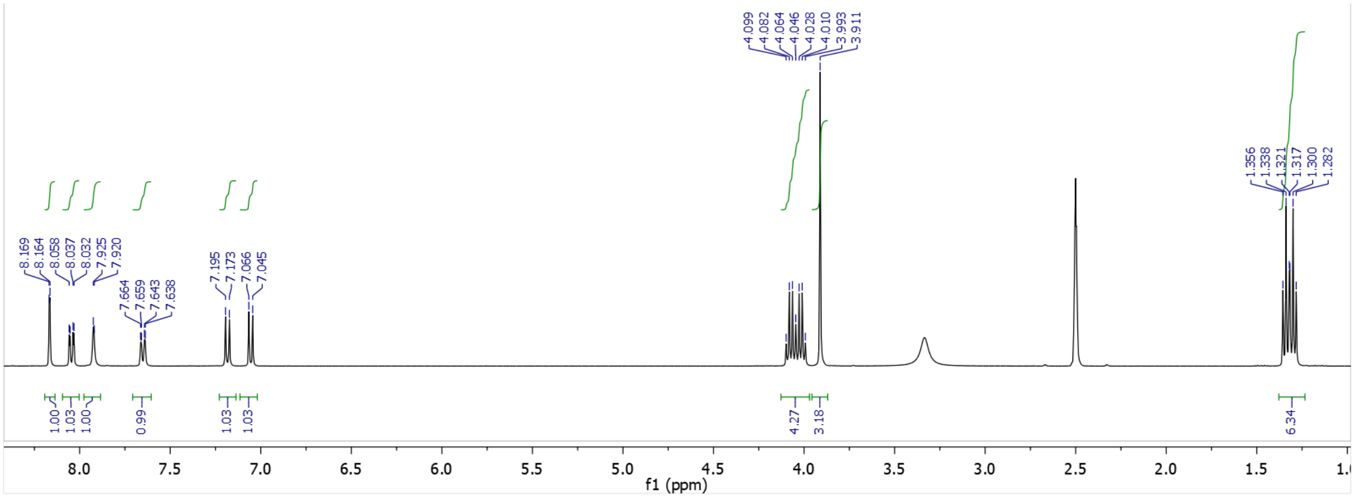

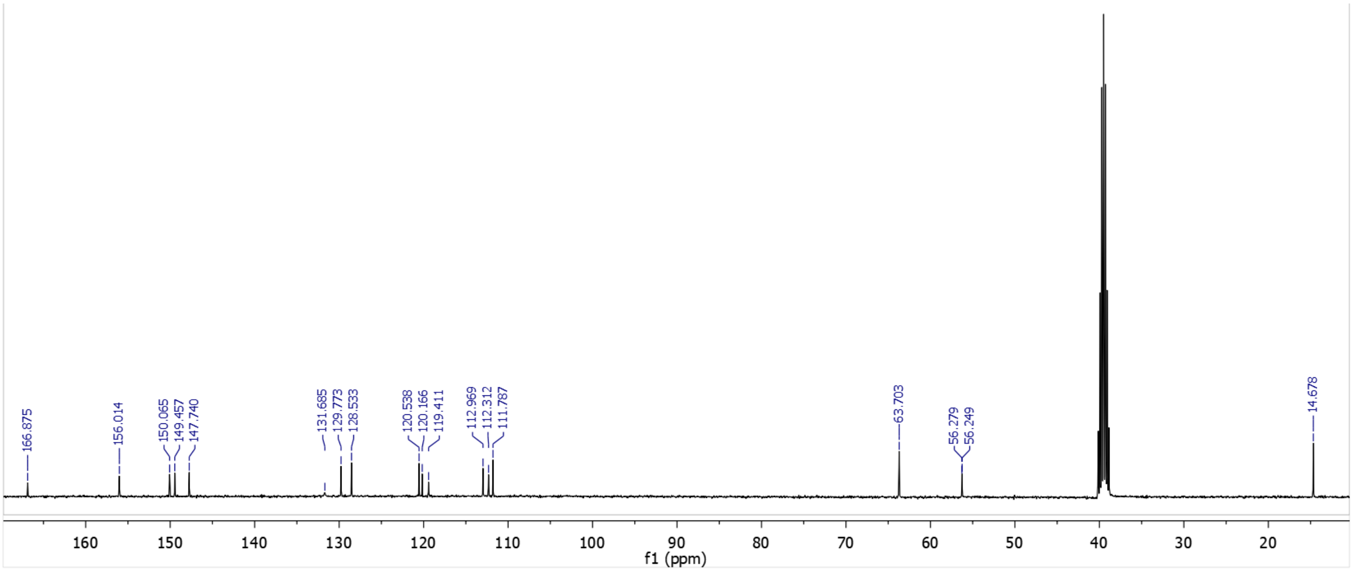

Figure S14. 1H NMR at 400 MHz and 13C NMR at 100 MHz spectra for compound **17**

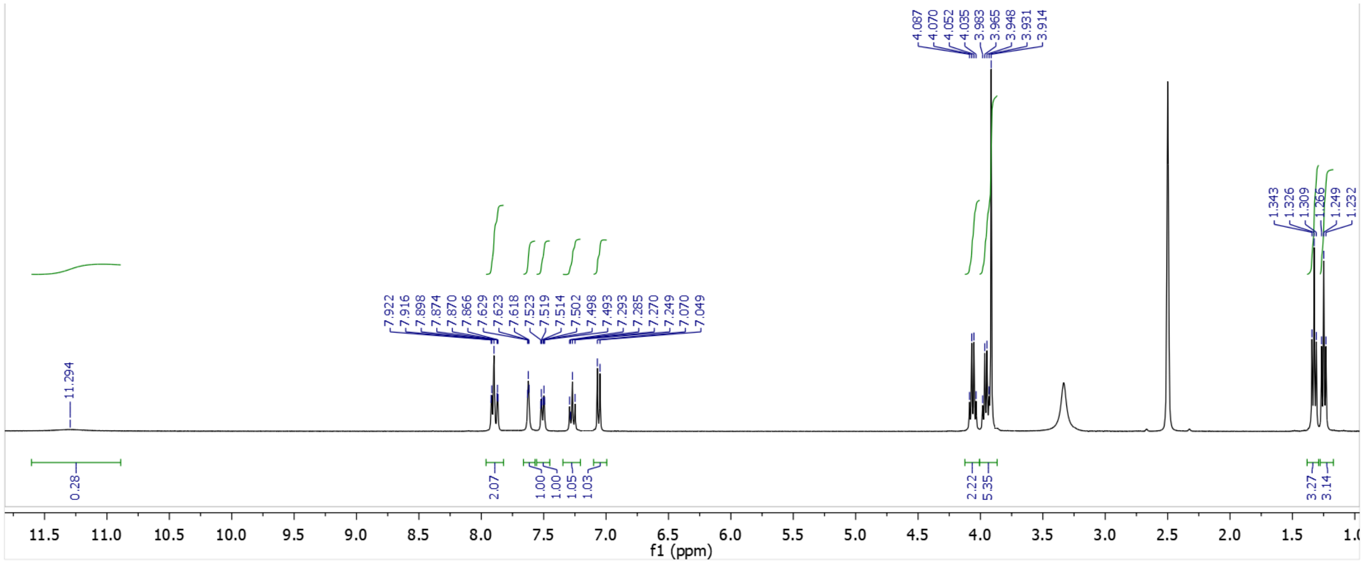

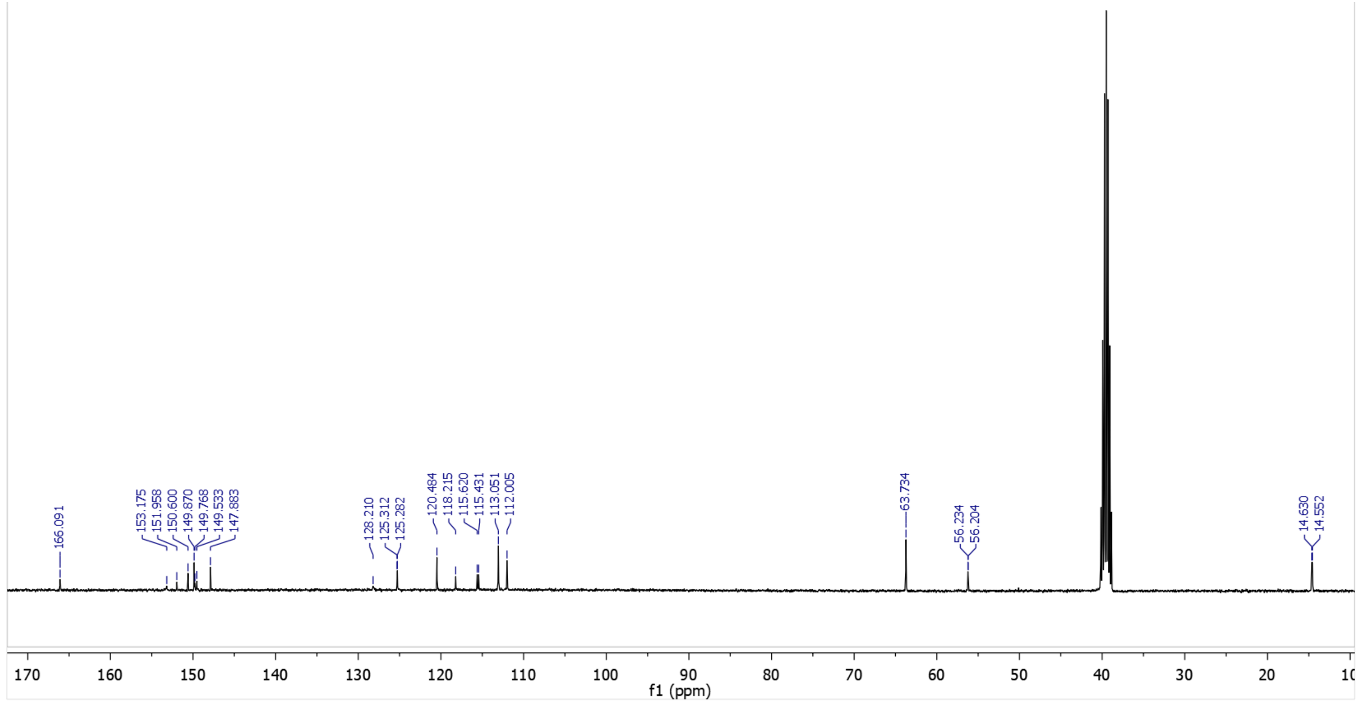

Figure S15. 1H NMR at 400 MHz and 13C NMR at 100 MHz spectra for compound **26**

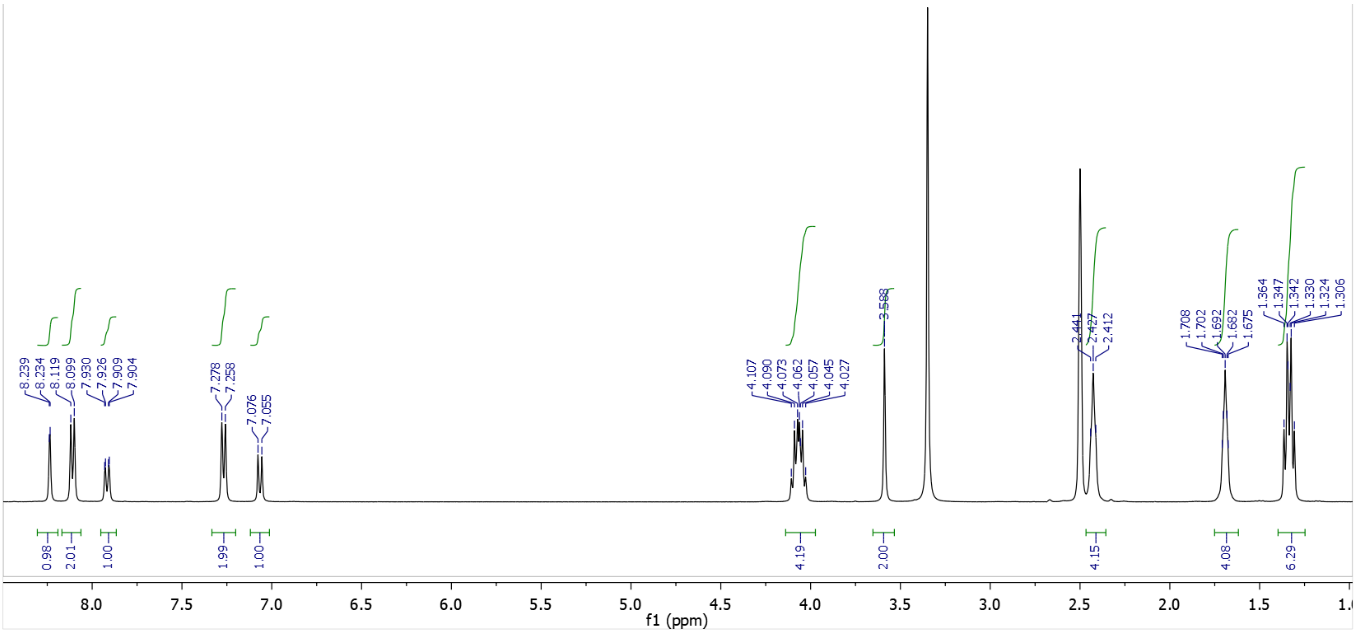

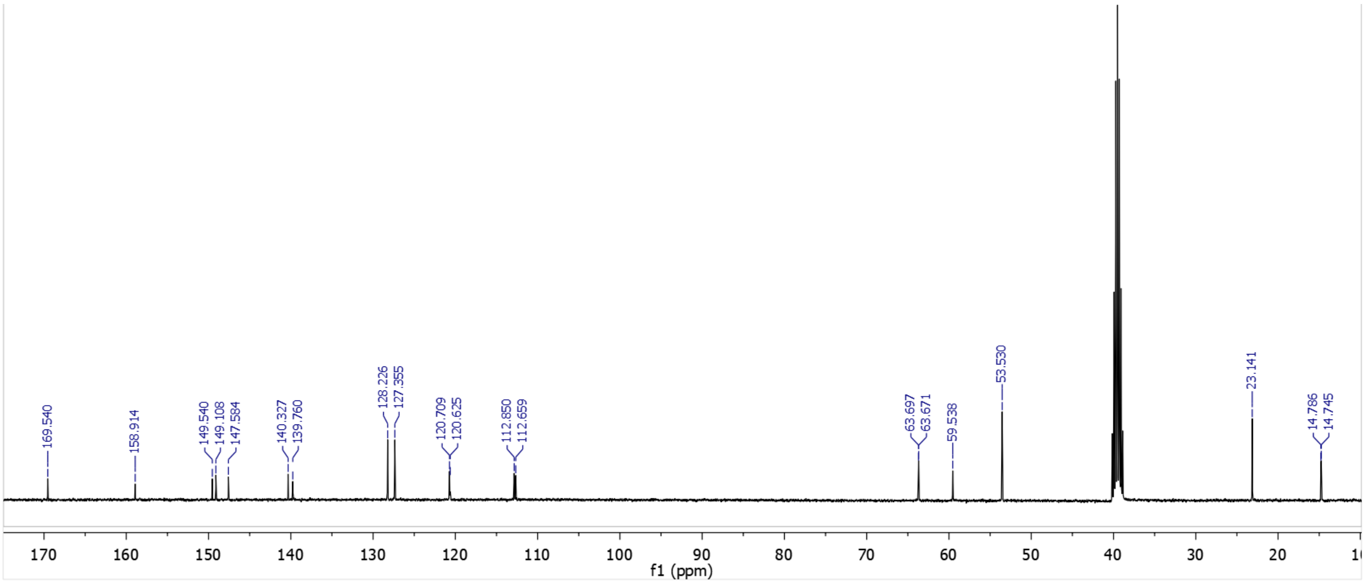

Figure S16. 1H NMR at 400 MHz and 13C NMR at 100 MHz spectra for compound **27**

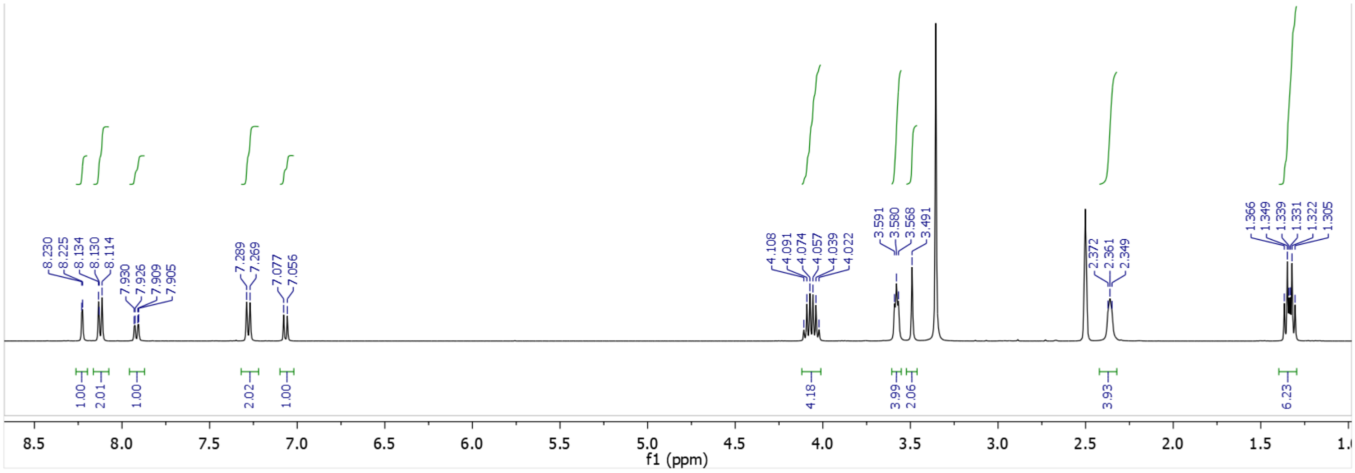

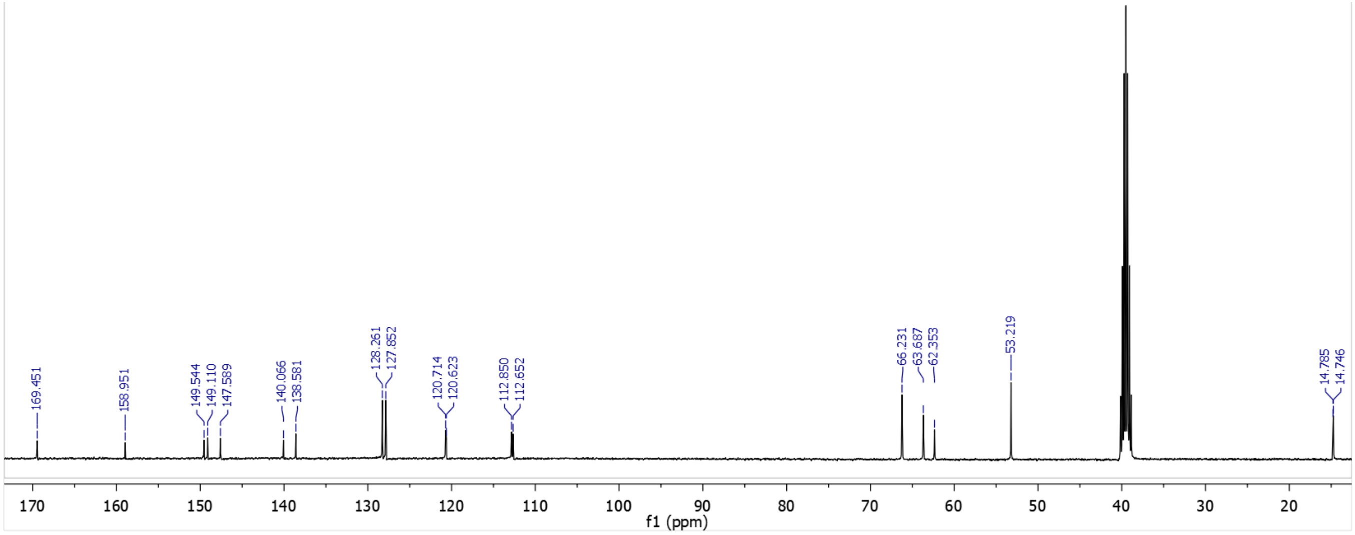

Figure S17. 1H NMR at 400 MHz and 13C NMR at 100 MHz spectra for compound **28**

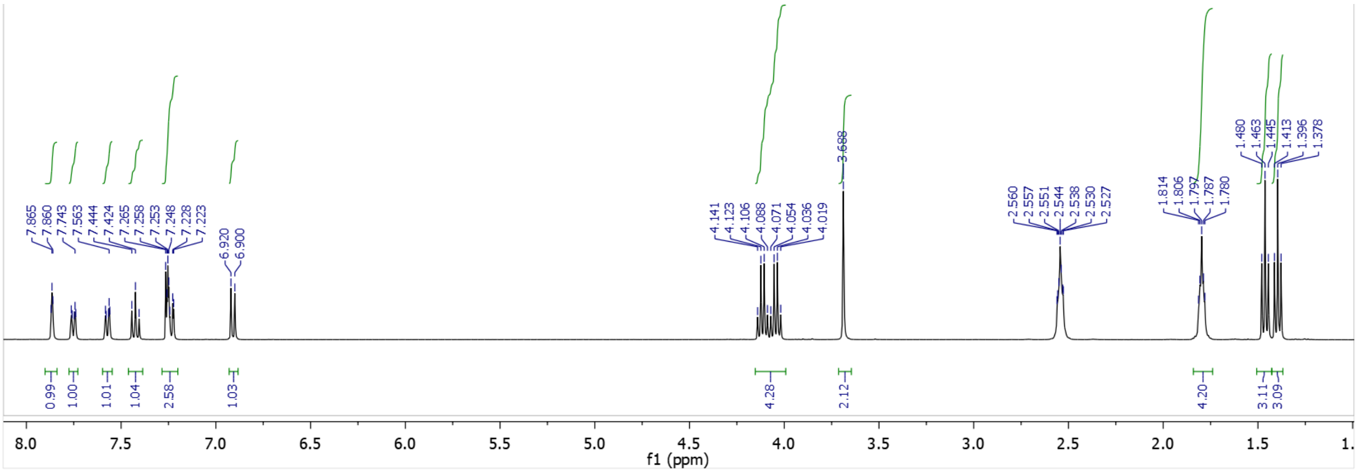

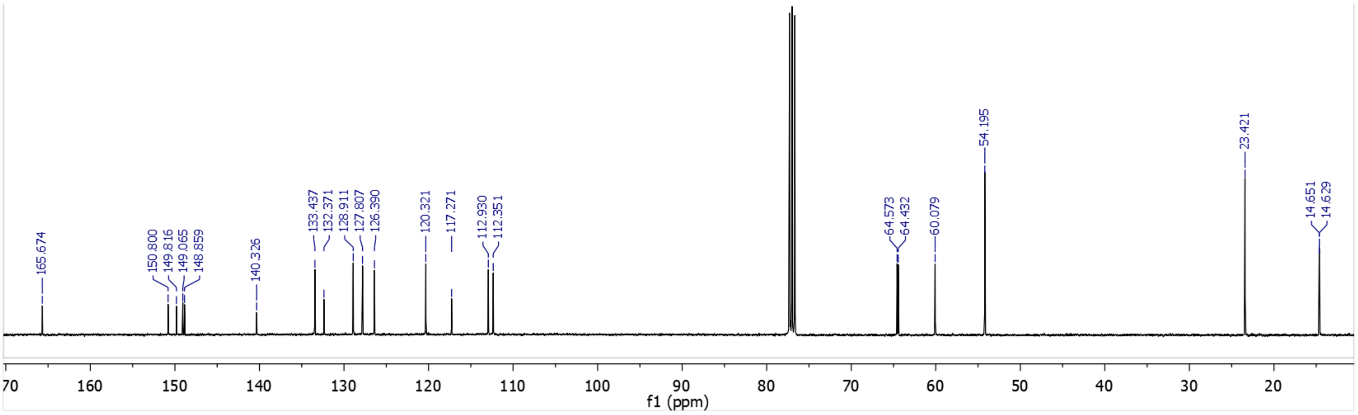

Figure S18. 1H NMR at 400 MHz and 13C NMR at 100 MHz spectra for compound **29**

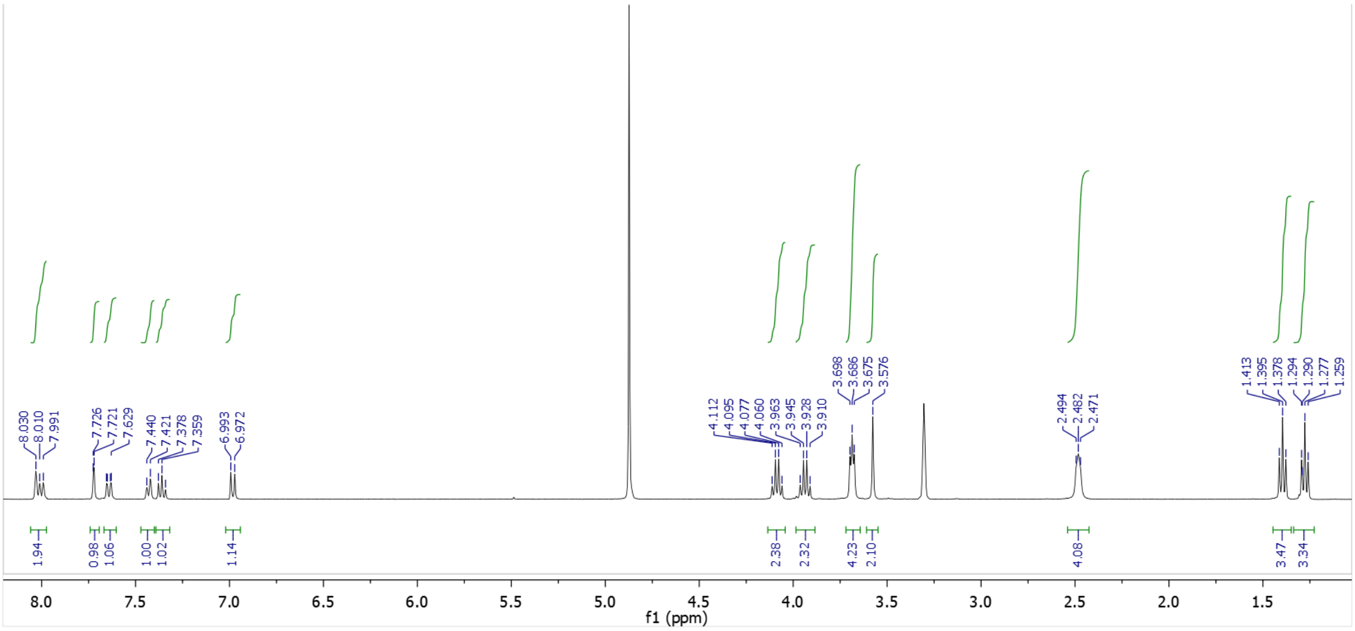

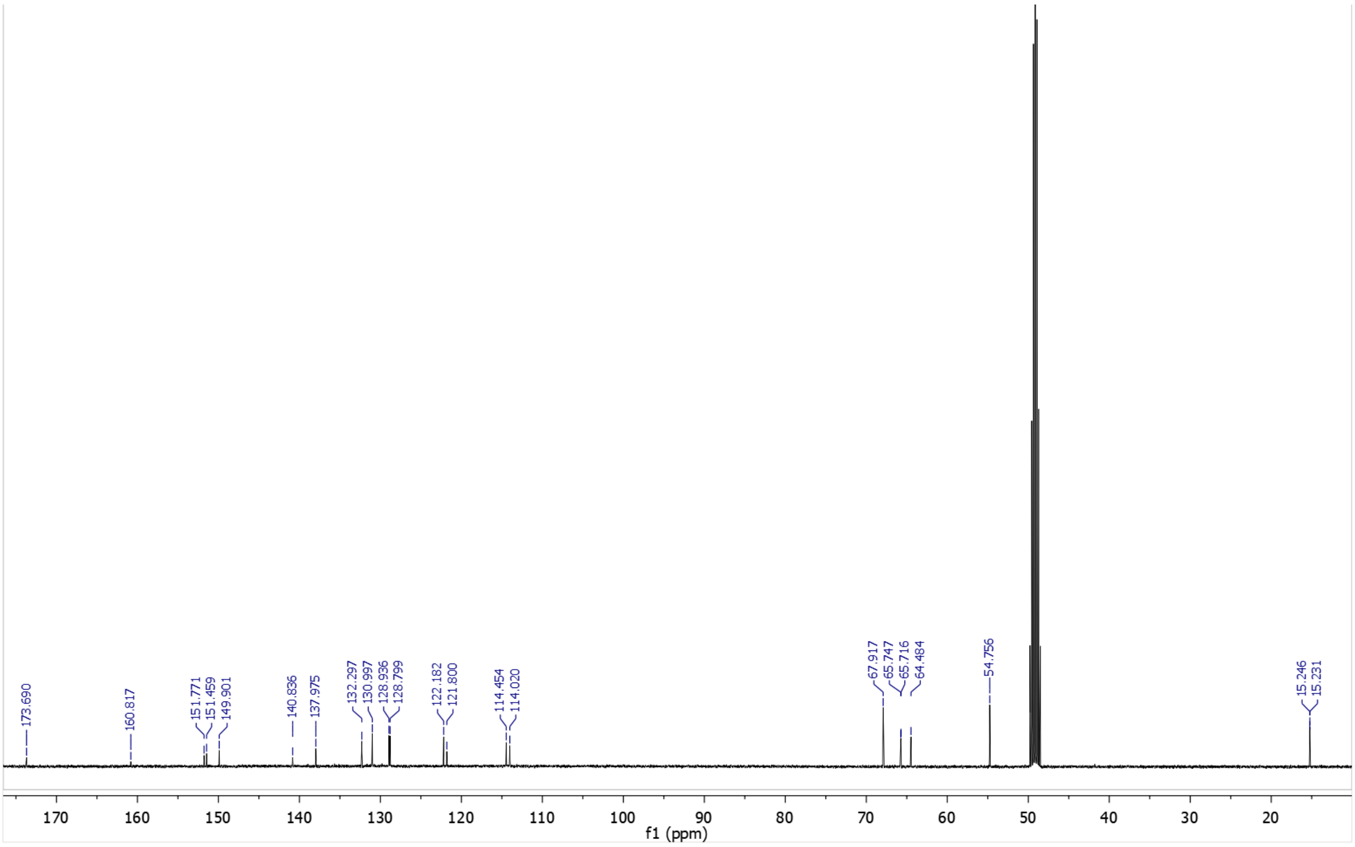

Figure S19. 1H NMR at 400 MHz and 13C NMR at 100 MHz spectra for compound **31**

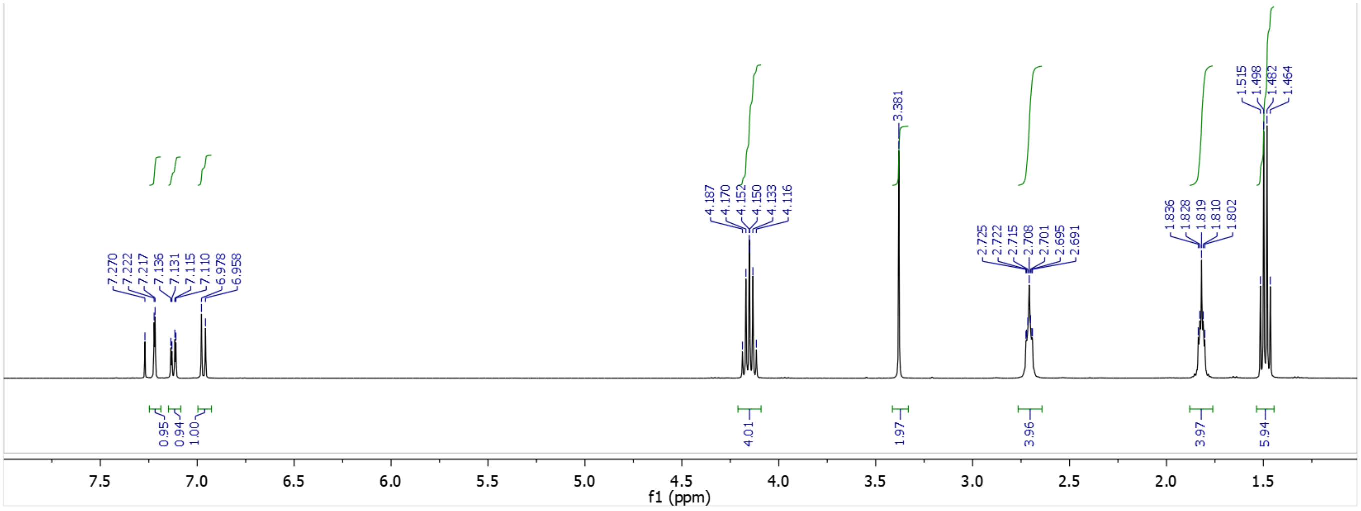

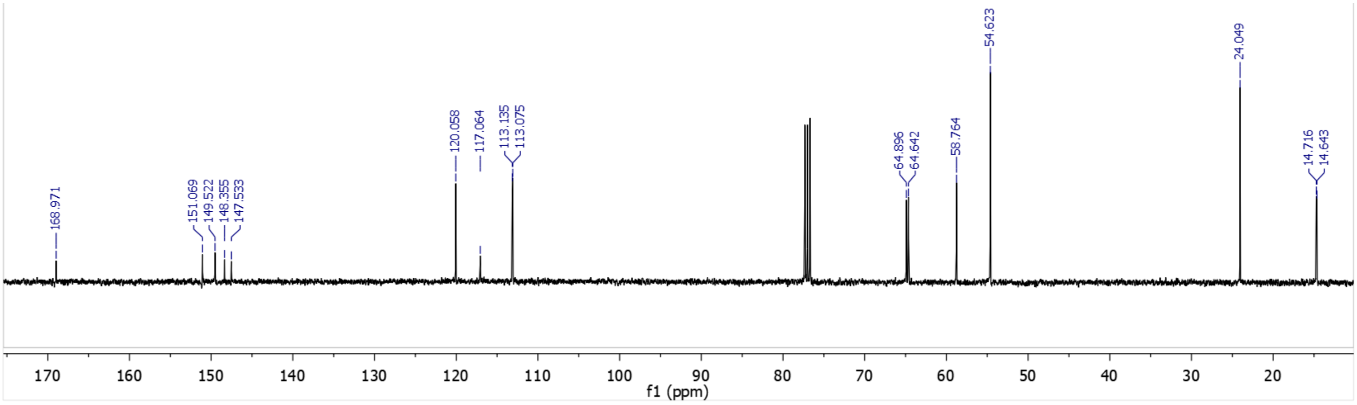

Figure S20. 1H NMR at 400 MHz and 13C NMR at 100 MHz spectra for compound **32**

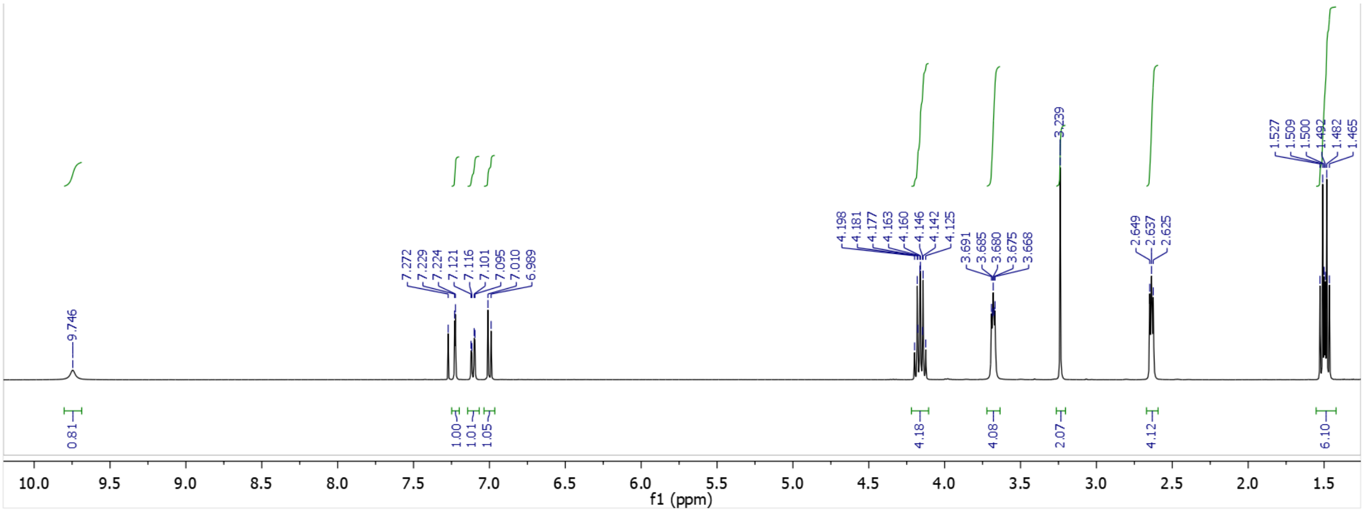

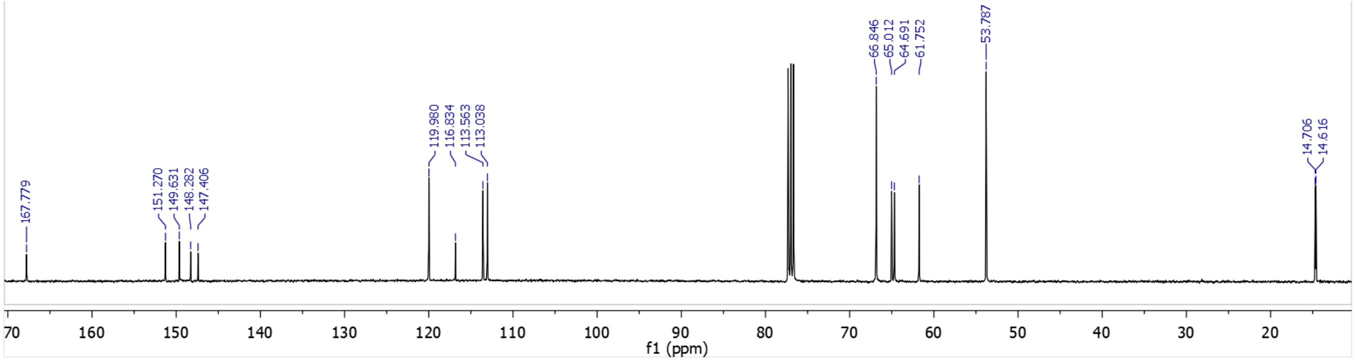

Figure S21. 1H NMR at 400 MHz and 13C NMR at 100 MHz spectra for compound **34**

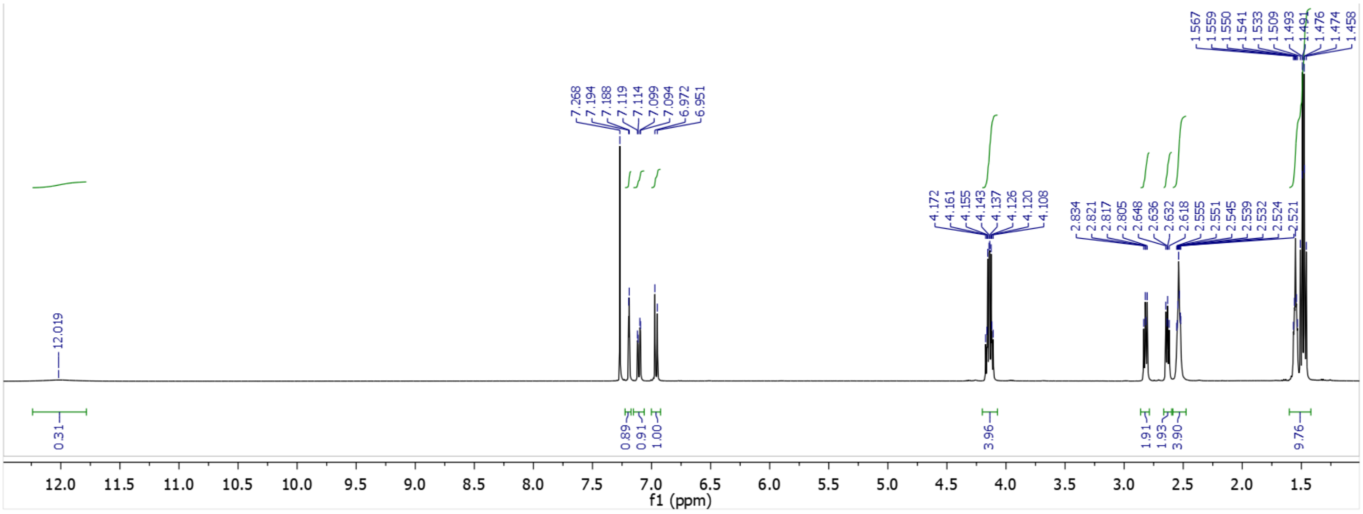

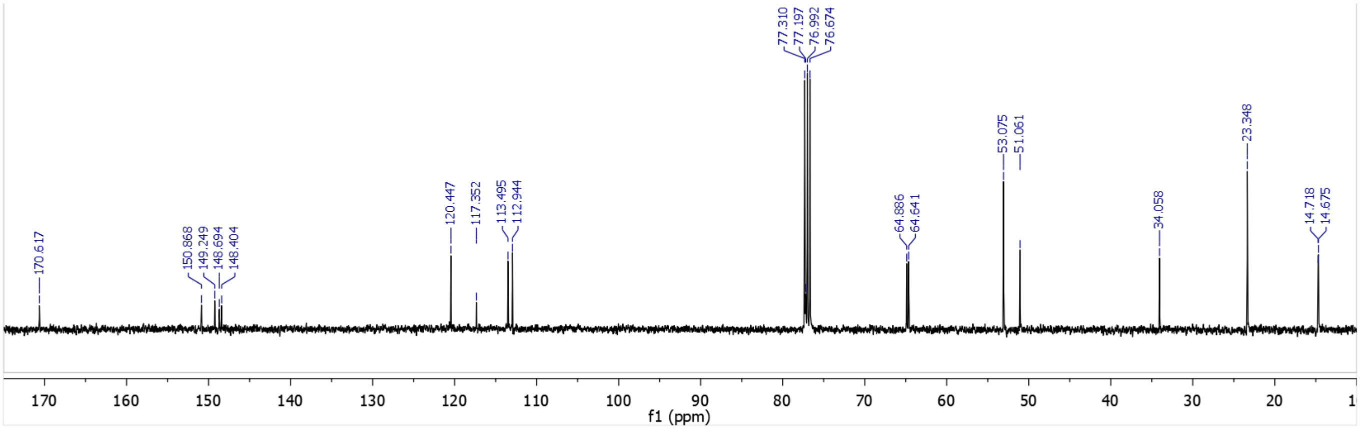

Figure S22. 1H NMR at 400 MHz and 13C NMR at 100 MHz spectra for compound **35**

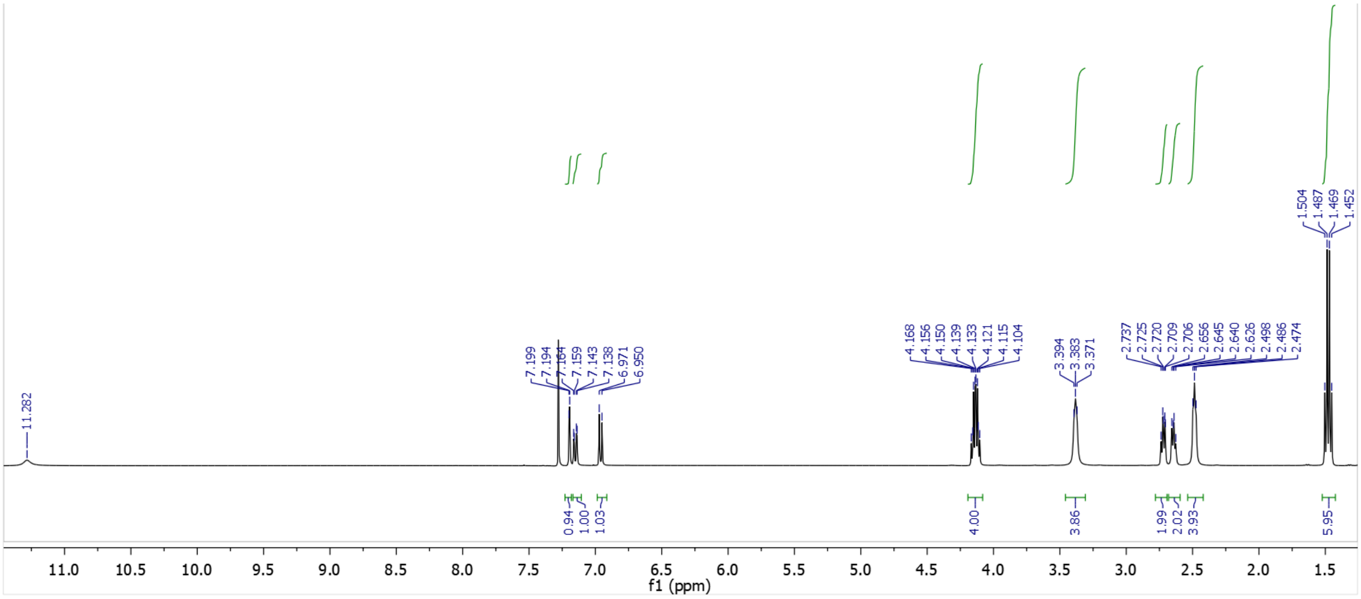

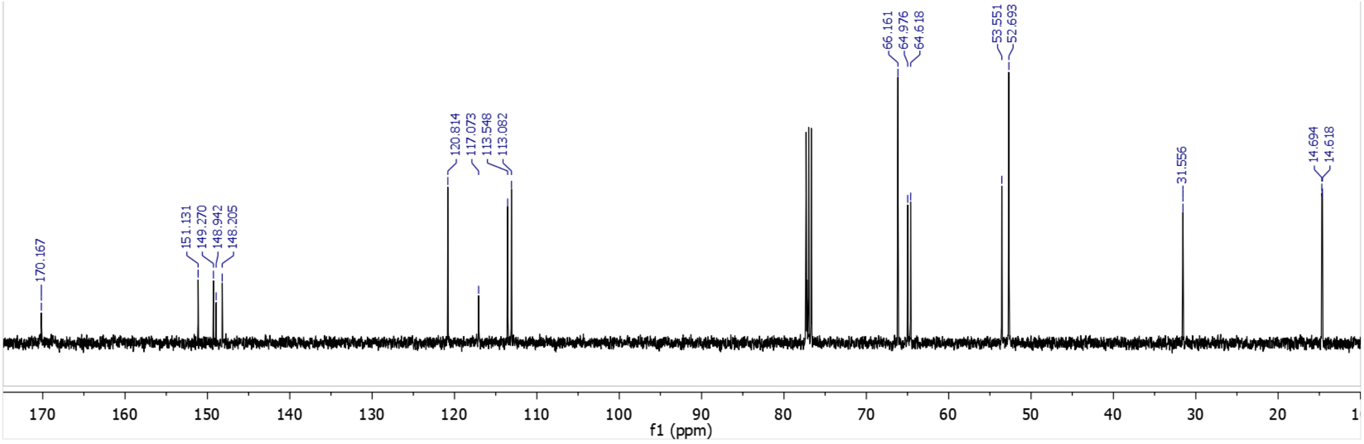

Figure S23. 1H NMR at 400 MHz and 13C NMR at 100 MHz spectra for compound **39**

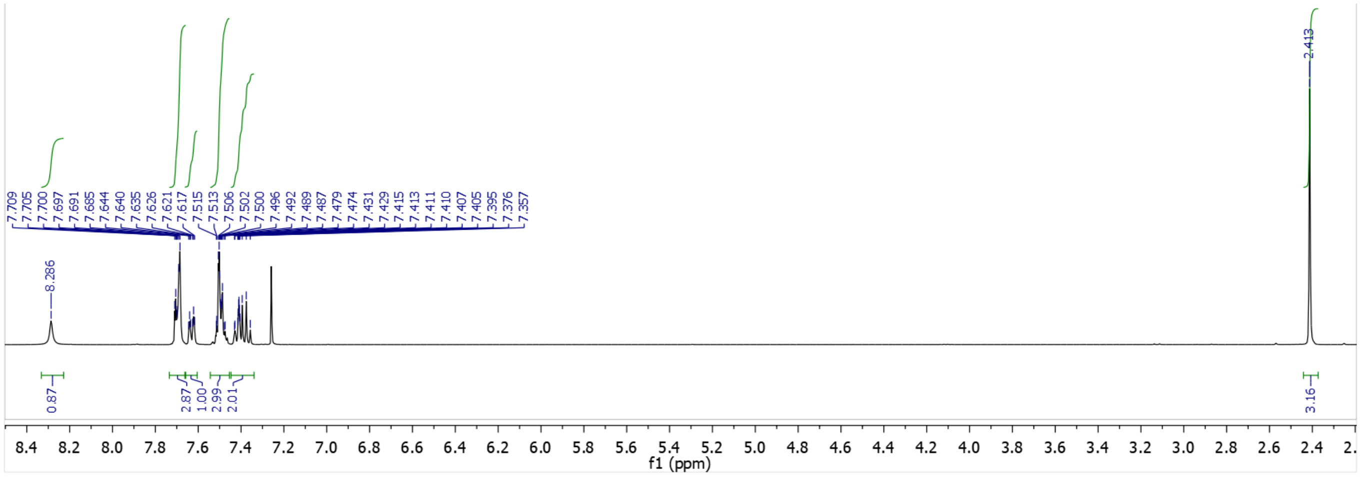

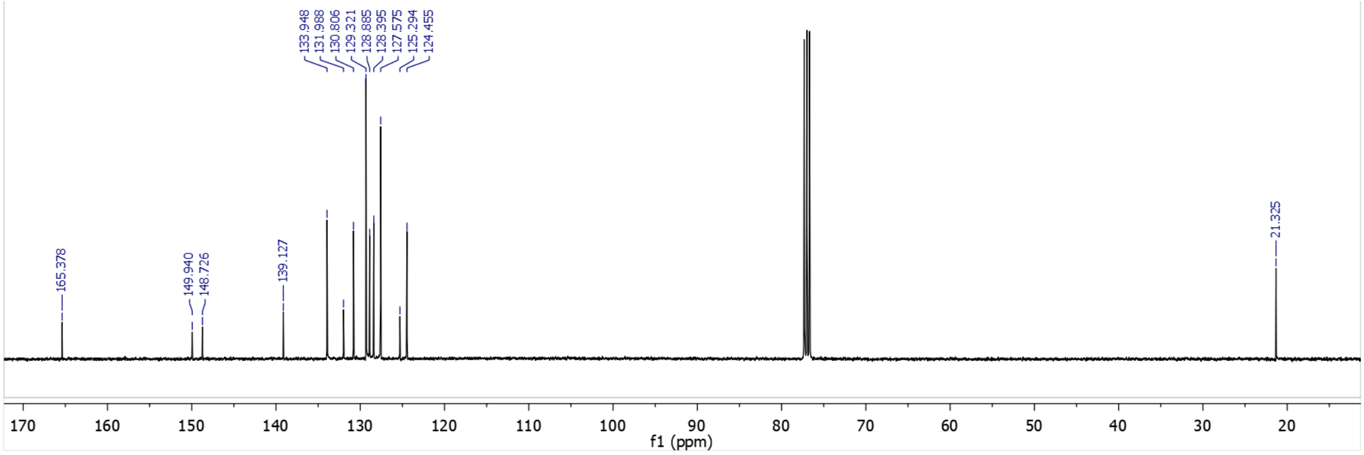

Figure S24. Dose-response curves of compound **1** against *P.f.* NF54, *P.f.* K$_1$ and L-6 cells.

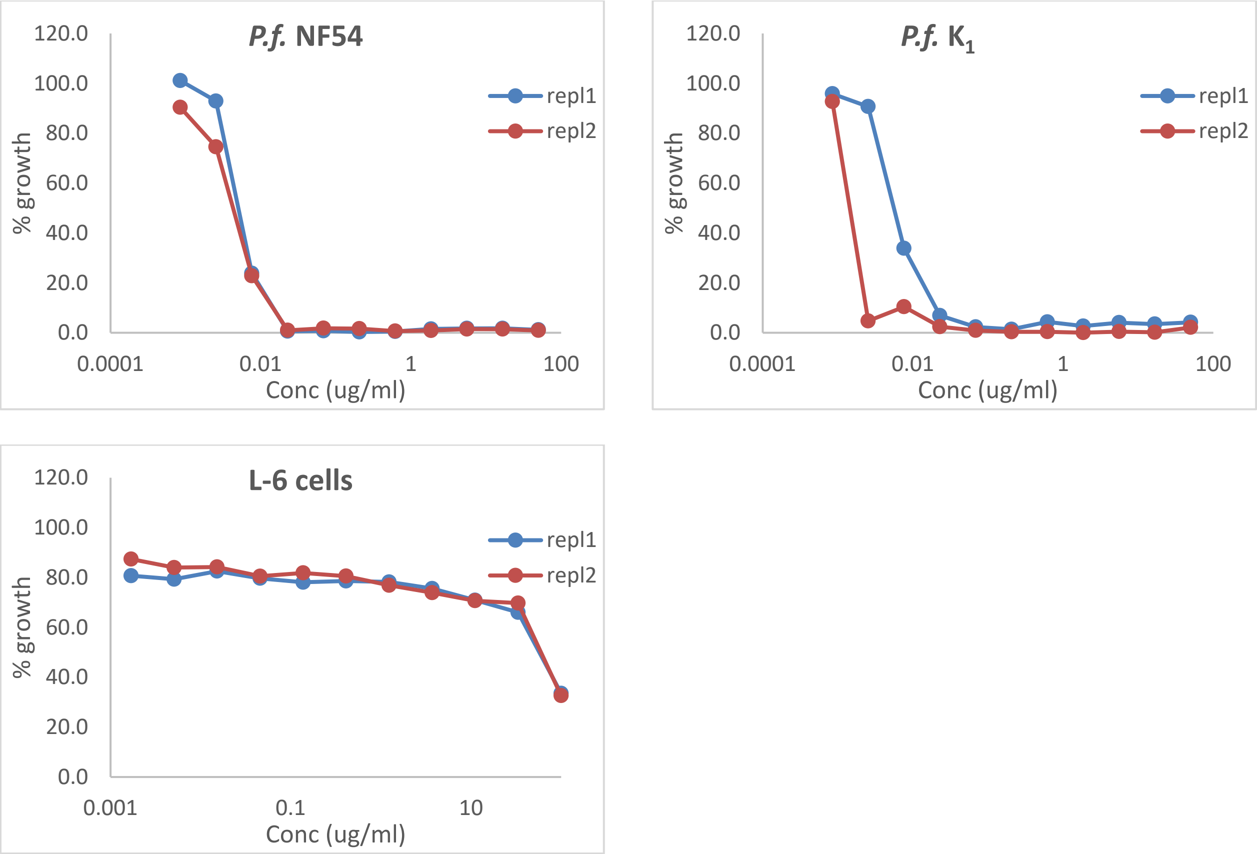

Figure S25. Dose-response curves of compound **10** against *P.f.* NF54, *P.f.* K$_1$ and L-6 cells.

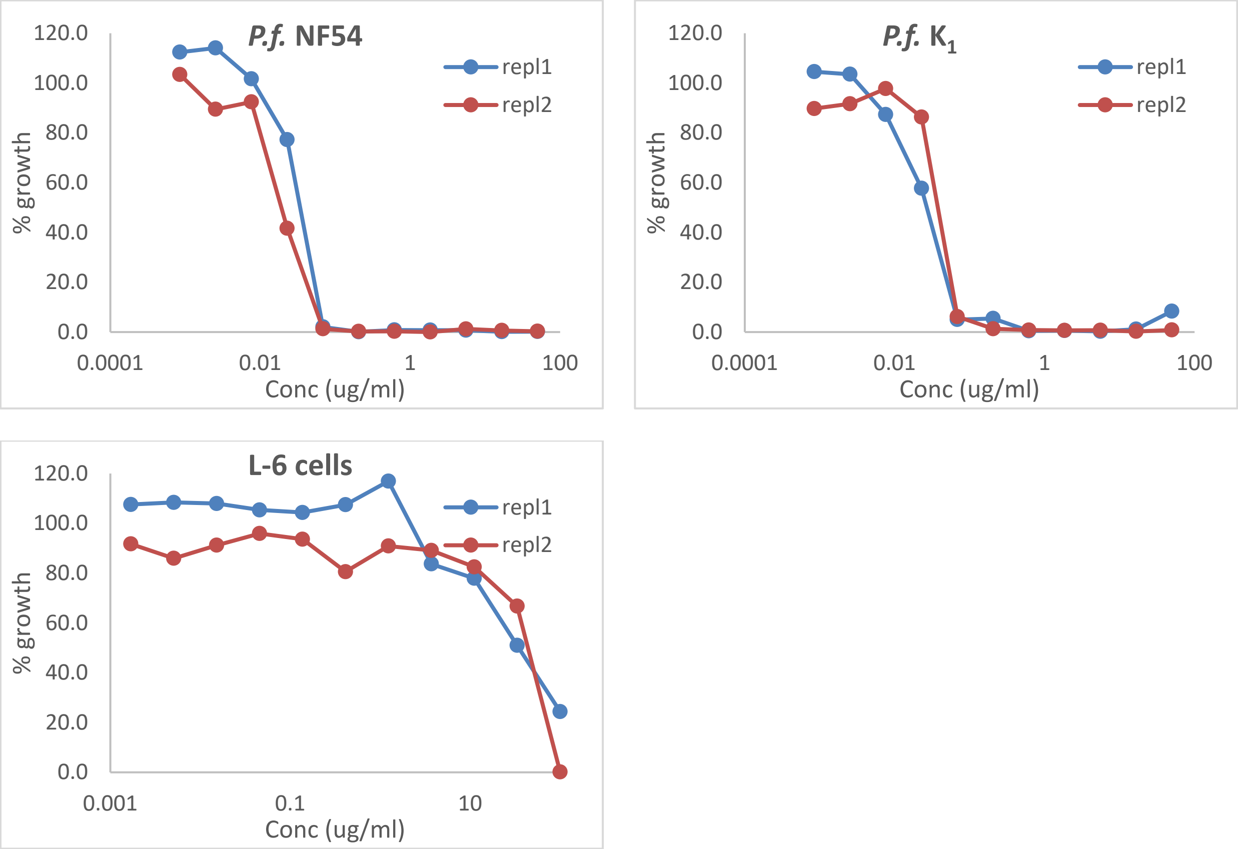

Figure S26. Dose-response curves of compound **11** against *P.f.* NF54 and L-6 cells.

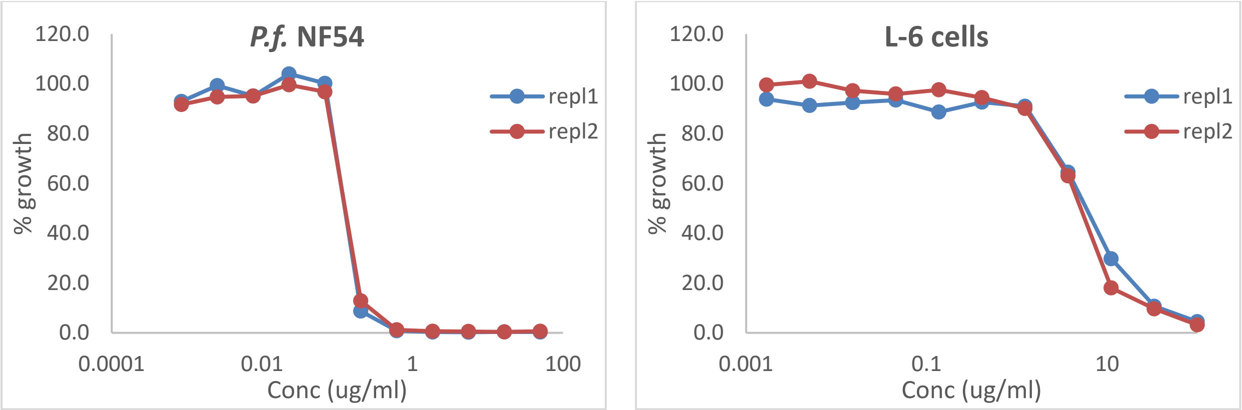

Figure S27. Dose-response curves of compound **12** against *P.f.* NF54 and L-6 cells.

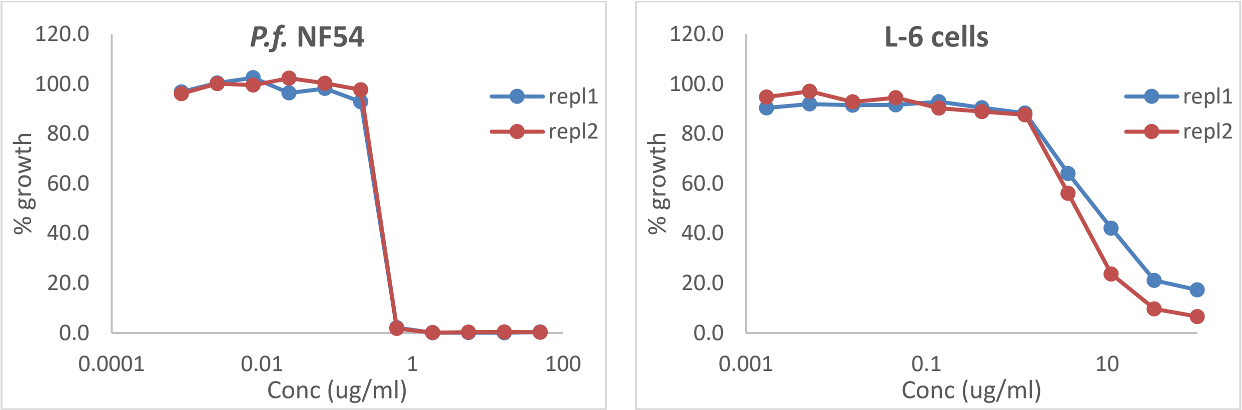

Figure S28. Dose-response curves of compound **13** against *P.f.* NF54, *P.f.* K1 and L-6 cells.

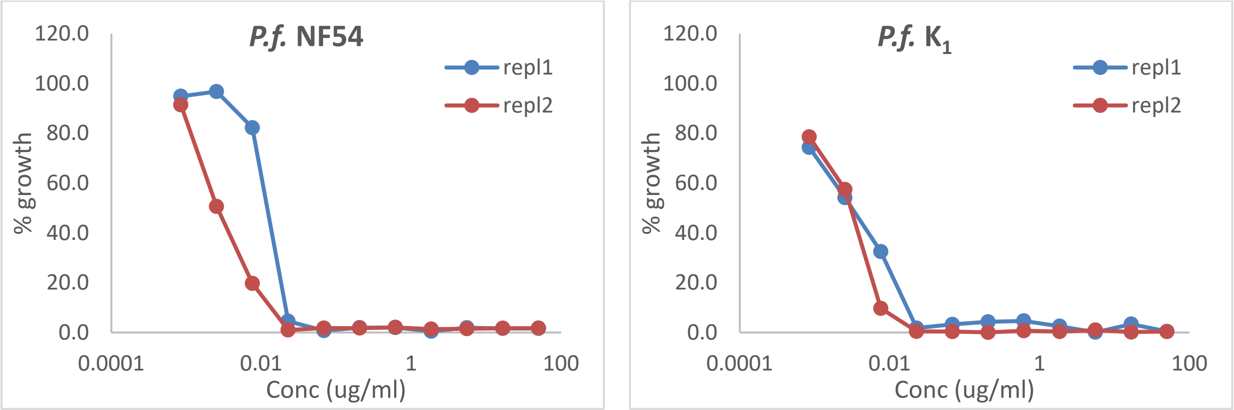

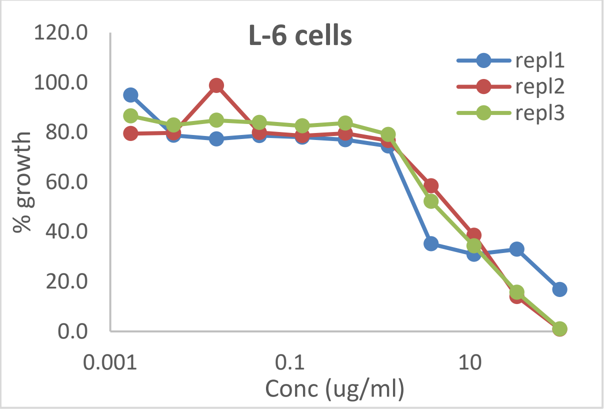

Figure S29. Dose-response curves of compound **14** against *P.f.* NF54, *P.f.* K1 and L-6 cells.

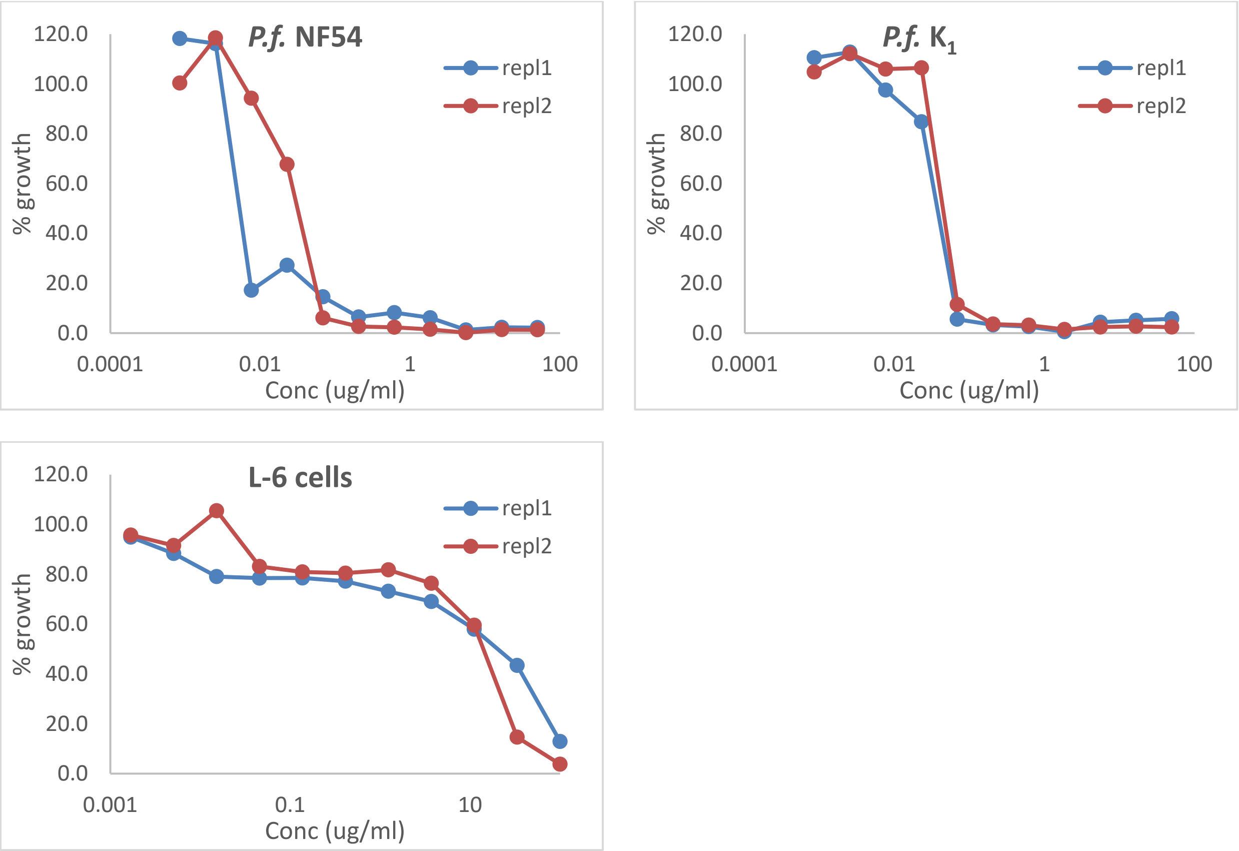

Figure S30. Dose-response curves of compound **15** against *P.f.* NF54 and L-6 cells.

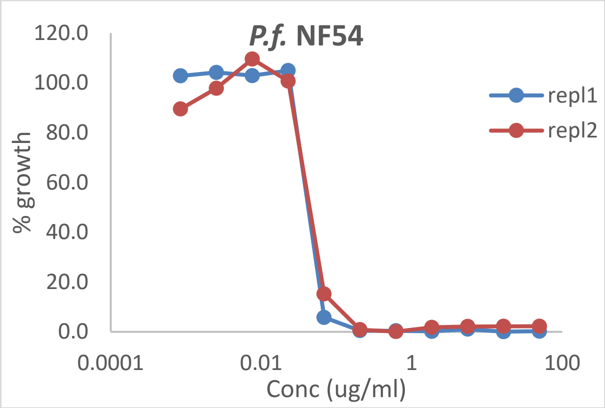

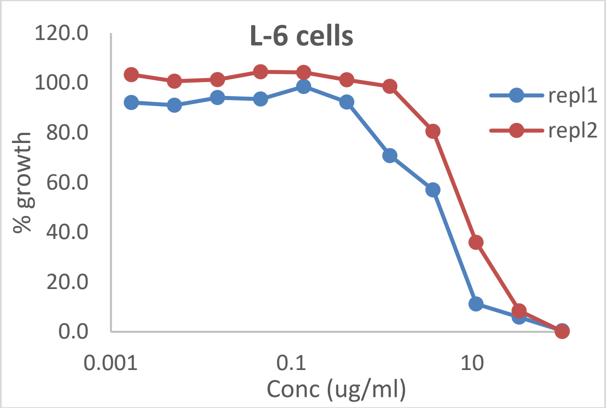

Figure S31. Dose-response curves of compound **16** against *P.f.* NF54, *P.f.* K1 and L-6 cells.

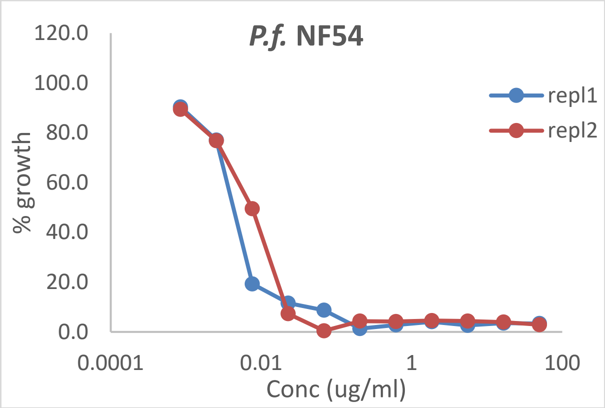

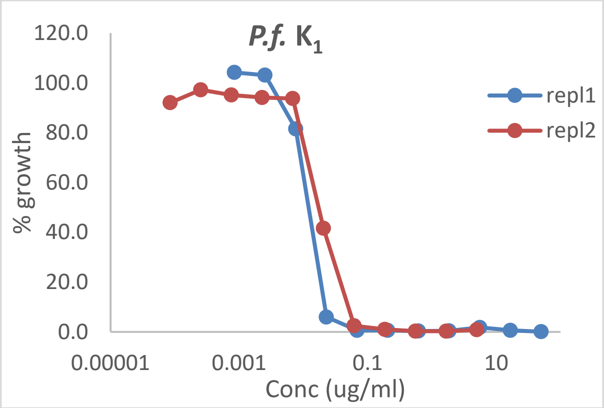

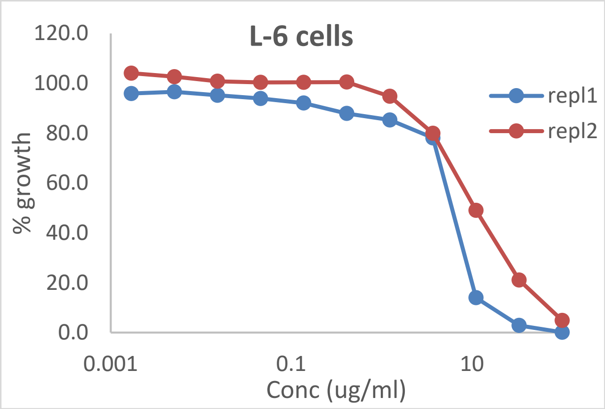

Figure S32. Dose-response curves of compound **17** against *P.f.* NF54, *P.f.* K1 and L-6 cells.

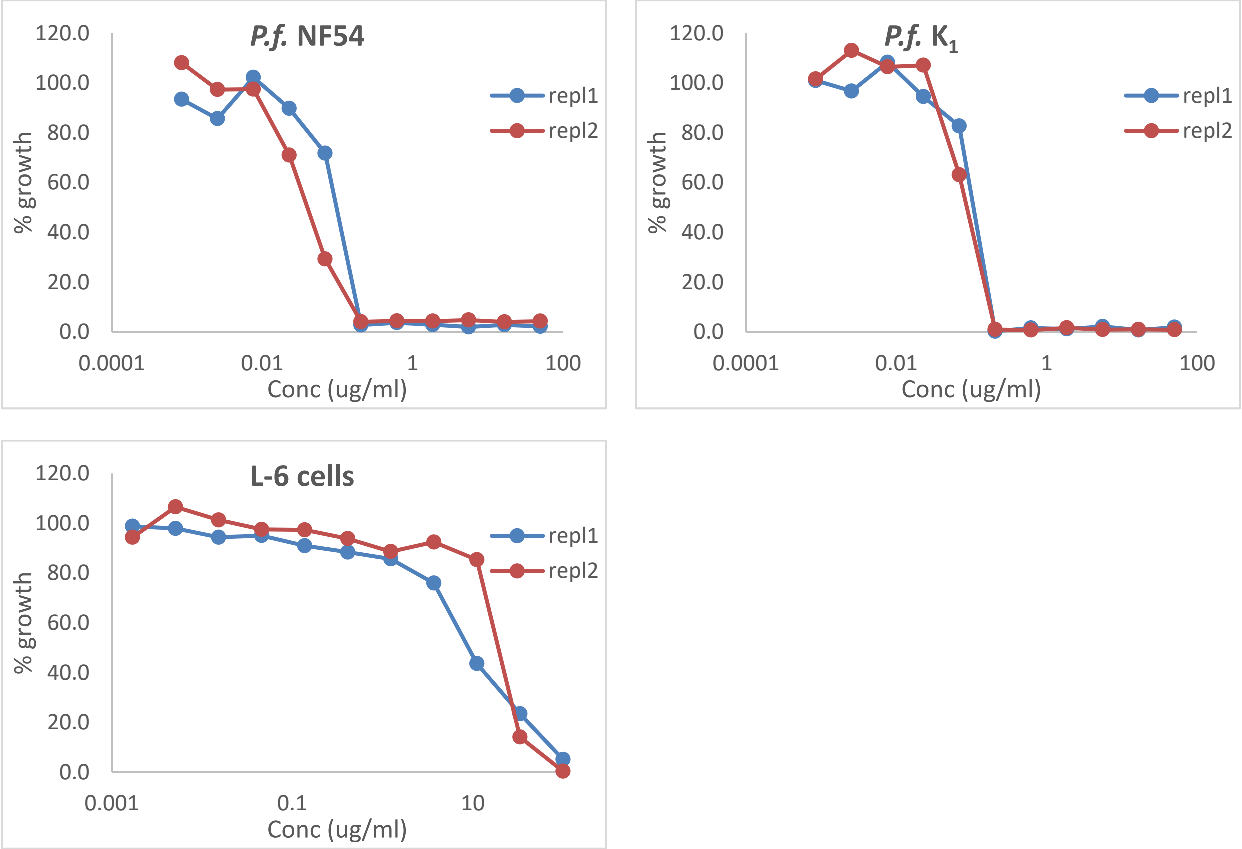

Figure S33. Dose-response curves of compound **26** against *P.f.* NF54, *P.f.* K1 and L-6 cells.

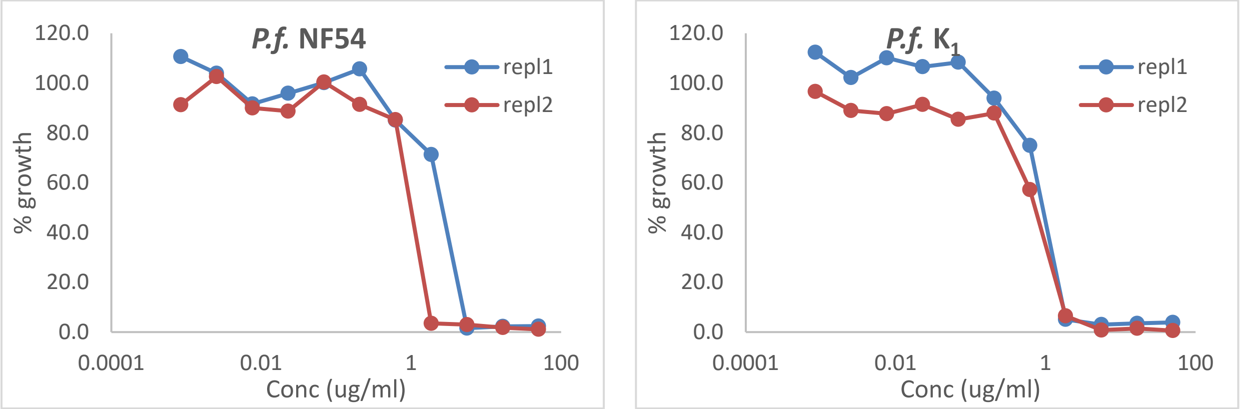

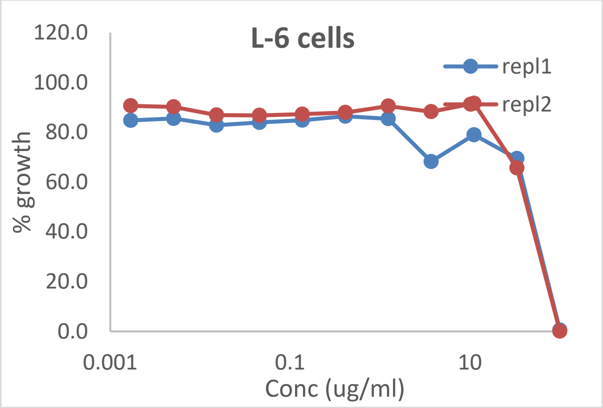

Figure S34. Dose-response curves of compound **27** against *P.f.* NF54, *P.f.* K1 and L-6 cells.

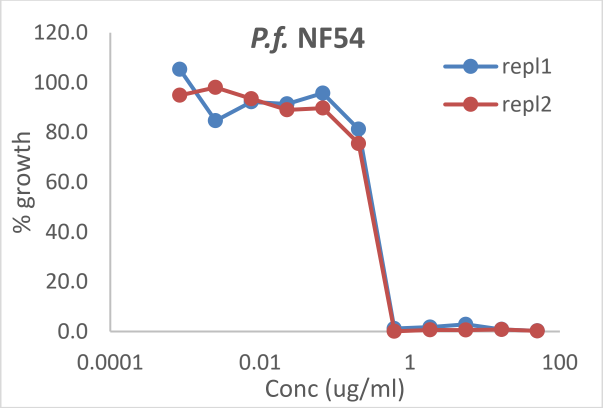

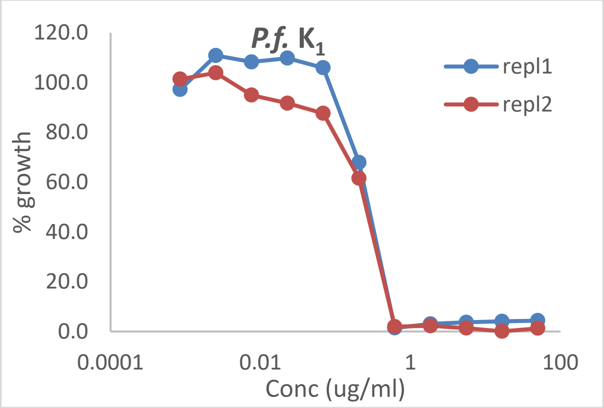

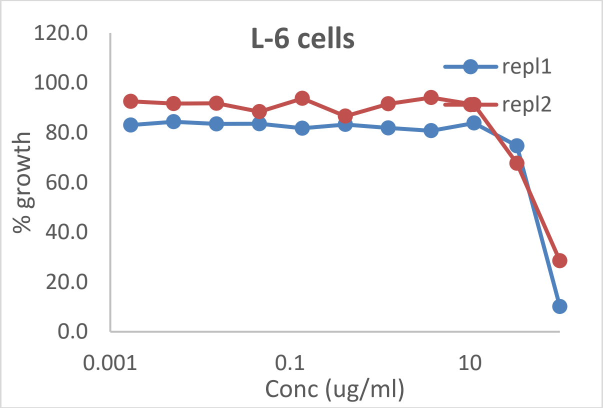

Figure S35. Dose-response curves of compound **28** against *P.f.* NF54 and L-6 cells.

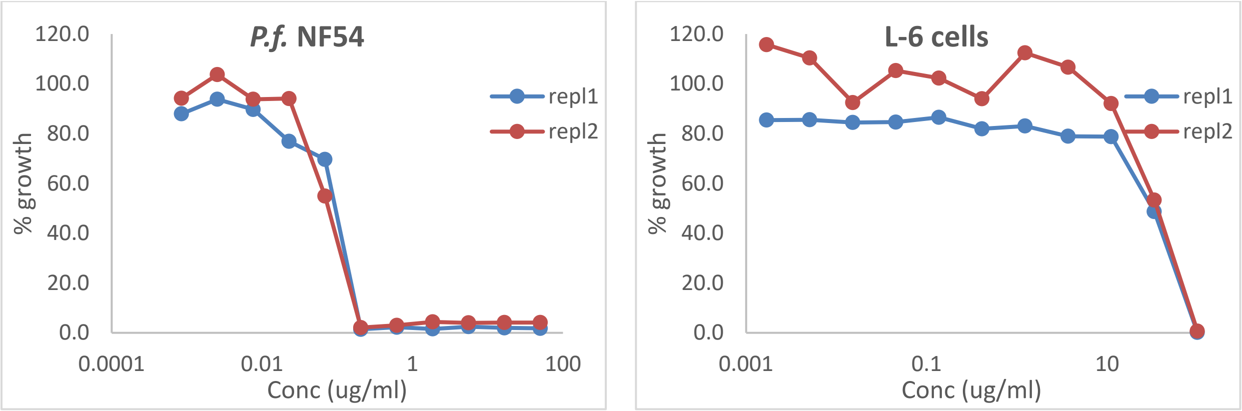

Figure S36. Dose-response curves of compound **29** against *P.f.* NF54 and L-6 cells.

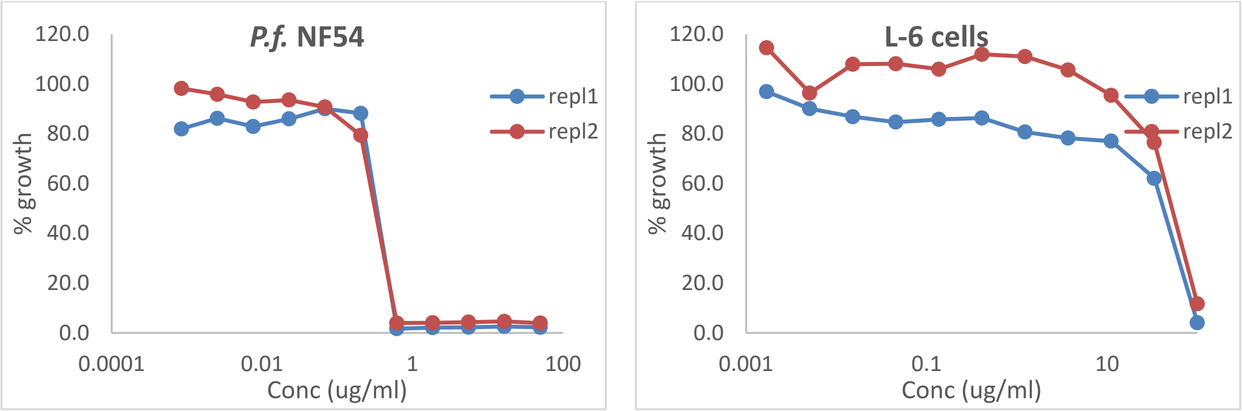

Figure S37. Dose-response curves of compound **31** against *P.f.* NF54, *P.f.* K1 and L-6 cells.

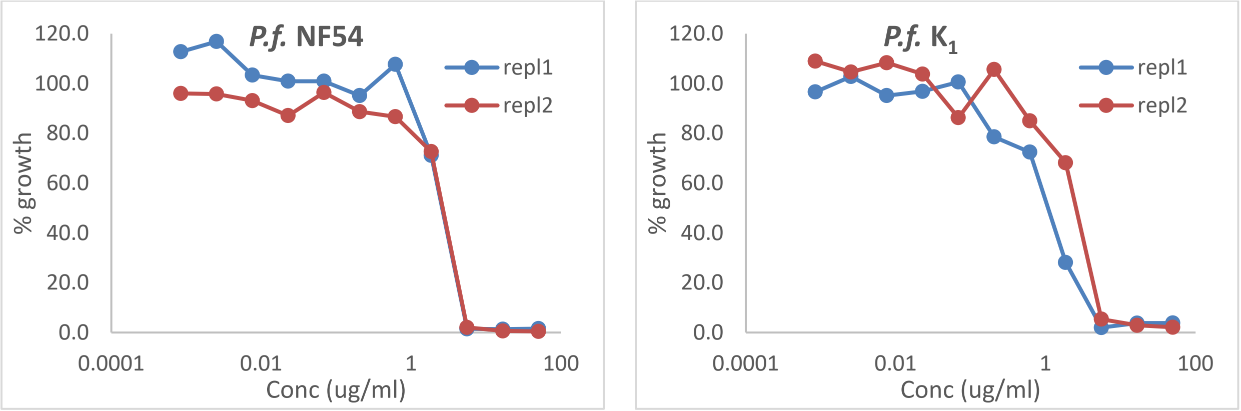

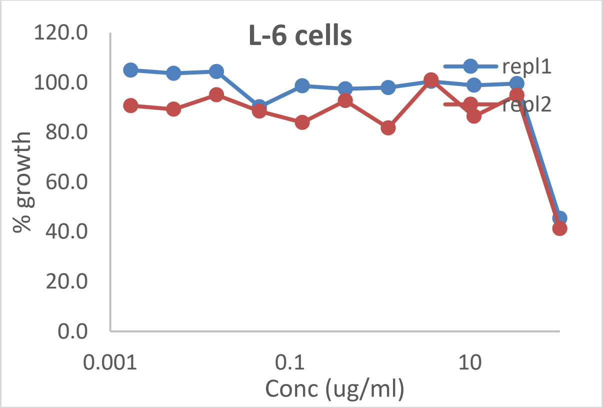

Figure S38. Dose-response curves of compound **32** against *P.f.* NF54, *P.f.* K1 and L-6 cells.

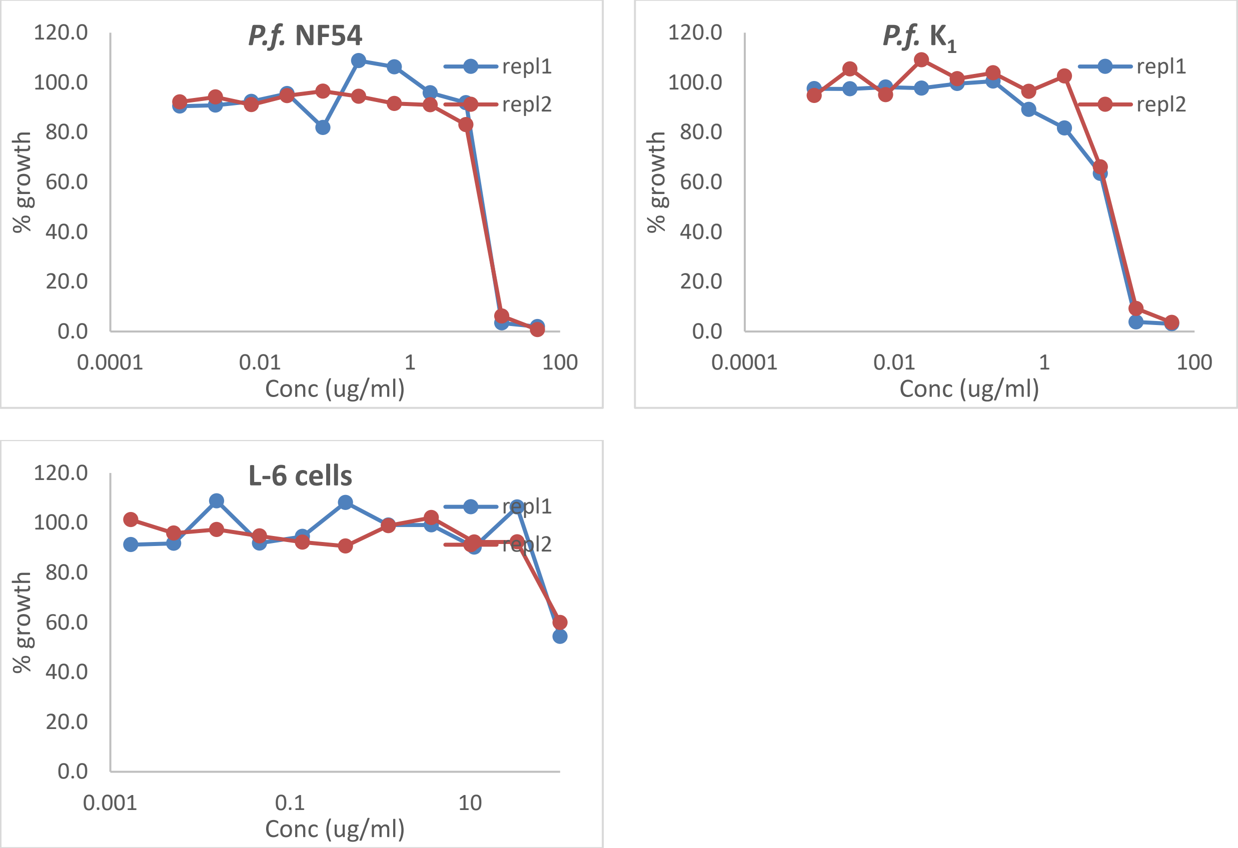

Figure S39. Dose-response curves of compound **34** against *P.f.* NF54, *P.f.* K1 and L-6 cells.

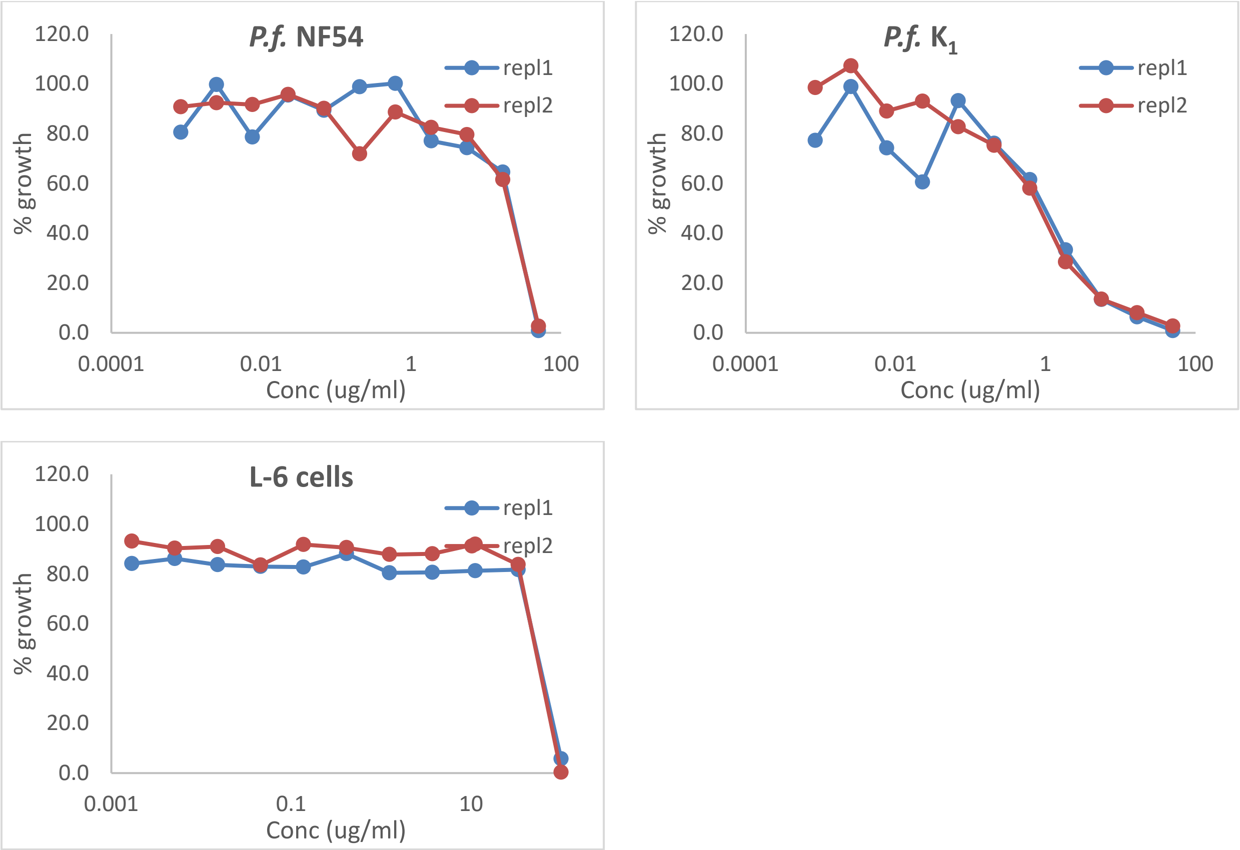

Figure S40. Dose-response curves of compound **35** against *P.f.* NF54, *P.f.* K1 and L-6 cells.

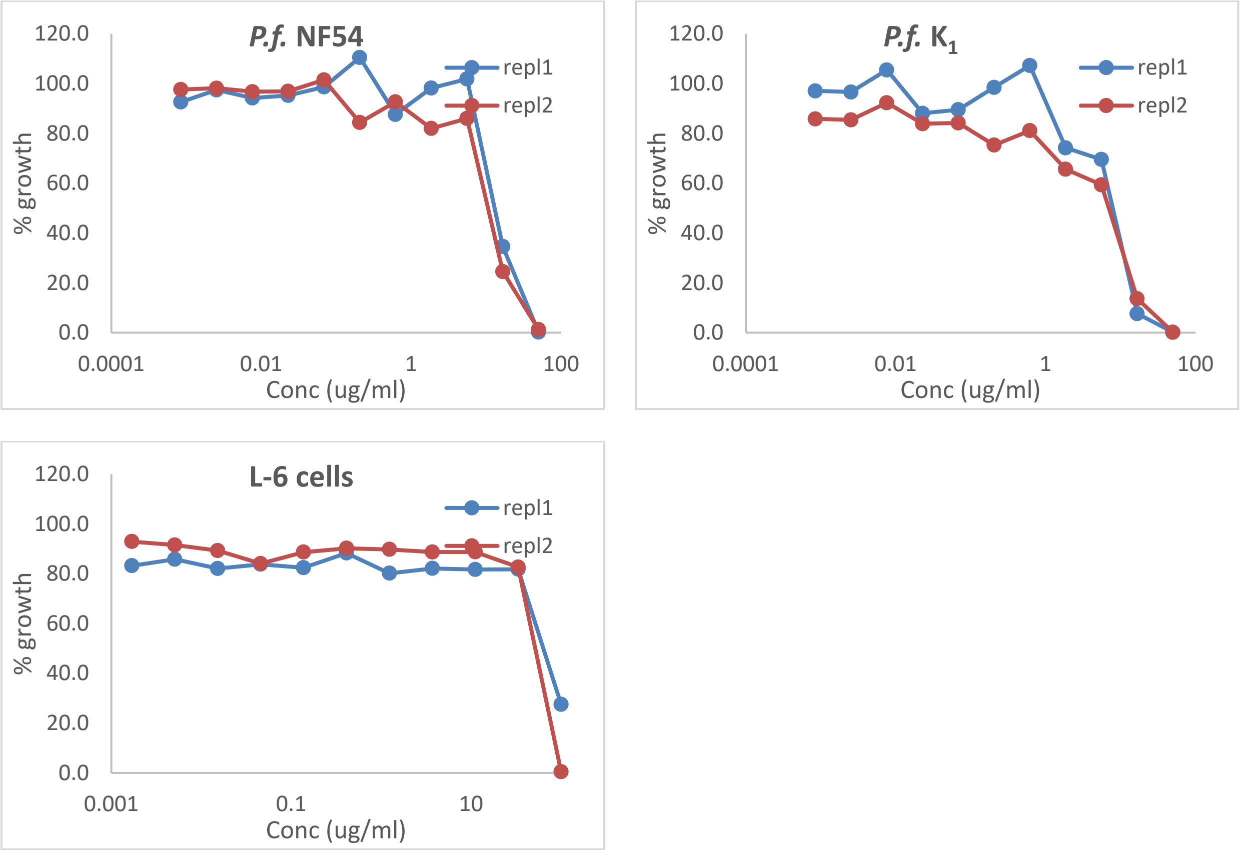

Figure S41. Dose-response curves of compound **39** against *P.f.* NF54, *P.f.* K_1 and L-6 cells.

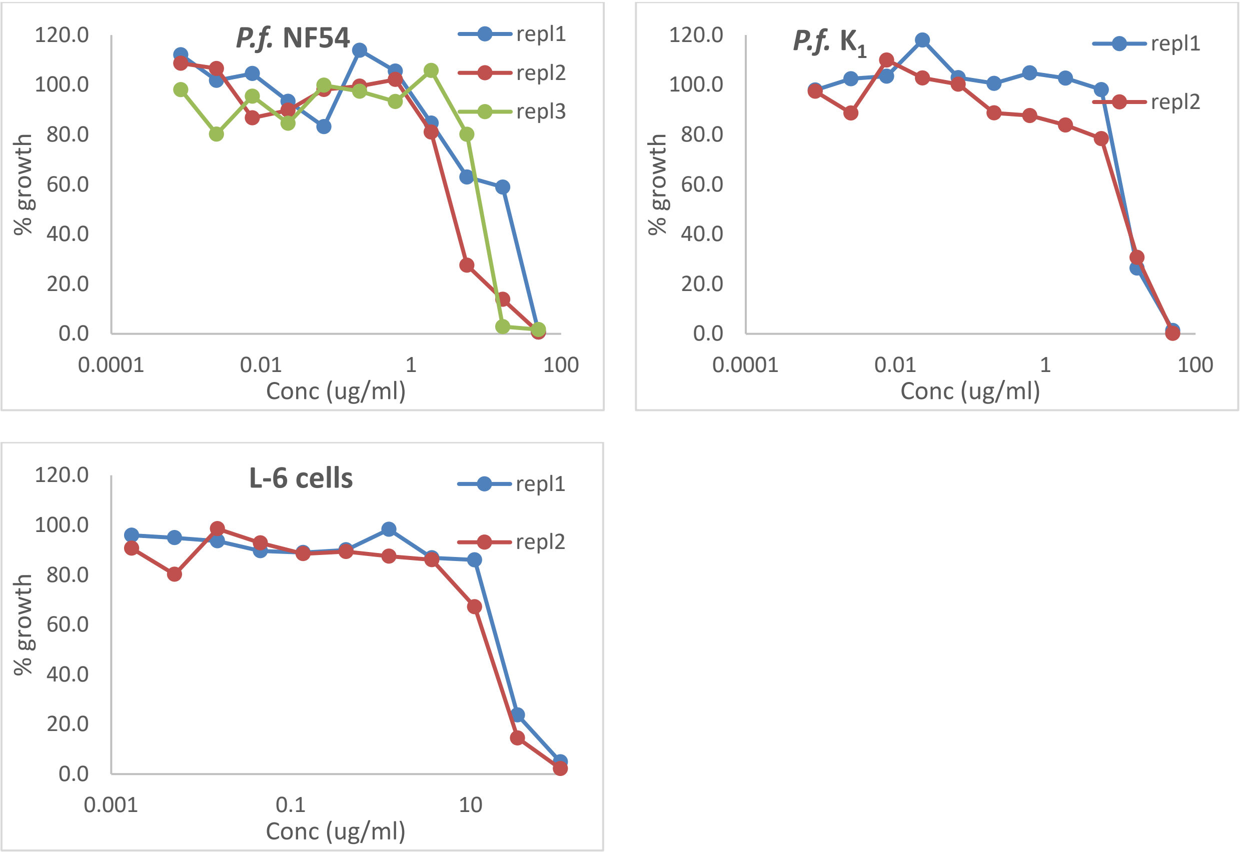

Table S1. Activities with standard deviation values of compounds **1, 10-17, 26-29, 31, 32, 34, 35** and **39** against *P.falciparum* NF54, *P.falciparum* K_1 and L-6 cells, expressed as IC_{50} (μM).[a]

**Compound**	***P.f.* NF54**[b]	***P.f.* K_1**[c]	**Cytotoxicity L-6 cells**
**1**	0.011 ± 0.001	0.011 ± 0.0068	159.3 ± 3.36
**10**	0.076 ± 0.021	0.091 ± 0.015	111.2 ± 12.9
**11**	0.343 ± 0.001		14.92 ± 1.05
**12**	0.831 ± 0.011		14.24 ± 3.45
**13**	0.019 ± 0.010	0.007 ± 0.00	9.97 ± 3.36
**14**	0.049 ± 0.035	0.108 ± 0.025	41.09 ± 9.96
**15**	0.098 ± 0.002		13.99 ± 3.97
**16**	0.014 ± 0.003		20.03 ± 5.67
**17**	0.167 ± 0.068		35.03 ± 12.7
**26**	4.055 ± 1.78	1.87 ± 0.210	101.7 ± 2.41
**27**	0.674 ± 0.015	0.586 ± 0.020	116.4 ± 4.35
**28**	0.192 ± 0.020		77.23 ± 4.46
**29**	0.712 ± 0.013		104.0 ± 11.4
**31**	7.24 ± 0.068	5.02 ± 2.03	242.3 ± 10.3
**32**	24.25 ± 0.575	19.53 ± 0.610	265.7 ± 0.00
**34**	56.01 ± 0.821	2.41 ± 0.179	139.8 ± 0.995
**35**	30.15 ± 3.05	18.99 ± 1.15	147.3 ± 15.1
**39**	21.52 ± 8.99	39.92 ± 1.64	65.95 ± 9.06
**ART**		0.0064	450.5
**CQ**		0.15	188.5
**POD**			0.012

ART = artemisinin; CQ = chloroquine; POD = podophyllotoxin

[a] Values represent the average of four determinations (two determinations of two independent experiments)

[b] Sensitive to chloroquine

[c] Resistant to chloroquine and pyrimethamine.
